# Pro-dopaminergic pharmacological interventions for anhedonia in depression: a living systematic review and network meta-analysis of human and animal studies

**DOI:** 10.1016/j.ebiom.2025.105967

**Published:** 2025-10-29

**Authors:** Edoardo G. Ostinelli, Georgia Salanti, Malcolm Macleod, Virginia Chiocchia, Katharine A. Smith, Argyris Stringaris, James Downs, Emma SJ. Robinson, Gin S. Malhi, Dominic M. Dwyer, Astrid Chevance, Christoph U. Correll, Thomy Tonia, Emily Wheeler, Toshi A. Furukawa, Diego A. Pizzagalli, Michael Browning, Jennifer Potts, Andrea Cipriani

**Affiliations:** a Department of Psychiatry, University of Oxford, Oxford, UK; b NIHR Oxford Health Clinical Research Facility, Oxford Health NHS Foundation Trust, Warneford Hospital, Oxford, UK; c Oxford Precision Psychiatry Lab, NIHR Oxford Health Biomedical Research Centre, Oxford, UK; d Institute of Social and Preventive Medicine, University of Bern, Switzerland; e Centre for Clinical Brain Sciences, University of Edinburgh, UK; f Faculty of Brain Sciences, University College London, UK; g First Department of Psychiatry, Aiginiteion Hospital, National and Kapodistrian University of Athens, Greece; h MQ Mental Health Research, London, UK; i School of Physiology, Pharmacology & Neuroscience, University of Bristol, UK; j Academic Department of Psychiatry, Kolling Institute, Northern Clinical School, Faculty of Medicine and Health, The University of Sydney, Australia; k CADE Clinic and Mood-T, Royal North Shore Hospital, Northern Sydney Local Health District, St Leonards, Australia; l School of Psychology, Cardiff University, Cardiff, UK; m Université Paris Cité and Université Sorbonne Paris Nord, Inserm, INRAE, Center for Research in Epidemiology and StatisticS (CRESS), Paris, France; n Centre d’Epidémiologie Clinique, AP-HP, Hôpital Hôtel Dieu, F-75004, Paris, France; o Department of Psychiatry, Zucker Hillside Hospital, Northwell Health, New York, USA; p Department of Child and Adolescent Psychiatry, Charité Universitätsmedizin Berlin, Germany; q Kyoto University Office of Institutional Advancement and Communications, Kyoto, Japan; r Noel Drury, M.D. Institute for Translational Depression Discoveries, University of California, Irvine, CA, USA

**Keywords:** Anhedonia, Major depression, Dopamine, Living systematic review, Meta-analysis

## Abstract

**Background:**

It is unclear whether pro-dopaminergic drugs reduce anhedonia in major depressive disorder (MDD) and further, if so, to what extent this is the case.

**Methods:**

With lived experienced experts we co-produced two living systematic reviews of randomised controlled trials (RCTs) investigating the relative efficacy of pro-dopaminergic interventions in reducing symptoms of anhedonia in people with MDD (versus placebo) and in relevant non-human animal models (versus vehicle control/no intervention). Multiple electronic databases were searched until June 9, 2024. The primary outcomes were subjective anhedonia symptoms in humans and sucrose preference test (a measure of reward sensitivity and proxy for anhedonia) in animals. We evaluated other important domains and clinical aspects closely related to anhedonia, such as reward/reinforcement tasks, anxiety symptoms, acceptability, tolerability, and adverse events. We performed pairwise meta-analyses separately for human and non-human studies. We also estimated the relative effects of pro-dopaminergic versus non-dopaminergic antidepressants in human studies on anhedonia and overall depressive symptoms using a series of random-effects network meta-analyses of both aggregate and patient-level data. A multidisciplinary panel of international experts (including people with lived experience) then interpreted the overall results and produced a list of recommendations via a triangulation process. This study is part of GALENOS (Global Alliance for Living Evidence in aNxiety, depressiOn, and pSychosis). PROSPERO registration: CRD42023451821.

**Findings:**

Pro-dopaminergic interventions were associated with a small reduction of anhedonia symptoms (6 RCTs, n = 2076; SMD −0.24, 95% CI −0.46 to −0.03) in people with MDD and increased sucrose preference in animal models (27 RCTs; SMD 1.34, 0.88 to 1.79). We did not find data about reward/reinforcement tasks in humans. Evidence was rated as low to moderate. In the network meta-analysis, some antidepressants with a non-dopaminergic mechanism of action showed reduction in anhedonia symptoms, which was larger than pro-dopaminergic drugs and probably independent of overall depression improvement.

**Interpretation:**

Our findings provide some support for the role of dopamine in anhedonia. However, the precise neurobiological mechanisms of anhedonia in major depression are still poorly understood and we posit that they may be possibly related to altogether different or more general effects of antidepressants on these symptoms. Therefore, data on reward, including reward-related learning and memory, are needed to properly examine the relationship between dopamine modulation and anhedonia.

**Funding:**

10.13039/100004440Wellcome (GALENOS project).

**Research in context**

**Evidence before this study**

Anhedonia is a common feature of major depressive disorder, and in clinical practice it generally refers to a situation in which a person experiences a lack of general interest, and an ability to feel pleasure and joy from activities that would normally have provided enjoyment. Some studies suggest that increasing the levels of dopamine, a neurotransmitter in the brain, can reduce anhedonia in people with major depressive disorder. However, this is yet to be proven, and it is also unknown how pro-dopaminergic drugs (such as certain antidepressants or stimulants) target processes in the brain and body more generally that are related to anhedonia. We searched PubMed from database inception to March 31, 2025, for any meta-analyses of randomised controlled trials with an English language abstract, regardless of the full-text language, investigating the efficacy of pro-dopaminergic drugs in participants with major depressive disorder and anhedonia. We used the following search terms: (("Anhedonia"[Mesh]) OR "Depressive Disorder"[Mesh]) AND "Dopamine Agents"[Mesh]. Our search yielded 373 articles, however, only seven were systematic reviews and none of these was a living systematic review. The available papers were of limited use and scope, because they focused only on one medication or just a selected number of drugs, or on a specific sub-population of patients with comorbid depression (i.e., in people with also schizophrenia or with Parkinson's disease).

**Added value of this study**

This living systematic review explored the effects of pro-dopaminergic pharmacological interventions on anhedonia symptom severity and reward/reinforcement tasks in depression in both human and non-human studies. We carried out a series of pairwise and network meta-analyses using both aggregate and individual patient data. We developed a triangulation process to jointly appraise data from human and non-human sources of evidence to (i) interpret the results, (ii) infer about the mechanism of action of pro-dopaminergic interventions on anhedonia in depression, and (iii) agree on the implications for future research. Multiple stakeholders and lived experience experts were involved in co-producing this review. We found that the effects of pro-dopaminergic agents on anhedonia symptoms in humans were small. Moreover, there are non-dopaminergic agents that were more efficacious in their actions on anhedonia than pro-dopaminergic medications.

**Implications of all the available evidence**

Our results support the hypothesis that dopaminergic-based pathways are implicated in anhedonia, although the underlying neurobiological framework may be the result of complex interactions involving other neurotransmitters. For some drugs, the observed decrease in anhedonia symptom severity was likely specific and unrelated to their antidepressant effect. To properly investigate the role of dopamine and other neurotransmitters in anhedonia, a greater diversity of interventions (e.g., non-dopaminergic pharmacological treatments) and outcomes (including imaging data) should be considered in future trials and living systematic reviews.

## Introduction

Anhedonia is usually described as the inability to experience enjoyment from activities that would normally be pleasurable.^1^ It is a symptom of several psychiatric disorders and broadly manifests as markedly diminished interest in almost all activities.^2^ However, most commonly it features in major depressive disorder where along with low mood it is one of the two core diagnostic symptoms of the disorder, and clinically, anhedonia is associated with poorer response to treatment, worse quality of life, and impaired psychosocial functioning.^3^ The mesolimbic dopamine circuit is integral to broadly defined “reward processes” in the brain (including particularly reward learning, motivation and vigor) and clinical manifestations of anhedonia may reflect changes in this reward circuitry.^4^ Dopamine has also been shown to mediate behavioural reinforcement learning and reward prediction responses to conditioned stimuli in animals.^5^ For instance, studies have shown that mice responded with a higher preference to the sugar-water option in the sucrose preference test (SPT) when administered pro-dopamine agents. This demonstrates an increase in sensitivity to the reward, which may align with similar human data although studies using a human equivalent of the SPT are limited.

New treatments, such as kappa-opioid receptor antagonists, are currently being evaluated in Phase 2 and 3 studies,^6^ but targeting dopamine-related signalling in the brain is still the leading target to improve anhedonia-related symptoms in depression.^7^ Pro-dopaminergic drugs can also reduce anxiety, which is a common clinical feature in clinical studies with people with depression and anhedonia.^3^ However, how pro-dopaminergic treatments can improve anhedonia and related symptoms - and to what extent - has not been comprehensively investigated.^1^ Here, we aimed to summarise the best available evidence of efficacy of pro-dopaminergic agents for anhedonia from human and animal studies, and provide a broader context for future developments and drug discovery in anhedonia associated with depression.

## Methods

This review is a living systematic review (i.e. systematic reviews that are continually updated, incorporating relevant new evidence as it becomes available), which is part of GALENOS, a global evidence synthesis project co-produced with people with lived experience.^8^ For full information about the review methods, see the published protocol,^9^ also registered with PROSPERO (CRD42023451821). When new eligible studies are included, we will incorporate new evidence and update the living systematic reviews based on their potential impact on substantially changing the overall findings (all the different versions of the review will be published on the Wellcome Open Research Gateway for GALENOS, https://wellcomeopenresearch.org/galenos).

### Ethics

Ethical approval was not required for this systematic review and meta-analysis, as no primary data involving human or animal subjects were collected. The data and analyses are based on previously published research. We follow the Preferred Reporting Items for Systematic Reviews and Meta-Analyses (PRISMA) 2020 statement to report our analyses.^10^

### Search strategy and selection criteria

We searched for published and unpublished studies until 9th June 2024 (for full information on search terms, databases and list of interventions see https://osf.io/2d967). For humans, we included placebo-controlled randomised trials (RCTs) investigating the effects of pharmacological interventions with a recognised pro-dopaminergic mechanism of action in participants with unipolar MDD. In case of uncertainty on the eligibility of specific pro-dopaminergic interventions, we consulted with international experts in neuropharmacology to find an agreement. The list of potentially eligible pharmacological treatments is available in the review protocol^9^ and in Open Science Framework (https://osf.io/2d967). For animals, we included controlled experimental studies (parallel or crossover design) investigating pro-dopaminergic pharmacological interventions in any non-human mammalian species or zebrafish, which had an experimentally induced depression-like phenotype. Title and abstract screening and data extraction were performed by pairs of independent reviewers using Evidence for Policy and Practice Information Reviewer for human studies,^11^ and the Systematic Review Facility (RRID SCR_018907) for animal studies.^12^ Conflicts were resolved through discussion with a third reviewer.

### Outcomes

In human studies, the primary outcome was anhedonia symptom severity measured with observer- and/or self-rated anhedonia-specific scales (e.g., Snaith-Hamilton Pleasure Scale),^13^ or individual items from depression rating scales (e.g., item 8 from the Montgomery-Åsberg Depression Rating Scale (MADRS))^14^ after 8 weeks of treatment. Secondary outcomes included anxiety symptom severity, reward and reinforcement processes (e.g., probabilistic reward task, effort expenditure for rewards task),^15^ acceptability (i.e., the proportion of participant discontinuing treatment due to any cause), tolerability (i.e., the proportion of participants discontinuing the intervention due to adverse events), and safety (i.e., the proportion of participants reporting specific adverse events) at the end of the study.

In animal studies, the primary outcome was the change in a mode of anhedonic-related behaviour following dopaminergic manipulation, measured as sucrose preference in a SPT. The SPT requires an animal to detect, and select more, a solution containing a low concentration of sucrose (typically 1 or 2%) versus water; chronic stress reduced sucrose preference and/or consumption and this is reversed by antidepressant treatment. Secondary outcomes included dopamine-related neurobiological outcomes, such as dopamine concentration, dopamine receptor biology, dihydroxy-phenyl acetic acid (DOPAC, an active dopamine metabolite) concentration, dopamine/DOPAC ratio, and adverse events, where these were reported in publications which also reported results for the SPT.

### Risk of bias assessment

For human studies, we assessed the risk of bias with the Risk of Bias 2.0 tool.^16^ To evaluate biases due to missing evidence and biases across studies, we used the Risk of Bias for Missing Evidence (ROB-ME) tool.^17^ For animal studies, we assessed the risk of bias using SYRCLE's risk of bias tool.^18^ Reporting completeness in animal studies was evaluated according to the ARRIVE 2.0 guidelines.^19^

### Statistics

Effect sizes were calculated as standardised mean differences (SMDs) for continuous outcomes and odds ratios (ORs) for dichotomous outcomes and reported with 95% confidence intervals (CIs). We synthesised human data using random effects pairwise meta-analyses, with the restricted maximum-likelihood estimator for the heterogeneity variance (τ^2^) and the Hartung-Knapp method to adjust the 95% CIs, when there were at least five studies. For animal data used a multilevel mixed effects approach. We reported the heterogeneity using 95% prediction intervals, τ^2^ and the variance attributable to each of the nested hierarchical levels within the multilevel model. The extend of heterogeneity is represented by the width of the prediction intervals, which is the range within which the true effect of a new study is expected to be found. Where more than 10 treatment effects were available, we conducted meta-regression analyses to explore possible sources of heterogeneity. For human studies, we considered age of participants, anhedonia baseline score, sex assigned at birth (proportion of female participants), and planned treatment duration. For animal studies, we considered type, duration and dose of the intervention, type of disease model induction procedure (i.e., behavioural, pharmacological, surgical, and subtypes of these, e.g. type of behavioural model induction or timing of pharmacological induction) and route of administration. We also performed subgroup analyses by risk of bias tools and completeness of study reporting. We evaluated the sensitivity of the results from the meta-analysis of animal studies to the assumed within-study correlation coefficient, by using values 0.2 and 0.8 instead of our default 0.5. Where possible, we also used the normalised mean difference as effect size.

To compare the effects on anhedonia of pro-dopaminergic drugs with other antidepressants with a different presumed mechanism of action, we performed a series of post-hoc analyses: random effects network meta-analyses of antidepressant trials using an existing set of individual patient data (IPD) to estimate the comparative effect sizes of several antidepressants on the item 8 of the MADRS.^20^

We evaluated the certainty of the evidence provided by human and animal studies separately, the risk of within-study bias, across-study bias, indirectness, and other biases. We also make assumptions about the direction of bias in each domain (over- or under-estimation of the effect).

### Co-production with lived experience and triangulation

This living systematic review was co-produced with people with lived experience of mental disorders, who commented on the protocol design, interpreted the results, prepared the plain language summary, revised the manuscript, and will disseminate the findings to the wider public. The topic chosen was based on previous prioritisation exercises coordinated by the James Lind Alliance (https://www.jla.nihr.ac.uk/).

Triangulation is a process of evidence synthesis where sources of evidence with different types of bias are considered together.^9^ Systematic errors (or biases) are present in each source of evidence, but these biases are likely to be unrelated when different types of studies are assessed. Triangulation enables the integration and appraisal of evidence from different sources which would not usually be considered together. Triangulating animal and human evidence with different study designs can therefore be particularly helpful to examine the underlying mechanisms which underpin disorders and their treatment and is essential in the development of new treatment approaches.^21^ The authors of this paper were involved in the triangulation process, which included also an in-person meeting in London in June 2024. The input of multiple stakeholders (including basic human and animal researchers, clinical researchers, clinicians, and people with lived experience) throughout the whole review process depended on open and equitable communication, especially given the complexity of the topic and the novelty of the triangulation process.

### Role of funders

The funder had no role in study design, data collection, data analysis, data interpretation, or writing of the report. All authors approved the decision to submit for publication.

## Results

### Human studies

**Fig. 1:**
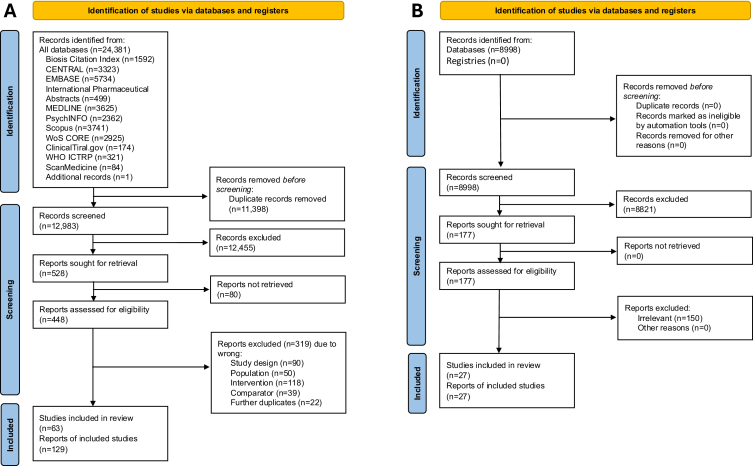
PRISMA flow diagram for human studies (A) and animal studies (B). FT: Full-text articles.

Searches of the eight databases and three trial registries resulted in 24,381 records. After the removal of duplicates and title/abstract screening, 528 reports were retrieved for full-text screening and finally 63 eligible trials (10,645 patients) were included in the review (Fig. 1; Appendix pp 9–16 for PRISMA diagram and full reference list, and pp 60–85 for Table of included studies). The mean age of participants was 42.2 years (SD 9.4, range: 15 to 72), 58% were female (range: 0–86%) and treatment duration was between four and 13 weeks (median: 6 weeks). The pro-dopaminergic interventions included (alphabetical order): amitifadine, brofaromine, bupropion, dextromethorphan-bupropion, gsk372575, isocarboxazid, memantine, methylphenidate, minaprine, moclobemide, phenelzine, pramipexole, ropinirole, rx-10100, selegiline, and tranylcypromine.

#### Anhedonia and reward/reinforcement processes

##### Data from the current living systematic review

**Fig. 2:**
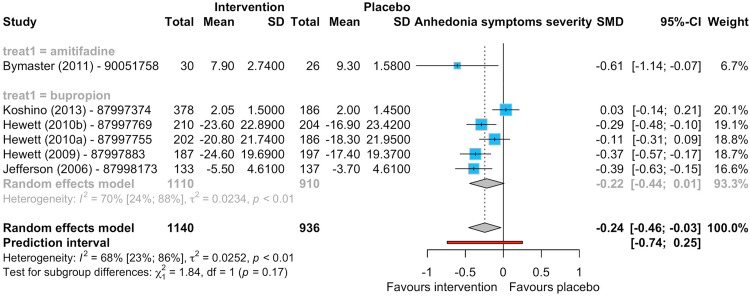
Forest plot for symptoms of anhedonia (primary outcome) comparing pro-dopaminergic interventions (namely, amitifadine and bupropion) vs placebo for individuals with anhedonia at 8 weeks. SMD: standardised mean difference, 95% CI: 95% confidence intervals, SD: standard deviation.

Only six RCTs in humans reported data on anhedonia (1140 participants allocated to bupropion (5 studies)^22^, ^23^, ^24^, ^25^, ^26^ or amitifadine (1 study)^27^ versus 936 to placebo). Three studies provided data using the Motivation and Energy Inventory, one trial used the IDS-C-30 Pleasure scale, and two RCTs used the MADRS (one used the MADRS anhedonia factor and one the MADRS item 8). Pro-dopaminergic interventions were associated with improved symptoms of anhedonia compared with placebo (SMD -0.24, 95% CI -0.46 to −0.03; Prediction Interval −0.74 to 0.25; 6 studies, 2076 participants), and the effect of bupropion alone was of very similar magnitude (SMD -0.22, −0.44 to 0.01; 5 studies, 2020 participants) (Fig. 2). The certainty of evidence was rated as low to moderate risk (see Appendix pp 49–58). We did not find data on reward and reinforcement processes in the context of pro-dopaminergic interventions for anhedonia. As only six studies were included in the meta-analysis of the primary outcome, we did not conduct any meta-regressions.

##### Comparisons with other network meta-analyses

In the network meta-analysis on MADRS item 8 from 34 studies (14,054 participants) comparing individual antidepressants versus placebo (Table 1), the effect of bupropion in this larger group of studies was smaller than the effect found in the aggregate data from the 5 RCTs (SMD -0.12, −0.25 to 0.00 vs −0.22, −0.44 to 0.01). Moreover, other antidepressants without a pro-dopaminergic mechanism of action were more effective in reducing symptoms of anhedonia when compared with placebo (Table 1). Using the GRISELDA dataset,^28^ we compared the effect of individual antidepressants specifically on anhedonia (i.e., item 8 of the MADRS) with the overall effect on depressive symptoms (i.e., change or endpoint score using standardised rating scales) (Table 1). There is no clear pattern of symptom effects being mapped onto pharmacological action. For some drugs the two effects were similar (duloxetine, escitalopram, fluoxetine, paroxetine, venlafaxine and vortioxetine), but for other antidepressants the anti-anhedonia effect was larger than the antidepressant effect (reboxetine) or vice versa (agomelatine, amitriptyline, bupropion, citalopram and trazodone).

**Table 1:** Network meta-analysis for symptoms of anhedonia comparing antidepressants vs placebo for individuals with anhedonia at 8 weeks.

Antidepressants	Pharmacological domain [based on neuroscience based nomenclature∗]	“Inability to feel” MADRS item [34 RCTs, individual-level data]	Overall MADRS score [391 RCTs, aggregate-level data]
		SMD (95% CI)	SMD (95% CI)
*Agomelatine*	Melatonin, serotonin	−0.05 (−0.19, 0.08)	**−0.26 (−0.33, −0.19)**
*Amitriptyline*	Histamine, serotonin, norepinephrine	−0.09 (−0.78, 0.60)	**−0.48 (−0.55, −0.41)**
*Bupropion*	Dopamine, norepinephrine	−0.12 (−0.25, 0.00)	**−0.25 (−0.33, −0.16)**
*Citalopram*	Serotonin	**−0.16 (−0.28, −0.05)**	**−0.24 (−0.31, −0.17)**
*Duloxetine*	Serotonin, norepinephrine	**−0.40 (−0.48, −0.32)**	**−0.37 (−0.44, −0.31)**
*Escitalopram*	Serotonin	**−0.21 (−0.39, −0.03)**	**−0.29 (−0.35, −0.24)**
*Fluoxetine*	Serotonin	**−0.28 (−0.46, −0.11)**	**−0.23 (−0.28, −0.19)**
*Paroxetine*	Serotonin	**−0.32 (−0.41, −0.23)**	**−0.32 (−0.37, −0.28)**
*Reboxetine*	Norepinephrine	**−0.39 (−0.74, −0.04)**	**−0.17 (−0.26, −0.08)**
*Trazodone*	Serotonin	−0.11 (−0.36, 0.15)	**−0.29 (−0.40, −0.17)**
*Venlafaxine*	Serotonin, norepinephrine	**−0.30 (−0.47, −0.13)**	**−0.33 (−0.39, −0.28)**
*Vortioxetine*	Serotonin	**−0.31 (−0.42, −0.19)**	**−0.28 (−0.36, −0.20)**
*PLACEBO*	-	*Reference*	*Reference*

95 % CI: 95% confidence interval; MADRS: Montgomery-Åsberg Depression Rating Scale; RCT: randomized controlled trial; SMD: standardised mean difference.

Statistically significant results are highlighted in bold.

∗ For full information about Neuroscience based Nomenclature, see https://nbn2r.com/.

#### Anxiety symptoms, acceptability, tolerability and specific adverse events

**Fig. 3:**
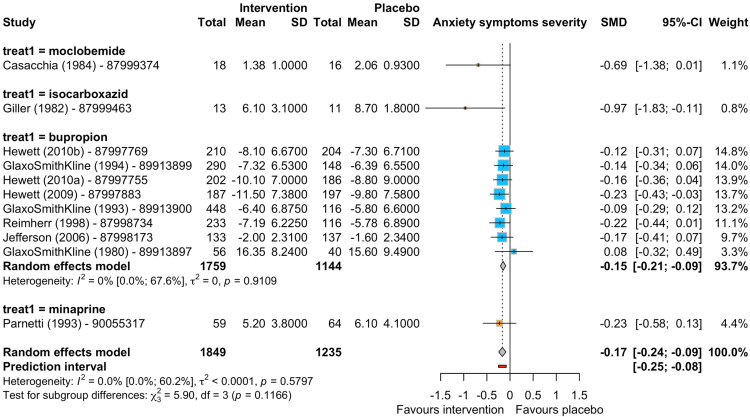
Forest plot for symptoms of anxiety comparing pro-dopaminergic interventions vs placebo for individuals with anhedonia at 8 weeks. SMD: standardised mean difference, 95% CI: 95% confidence intervals, SD: standard deviation.

Eleven studies contributed data about anxiety symptoms (1849 participants allocated to pro-dopaminergic interventions, 1235 to placebo)^22^, ^23^, ^24^, ^25^^,^^29^, ^30^, ^31^, ^32^, ^33^, ^34^, ^35^ favouring pro-dopaminergic interventions (SMD −0.17, −0.24 to −0.09; Prediction Interval −0.25 to −0.08) (Fig. 3). In terms of number of participants discontinuing treatment due to any reason (acceptability), we found no difference between pro-dopaminergic interventions and placebo (OR 0.97, 0.79 to 1.17; 52 studies) (Appendix pp 23–25). The meta-analysis about discontinuation due to adverse events included 43 studies (9030 participants: 5110 allocated to pro-dopaminergic interventions, 3920 to placebo) and showed that placebo was better tolerated than active interventions (OR 1.83, 1.38 to 2.41) (Appendix pp 26–28). Placebo was favoured over the dopamine reuptake inhibitor interventions for all adverse events aside from vomiting, where the null effect was not excluded (see Appendix pp 29–48). There were enough data to conduct meta-regressions for age, proportion of females, and treatment duration for all specific adverse events but vomiting, which was only reported in 5 studies overall. Of these meta-regression analyses, only proportion of females was able to explain between-study variance for dry mouth with a higher proportion of females predicting less reports of dry mouth (OR 0.14, 0.03–0.76—Appendix p 46). Aside from vomiting, which was rated at moderate risk of bias, the summary of evidence for the other specific adverse events was rated as low risk (Appendix pp 49–58). For ROB-ME results, see Appendix (pp 86–99).

### Animal studies

The initial search of four databases identified 8998 unique studies, 27 of which met the inclusion criteria and were included in the review (20 studies used rats and seven studied mice - Fig. 1; see Appendix pp 101–109 for description of characteristics of included studies and pp 166–168 for references). All studies used a disease model and 18 employed some form of chronic, in some cases unpredictable, mild stress. Although there are many other behavioral methods used in animals to study reward processing, these were not associated with a depression model and therefore not included in this analysis.

#### Sucrose preference

For the primary outcome (sucrose preference), 64 experimental comparisons investigated the effects of 19 pro-dopaminergic agents (including amantadine, aripiprazole, bromocriptine, bupropion, cryptotanshinone, d-amphetamine, dopamine, l-DOPA, *P. orientalis* seed, piribedil, pramipexole, ropinirole, quinpirole, selegiline, simvastatin, SKF83959, SKF38393, tranylcypromine and 2_HBC) in 10 animal strains (Appendix pp 110–116). The risk of bias was rated unclear for the great majority of studies, only 12 studies reported randomisation to the experimental group, whereas four reported blinding, two justified the sample size used, and none reported on whether any animals were excluded from the analysis (Appendix p 114).

Pro-dopaminergic interventions improved sucrose preference (SMD 1.34, 0.88–1.79) (Appendix p 115). There was no significant effect of sex, depression model used, route of intervention administration, whether the drug was administered before or after the depression modelling, duration of treatment, intervention used, or risk of bias (Appendix pp 116–123). In 61 of 64 comparisons, it was possible to calculate a normalised mean difference effect size, and the corresponding meta-analysis suggested that pro-dopaminergic drugs reversed about 70% of the change in sucrose preference which had been induced by the modelling of depression (Appendix pp 130–131).

For dopamine concentration, three studies reported 13 outcomes, and random effects meta-analysis suggested an increase in dopamine concentrations (SMD 1.63, 0.67 to 2.59) (Appendix p 134–136). For DOPAC concentration, dopamine/DOPAC ratio and dopamine receptor biology, there were insufficient studies to perform multilevel meta-analysis. We found evidence of small study effects (an indicator of potential publication bias) for the effects of dopaminergic agents on sucrose preference in depression models (coefficient 15.22, 95% CI 3.25–27.19) (Appendix p 149). Summary of evidence results are reported in the Appendix (pp 158–164).

### Triangulation

Results of the triangulation process are summarized in Table 2.

**Table 2:** Recommendations about the design and analysis of future studies on anhedonia in humans and animals.

In humans • Co-develop and validate rating scales (also with experts in animal models) to investigate translational mechanisms and assess different components of anhedonia in depression (for instance, anticipatory vs consummatory phases, or wanting vs liking). • Explore whether the severity of anhedonia at baseline predicts the response to dopaminergic and non-dopaminergic drugs and whether the effect on anhedonia is different to that on depression in general. • Consider whether pro-dopaminergic drugs might have a role as add-on treatments if they show a specific anhedonia effect. • Consider whether there are behavioural tasks (e.g., reward learning tasks or real-world behavioural readout) that we can measure in humans that are at least phenomenologically closer to the animal work. These need to be co-developed between human and animal researchers. • Evaluate other outcomes related to anhedonia and depression, for instance anxiety and quality of life.
In animals • Better define the phenotype(s) of depression in animals, in particular its behavioural manifestation. This should include consideration of deficits in reward processing vs. deficits in reward learning because the latter may be more universal across different aetiologies of depression (rather than just chronic stress-induced depression). • Utilise behavioural assessment tools (such as the rat reward learning assay, the rodent probabilistic learning tasks, the taste reactivity test and the microstructural analysis of licking) as tests of hedonic reactions in rodents that directly link to the “wanting/liking” difference noted in humans (see first bullet point above) and incorporate multiple measures of reward processing rather than the single domain tested using the sucrose preference test. • Find and/or use assessment methods which reflect other potential deficits in reward processing more relevant to anhedonia and look at more than one disease model in the same study. • Strongly encourage pre-registration of animal studies for hypothesis testing animal studies, as well as the open archiving of data and methods (including analytical tools), to reduce the risk of bias in analysis and publication.

Equal partnerships with people with lived experience of mental health conditions are central to GALENOS.^8^ For this study, two experts in lived experience formed part of the triangulation panel and participated in the triangulation of the sources of evidence. They were supported at the meeting by an MQ Mental Health Research team member with a background in co-production approaches in mental disorders. To prepare for the triangulation meeting, as well as review all the materials provided to the other triangulation panel members, the two experts in lived experience also met with the GALENOS Director to understand the scientific details of the studies assessed and to ask questions to clarify any areas where technical terms or jargon might prevent understanding of the results.

## Discussion

In this living systematic review and meta-analysis, we assessed the evidence from human and animal studies about the effects of pro-dopaminergic interventions on anhedonia in depression. We found that bupropion was more efficacious than placebo in reducing anhedonia symptoms at 8 weeks in people with depression, however, the effect size was small, the confidence interval was quite wide (almost reaching the null effect) and, interestingly, other antidepressants (with or without dopaminergic action) resulted in similar or possibly even greater improvements in anhedonia. These findings raise the question about the role of dopamine in improving anhedonia symptoms. Our results are consistent with dopamine playing a role in anhedonia, but also indicate that modulation of dopaminergic-based pathways is neither necessary nor sufficient for the treatment of anhedonia in humans and do not provide strong evidence that the effect of pro-dopaminergic drugs is greater than that seen in non-dopaminergic drugs. Unfortunately, the available evidence did not allow us to fully examine the underlying neurobiological framework of anhedonia in humans. While bupropion has a high affinity for dopamine transporters, it also exerts effects on other neurotransmitter systems, such as 5HT3A receptors and the noradrenaline transporter,^36^ suggesting indirect effects on dopamine neurotransmission which may relate to the therapeutic effect.

Evidence from animal models of depression and the SPT readout supported the reduction in anhedonia as the result of a complex interaction involving dopamine and non-dopamine neurotransmitters.^37^ Unfortunately, evidence from depression models and dopaminergic manipulations in more translational models were limited to only a small number of papers and therefore were not included in this analysis. Further evidence, particularly using innovative behavioural methods integrated with computational models, genetics and neuroimaging, would allow a better understanding of the dopamine-modulated reward pathway and how this relates to different aspects of reward processing and the symptom of anhedonia, including via indirect pathways.^1^ To better investigate the role of the dopaminergic system in anhedonia, a larger number and variety of interventions should be considered. There is evidence about novel treatments, such as kappa opioid receptor antagonists, which – even though the clinical trials have produced mixed results so far - may facilitate dopaminergic transmission and improve anhedonia by reducing synaptic peptide dynorphins,^38^ the potassium channel modulator ezogabine,^39^ and transcranial direct current stimulation of the left dorsolateral prefrontal cortex.^40^ Behavioural activation (improving recognition of avoidance patterns that contribute to the persistence of low mood and promote engagement in activities that offer positive reinforcement), positive affect treatment and augmented depression therapy (focusing on values clarification, behavioural activation, and problem-solving) are targeted psychotherapies, which place greater emphasis on the psychological mechanisms mediating reward and behavioural approaches, and which have shown promising results.^41^^,^^42^ We plan to incorporate these additional interventions into future iterations of this living systematic review.

In this project we used triangulation of evidence, an important and novel tool for mental health because it enables the integration and appraisal of evidence from different sources which would not usually be considered together because of their level of bias. By including clinical data from human studies and preclinical data, and with the voices of people with lived experience included throughout, the transdisciplinary process of triangulation used multiple sources of evidence to foster new conceptual models that bridge gaps between neuroscience, clinical expertise, and lived experience.^21^ At the end of the triangulation process, we agreed on a series of recommendations about the design and analysis of future studies about anhedonia in humans and animals (Table 2). We hope these recommendations will stimulate discussion among patients, clinicians, researchers and funders to better inform research prioritisation and future developments in the field, which in turn can provide new evidence to support clinical care.^43^

Anhedonia is a complex concept, as it involves reward learning and hedonic response (consummatory), and includes loss of interest (motivational), loss of pleasure, less energy (behavioural) and emotional numbing. Historically, it has been defined as an insensitivity to both physical and emotional pleasures, but neuroscience is now investigating it more as a motivational construct, focusing on dopamine and the anticipatory effect of “wanting” in addition to “liking” as well as the importance of reward-related cognition e.g. learning and memory, and its influence on these processes. It is not clear whether anhedonia is separate from a general loss of feeling all emotions, as in alexithymia (i.e., the inability or difficulty in recognizing, understanding, and describing one's own emotions), or purely the loss of enjoyment and pleasure. Clinically, people with anhedonia may still crave in a very hedonic fashion e.g. certain foods, and substances. Also, there is comorbidity between anhedonia/depression and drug abuse, or bulimia.^37^ Although data from animal studies included measurements of reward in the SPT, recent studies found that reward processing abnormalities do not reach levels that would be useful for clinical prediction in depressed patients.^44^ This lack of comparable data across animal species makes it difficult to draw robust conclusions about the biological mechanisms behind anhedonia. However, the available evidence does not preclude a possible causal role of reward processing in depression and anhedonia: higher reward-related activation in the ventral striatum is associated with better reward learning and lower anhedonic symptoms.^41^

The findings from animal studies should also be interpreted with some caution. We focused on the SPT because it is the most commonly used behavioural assessment method used in the context of examining anhedonia in animal models of depression. While the use and application of SPT in animal studies has been challenged,^45^^,^^46^ other assessment methods have been used in fewer studies and thus there are relatively few data (this includes the intracranial self-stimulation model and the back-translated probabilistic reward task and affective bias test, in addition to other behavioural assessment of anhedonia that have yet to be applied in the context of dopaminergic manipulation).^47^ The SPT does not measure reward learning or motivation for reward, but reflects impairments in reward processing such as those induced by exposure to stress: potentially because the animals either require higher concentrations of sucrose to differentiate from water or they find the difference between solutions less rewarding. The SPT is often administered after a period of fasting leading to a high motivation state. However, the duration of fasting is not standardised, and a recent systematic review found that more than half of studies fasted animals for more than 19 h.^46^ This approach might lead to a drive in sucrose preference for metabolic rather than hedonic reasons (i.e. ‘wanting’ not ‘liking’).^48^ Current clinical applications of the term anhedonia go beyond pleasure (e.g. the MADRS and other scales do not just ask about “experienced pleasure”). So, the mapping between animal and human data would be improved by considering a range of methods in animals—not just those that focus better on pleasure. Deficits in sucrose preference are also largely restricted to stress-induced models of depression with variations across different types of animals and different depression models. Twenty-one of the 27 included studies used a stress induced model relevant to depression, and we did not find any heterogeneity in the results of these compared with other (drug, surgical) models but may not have been powered to do so; others have reported such heterogeneity. Unfortunately, the test-retest reliability of SPT in the same animals was not reported, many studies used single-item measurements, only a few reported measures such as randomisation or blinding, and none of them was pre-registered, although study registration is rare in in vivo research. Efficacy was seen consistently across all circumstances of testing, for different drugs given at different times in different models of depression to different sexes of animals. Such consistency of effects across different manipulations is a-priori somewhat implausible; so it is possible that the circumstances of testing have not been examined across a sufficiently wide range to identify any limits to efficacy. Such consistency may also result from a failure to report null results, and we did find some evidence for publication bias.

Our study has some potential limitations. There is still debate in the scientific literature about the different dopamine or non-dopamine pathways, with different levels of pro-dopaminergic effect across various medications.^49^ These pathways are not isolated and can interact with each other, contributing to a complex interplay of brain functions.^50^ Understanding these pathways is likely to be crucial for understanding various neurological and psychiatric disorders and developing potential treatments. As reported in the protocol,^9^ in this review we included pharmacological treatments with a direct dopaminergic agonism or partial agonism mechanism of action at the central nervous system, using the Neuroscience based Nomenclature (https://nbn2r.com). For transparency, we reported in the data repository available on Open Science Framework the full list of drugs included in our search strategy (https://osf.io/2d967), however we acknowledge the possibility that we may have missed some relevant compounds. In this first iteration of the living systematic review, for human studies, we deliberately included only placebo-controlled randomised trials. Almost all data were about bupropion, which limits the generalisability of our findings. The next step is to expand to non-dopaminergic agents and to non-randomised studies, especially if they use innovative analytical approaches like Mendelian randomisation. Our researchers with lived experience emphasised the need for future research to account for the wide-ranging heterogeneity of symptomatology, individual differences (including ethnicity culture, and gender), and co-occurring conditions that patients within real-world clinical contexts experience. Embedding coproduction within research methodology (including the triangulation process) can assist teams in asking clinically relevant questions such as these, establishing a more comprehensive approach to knowledge creation.^51^ Measuring anhedonia is a challenge because there is no gold standard measurement. Anhedonia fluctuates over time, for example in the context of melancholic depression it mirrors the diurnal variation of mood, but it is also susceptible to change in terms of responsivity particularly in the early response to antidepressants, and these fluctuations are not captured by an aggregate measure. There are no data on how rating scales of anhedonia (e.g., MADRS Item 8 or SHAPS) map onto behavioural data or clinical assessment in depression. In our work we identified publications using outcomes related to motivation, pleasure or loss of interest as proxy for anhedonia. It is challenging to combine these outcomes because they are phenomenologically different (as regard to patient experience) and might relate to different biological systems. It is also possible that agents acting on appetite-regulating systems (like the hypothalamus and related pathways) may influence food consumption and preference independently of their effects on hedonic capacity (i.e., the ability to experience pleasure).^52^ There is a need for a refined conceptualization of anhedonia (also with more input from people with lived experience) and use of a data-driven approach to define key concepts such as motivation and pleasure.

In conclusion, this living systematic review of pro-dopaminergic interventions in human and animal studies found that pro-dopaminergic interventions reduce the symptoms of anhedonia with a small effect size and mostly related to bupropion, with further evidence from animal studies suggesting that this effect may occur via a direct as well as an indirect dopaminergic mechanism. However, these conclusions are limited by a lack of data available on reward and reinforcement tasks in human studies and the translational validity of the sucrose preference test. Moreover, data suggest that the manipulation of pro-dopaminergic pathways is neither necessary nor sufficient to improve anhedonia in depression models in rodents and in humans. Until additional studies using pro-dopaminergic drugs are evaluated in more clinically relevant animal tasks than the SPT, the link between human and animal studies will remain limiting the triangulation process.

Additional analysis of human studies suggested that pro-dopaminergic interventions were not as effective as other antidepressants, highlighting the complexity of studying anhedonia and its underlying mechanism. Further research is needed focusing on other pro-dopaminergic interventions and non-dopaminergic interventions for anhedonia (including glutamatergic treatments, anti-inflammatory therapies, psychedelics and the development of new molecules) and to search for other targets of pro-dopaminergic interventions than anhedonia. There is also the need to generate more data on reward and reinforcement tasks to allow a better understanding of human and animal data regarding the concept of anhedonia and the relationship between pro-dopaminergic interventions and improvement of anhedonia.

## Contributors

Edoardo G. Ostinelli: Literature search, data curation, formal analysis, methodology, software, visualisation, data interpretation, writing-review & editing.

Georgia Salanti: Formal analysis, methodology, supervision, validation, visualisation, data interpretation, writing-review & editing.

Malcolm Macleod: Literature search, formal analysis, methodology, supervision, data interpretation, writing-review & editing.

Virginia Chiocchia: Formal analysis, methodology, software, visualisation, data interpretation, writing-review & editing.

Katharine A. Smith: Data interpretation, writing-review & editing.

Argyris Stringaris: Data interpretation, writing-review & editing.

James Downs: Data interpretation, writing-review & editing.

Emma S J Robinson: Data interpretation, writing-review & editing.

Gin S. Malhi: Data interpretation, writing-review & editing.

Dominic M Dwyer: Data interpretation, writing-review & editing.

Astrid Chevance: Data interpretation, writing-review & editing.

Christoph Correll: Data interpretation, writing-review & editing.

Thomy Tonia: Data interpretation, writing-review & editing.

Emily Wheeler: Data interpretation, writing-review & editing.

Toshi A. Furukawa: Data interpretation, writing-review & editing.

Diego A. Pizzagalli: Data interpretation, writing-review & editing.

Michael Browning: Data interpretation, writing-review & editing.

Jennifer Potts: Literature search, writing-review & editing.

Andrea Cipriani: Conceptualisation, literature search, data curation, formal analysis, supervision, validation, visualisation, writing-original draft.

Edoardo G Ostinelli, Georgia Salanti, Malcolm Macleod, Virginia Chiocchia and Andrea Cipriani have accessed and verified the underlying data, including the original articles of both included and excluded studies, as well as the data extracted for the meta-analysis. All authors have read and approved the final version of the manuscript.

## Data sharing statement

This living systematic review is based on aggregate data either from published peer-reviewed manuscripts or from clinical trials register, and the data are publicly available. All the data and the code for meta-analysis can be accessed through the GALENOS website (galenos.org.uk) and the GALENOS data repository (https://galenos-data.aliveevidence.org/data_browser). The individual patient data network meta-analysis is part of an ongoing project (PETRUSHKA study) and the access to the data used for these analyses is restricted according to an agreement between the University of Oxford, Vivli and pharmaceutical companies.

## Declaration of interests

Edoardo G. Ostinelli received research and consultancy fees from Angelini Pharma. Gin Malhi has received personal payment for lecture from Abbvie and institutional grants from AstraZeneca and Janssen-Cilag. Emma Robinson has received funding for collaborative and contract research from pharmaceutical companies, Boehringer Ingelheim, Compass Pathways, Eli Lilly, IRLabs Therapeutics, Pfizer and SmallPharma and acted as a paid consultant for Compass Pathways and Pangea Botanicals. Christoph U. Correll has been a consultant or advisor to, or has received honoraria from, AbbVie, Alkermes, Allergan, Angelini, Aristo, Autobahn, Boehringer-Ingelheim, Bristol-Meyers Squibb, Cardio Diagnostics, Cerevel, CNX Therapeutics, Compass Pathways, Darnitsa, Delpor, Denovo, Draig, Eli Lilly, Eumentis Therapeutics, Gedeon Richter, GH Pharma, Hikma, Holmusk, IntraCellular Therapies, Jamjoom Pharma, Janssen, J&J, Karuna, LB Pharma, Lundbeck, MedInCell, MedLink, Merck, Mindpax, Mitsubishi Tanabe Pharma, Maplight, Mylan, Neumora Therapeutics, Neuraxpharm, Neurocrine, Neurelis, NeuShen, Newron, Noven, Novo Nordisk, Orion Pharma, Otsuka, PPD Biotech, Recordati, Relmada, Response Pharmaeutical, Reviva, Rovi, Saladax, Sanofi, Seqirus, Servier, Sumitomo Pharma America, Sunovion, Sun Pharma, Supernus, Tabuk, Takeda, Teva, Terran, Tolmar, Vertex, Viatris, and Xenon Pharmaceuticals; has provided expert testimony for Janssen, Lundbeck, Neurocrine, and Otsuka; served on a Data Safety Monitoring Board for Compass Pathways, IntraCellular Therapies, Relmada, Reviva, Rovi; has received grant support from Boehringer-Ingelheim, Janssen, and Takeda; has received royalties from UpToDate; and is a stock option holder of Cardio Diagnostics, Kuleon Biosciences, LB Pharma, MedLink Global, Mindpax, Quantic, and Terran. Toshi A Furukawa reports personal fees from Boehringer-Ingelheim, Daiichi Sankyo, D.T. Axis, Micron, Shionogi, SONY and UpToDate, and a grant from D.T. Axis and Shionogi, outside the submitted work; in addition, he has a patent 7448125 (Toshi Furukawa, Masaru Horikoshi, Takashi Katayama for applying machine learning in internet CBT) and a pending patent 2024–521973 (Shionogi, Kyoto University, a machine learning algorithm to predict depression worsening), and has licensed intellectual properties for Kokoro-app to D.T. Axis. Over the past three years, Diego Pizzagalli has received consulting fees from Arrowhead Therapeutics, Boehringer Ingelheim, Circular Genomics, Compass Pathways, Engrail Therapeutics, Neumora Therapeutics (formerly BlackThorn Therapeutics), Neurocrine Biosciences, Neuroscience Software, Sage Therapeutics, Sama Therapeutics, and Takeda; he has received honoraria from the American Psychological Association, Psychonomic Society and Springer (for editorial work) and from Alkermes; he has received research funding from the BIRD Foundation, Brain and Behavior Research Foundation, Dana Foundation, DARPA, Millennium Pharmaceuticals, NIMH and Wellcome Leap MCPsych; he has received stock options from Compass Pathways, Engrail Therapeutics, Neumora Therapeutics, and Neuroscience Software. Michael Browning has received consulting fees from Jansen Research, Engrail Therapeutics, Boehringer, P1vital Ltd, Alto Neuroscience, CHDR, and travel expenses from Lundbeck; he was previously employed by P1vital Lrd. Andrea Cipriani has received research, educational and consultancy fees from the Italian Network for Paediatric Trials, CARIPLO Foundation, Lundbeck, and Angelini Pharma outside the submitted work. No funding from these entities was used to support the current work, and all views expressed are solely those of the authors. Other authors report no conflicts of interest.
