# Supplementary material for: Pro-dopaminergic pharmacological interventions for anhedonia in depression: a living systematic review and network meta-analysis of human and animal studies
Source: eBioMedicine. 2025 Oct 29;121:105967. doi: 10.1016/j.ebiom.2025.105967 (PMC12790147; doi:10.1016/j.ebiom.2025.105967)
Supplement: Appendix [file mmc1.pdf]

Pro-dopaminergic pharmacological interventions for anhedonia in depression: a living systematic review and meta-analysis of human and non-human data

Appendix

Table of Contents

Table of Contents ... 2

Human studies ... 6

1. Methods ... 7

2. Results ... 9

2.1 Flow diagram ... 9

2.2 Description of included studies and reference list ... 10

2.3 Primary outcome: reduction in anhedonia scores at 8 weeks (from 4 to 12 weeks) ... 17

2.3.1 Pairwise meta-analysis ... 17

2.3.2 Risk of bias ... 17

2.3.3 Reporting bias ... 18

2.3.4 Meta-regression analyses ... 18

2.3.5 Post-hoc analyses ... 18

2.4 Secondary outcome: Reduction in mean anxiety score at 8 weeks (from 4 to 12 weeks) ... 20

2.4.1 Pairwise meta-analysis ... 20

2.4.2 Risk of Bias ... 21

2.4.3 Meta-regression analyses ... 22

2.5 Secondary outcome: Dropouts due to any reason ... 23

2.5.1 Pairwise meta-analysis ... 23

2.5.2 Risk of bias ... 24

2.5.3 Meta-regression analyses ... 25

2.6 Secondary outcome: dropouts due to side effects ... 26

2.6.1 Pairwise meta-analysis ... 26

2.6.2 Risk of bias ... 27

2.6.3 Meta-regression analyses ... 28

2.7 Secondary outcome: nausea ... 29

2.7.1 Pairwise meta-analysis ... 29

2.7.2 Risk of bias ... 30

2.7.3 Meta-regression analyses ... 31

2.8 Secondary outcome: headache ... 32

2.8.1 Pairwise meta-analysis ... 32

2.8.2 Risk of bias ... 33

2.8.3 Meta-regression analyses ... 34

2.9 Secondary outcome: insomnia **35**
2.9.1 Pairwise meta-analysis 35
2.9.2 Risk of bias 36
2.9.3 Meta-regression analyses 37
2.10 Secondary outcome: constipation **38**
2.10.1 Pairwise meta-analysis 38
2.10.2 Risk of bias 39
2.10.3 Meta-regression analyses 40
2.11 Secondary outcome: dizziness **41**
2.12.1 Pairwise meta-analysis 41
2.11.2 Risk of bias 42
2.11.3 Meta-regression analyses 43
2.12 Secondary outcome: dry mouth **44**
2.12.1 Pairwise meta-analysis 44
2.12.2 Risk of bias 45
2.12.3 Meta-regression analyses 46
2.13 Secondary outcome: vomiting **47**
2.13.1 Pairwise meta-analysis 47
2.13.2 Risk of bias 48
2.13.3 Meta-regression analyses 48
2.14 Summary of evidence tables **49**
3. Abbreviations ***58***
4. Software Used ***59***
5. References ***59***
6 Table of included studies ***60***
7. Risk of Bias - Missing Evidence Assessment ***86***
7.1 ROB-ME Matrix Step 1 .... **86**
7.2 ROB-ME Matrix Step 2 .... **89**
7.3 ROB-ME Step 3 & 4 .... **95**

Non-human animal studies ***100***
1. Flow of study selection and descriptives ***101***
1.1 Description of experiment types and methodological approach **110**
2 Dopaminergic agent v Control ***111***
2.1 Outcome 1: Sucrose preference **113**

2.1.1 Risk of bias 113
2.1.2 Reporting completeness.. 114
2.1.3 Meta-analysis 114
2.1.4 Subgroup analyses and meta-regressions... 116
2.1.5 Sensitivity Analyses.. 130
2.1.6 Reporting bias/small-study effects 132

2.2 Outcome 2: Dopamine concentration 134
2.2.1 Risk of bias 134
2.2.2 Reporting completeness.. 135
2.2.3 Meta-analysis 136

2.3 Outcome 3: DOPAC concentration 136

2.4 Outcome 4: Dopamine / DOPAC ratio 136

3 Effects of model induction 137

3.1 Outcome 1: Sucrose preference.. 138
3.1.1 Risk of bias 138
3.1.2 Reporting completeness.. 139
3.1.3 Meta-analysis 140
3.1.4 Subgroup analyses and meta-regressions... 142
3.1.5 Sensitivity Analyses.. 148
3.1.6 Reporting bias/small-study effects 149

3.2 Outcome 2: Dopamine concentrations... 150
3.2.1 Risk of bias 150
3.2.2 Reporting completeness.. 151
3.2.3 Meta-analysis 152

3.3 Outcome 3: DOPAC concentration 152

3.4 Outcome 4: Dopamine / DOPAC ratio 152

3.5 Outcome 5: Dopamine receptor biology 152

4. Observed relationships between different outcomes measures in the same cohorts of animals.. 153

4.1 Relationship between change in Sucrose preference test and change in measured dopamine concentrations.. 153

4.2 Relationship between change in Sucrose preference test and change in measured dopamine / DOPAC ratio 154

4.3 Relationship between change in dopamine concentrations and change in DOPAC concentrations 155

4.4 Relationship between change in dopamine concentrations and change in dopamine/DOPAC ratio 156

5. Attrition bias and adverse effects of treatment 157

6. Summary of the evidence 158

6.1 Dopaminergic agents versus control 158

6.2 Effect of inducing model, without treatment 159

Evaluation of indirectness of evidence (based on criteria in document "Assessing the certainty of evidence in animal studies") for the studies included in the review 161

Evaluation of the concordance between different outcome measures 165

7. Software used 165

References........166

Human studies

1. Methods

In this first iteration of the living systematic review we searched for randomised controlled trials that compared pro-dopaminergic interventions to placebo in adults with unipolar depression (i.e. above-threshold symptoms on any standardised measure, or a clinical diagnosis based on any operationalised criteria).

Eight databases were searched from inception up to the 9th of November, 2023 (see protocol for full search strings). Database search results were imported into EPPI-Reviewer and duplicates were removed prior to screening. All steps related to record screening and data extraction were completed in EPPI-Reviewer.

Titles and abstracts of the identified records were screened by at least two reviewers (CF, MC, JK, JP). We retrieved the full-texts and any supporting documents for all records that were not excluded at the title and abstract screening stage. The full-text screening was conducted by at least two reviewers (CF, JK, JP, AK, EB). Conflicts at title and abstract, and full-text screening, were resolved through discussion between the two reviewers and involvement of a third reviewer (AC, EGO).

We focused on the following outcomes: - anhedonia symptom severity: using anhedonia-specific scales, anhedonia-specific sub-scales, or individual items focusing on anhedonia (observer-rated or self-rated). Continuous, primary outcome. - anxiety symptom severity: as per observer or self-reported standardised scales. Continuous, secondary outcome. - acceptability: proportion of participants dropping out for any reason. Binary, secondary outcome. - tolerability: the proportion of participants dropping out due to an adverse event. Binary, secondary outcome. - safety: the proportion of participants reporting specific adverse events (nausea, headache, insomnia, constipation, dizziness, dry mouth, vomiting). Binary, secondary outcome.

Additional information on the full study eligibility criteria can be found in the pre-published protocol.

For anhedonia and anxiety symptom severity, we extracted outcome data reported at 8 weeks post-treatment or manipulation. If the information at 8 weeks was not available, we considered eligible data ranging between 4 and 12 weeks (with preference to the time point closest to 8 weeks and, if equidistant, the longer outcome). For acceptability, tolerability, safety and safety (specific adverse events), we extracted outcome data reported at the end of the studies.

When extracting continuous outcomes we extracted mean and standard deviation to two decimal places. Where standard error was reported, we converted the value to standard deviation. Baseline and endpoint values were extracted. These were preferred to change in score and endpoint, in which case the missing value was calculated by adding or subtracting the change in score from the time point given.

When extracting dichotomous outcomes we extracted natural numbers and where only percentages of participant groups were reported, a value was calculated and rounded up to the nearest natural number. Adverse events were extracted using the exact wording used to report them in the included studies.

Relevant data was extracted using EPPI-Reviewer by at least two reviewers (CF, CA, EB, JK). EPPI-Reviewer was used to screen records, extract data, and assess risk of bias.

We assessed risk of bias with the RoB2 tool (Higgins et al. 2019). All outcomes for all included studies were assessed by at least two reviewers (JK, CF, CA, AH) and conflicts were resolved by discussion between reviewers. To evaluate biases due to missing evidence, the ROB-ME tool (Page et al. 2023) was used with the same double screening and conflict resolution process as described above.

Effect sizes were calculated as standardised mean differences (SMDs) for continuous outcomes (anhedonia and anxiety symptom severity) and odds ratios (ORs) for dichotomous outcomes (acceptability, tolerability, and specific-adverse events). We calculated the 95% confidence interval (CI) around the pooled effect size for each meta-analysis.

Meta-analyses were conducted using a random effects model with the inverse variance method, using the restricted maximum-likelihood estimator for τ^2 and the Hartung-Knapp correction method to adjust 95% confidence intervals, if there are at least five studies. Prediction intervals of the overall pooled effect were calculated to convey the amount of heterogeneity.

In order to better contextualise pro-dopaminergic interventions within wider literature on the effects of antidepressants on anhedonia, we aggregated individual participant data (IPD) on the MADRS “inability to feel” item from 34 randomised controlled trials (14054 participants) on antidepressants in people with depression that we had access to. We utilised this post-hoc analysis to add further evidence on the impact of non-dopaminergic interventions on anhedonia in depression and in doing so, better understand the evidence we analysed on dopamine-specific drugs. We performed the following analyses to estimate the performance of bupropion versus placebo at the primary outcome (i.e. reduction in anhedonia scores):

- aggregated IPD, all antidepressants and placebo, random effects network meta-analysis.
- aggregated IPD, bupropion versus placebo, random effects pairwise meta-analysis.
- combination of aggregated IPD and early studies identified in this living systematic review for which IPD were not available, random effects pairwise meta-analysis.

Meta-regressions were planned for the following variables: mean age of participants, mean anhedonia baseline score, mean anxiety baseline score, sex (proportion of female participants), and planned treatment duration. Meta-regressions were only conducted for outcomes where data was available from 10 or more studies.

Summary of evidence tables were constructed for all outcomes including a summary of the meta-analytic result, biases within-study, across-study, and due to indirectness.

Please refer to the protocol and the extended data for more details.

A list of abbreviations can be found towards the end of the document.

2. Results

2.1 Flow diagram

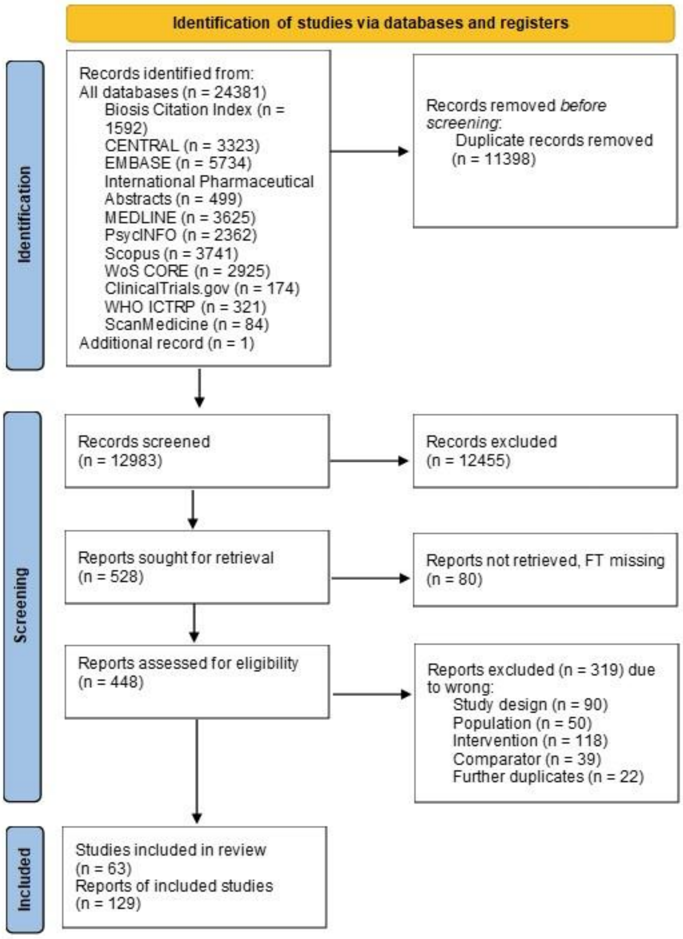

Figure 1. PRISMA 2020 flow diagram. `

2.2 Description of included studies and reference list

We identified 63 eligible studies. The characteristics of the identified studies can be found in **Table 1**. Data from studies contributed with at least one outcome with quantitative data (total of 10532 participants), which included adults from multiple countries. The mean age of participants was 42.2 years (range 15 to 72 years), with a mean proportion of 0.58 female participants (range 0 to 0.86). Included studies allocated the participants to treatment lasting between 4 to 12.9 weeks (median, 6 weeks).

1. Agosti V, Stewart JW, Quitkin FM. Life satisfaction and psychosocial functioning in chronic depression: effect of acute treatment with antidepressants. J Affect Disord. 1991 Sep;23(1):35-41. doi: 10.1016/0165-0327(91)90033-o. PMID: 1774421.
2. Amsterdam JD, Dunner DL, Fabre LF, Kiev A, Rush AJ, Goodman LI. Double-blind, placebo-controlled, fixed dose trial of minaprine in patients with major depression. Pharmacopsychiatry. 1989 Jul;22(4):137-43. doi: 10.1055/s-2007-1014596. PMID: 2668978.
3. Amsterdam JD. A double-blind, placebo-controlled trial of the safety and efficacy of selegiline transdermal system without dietary restrictions in patients with major depressive disorder. J Clin Psychiatry. 2003 Feb;64(2):208-14. doi: 10.4088/jcp.v64n0216. PMID: 12633131.
4. Bakish D, Bradwejn J, Nair N, McClure J, Remick R, Bulger L. A comparison of moclobemide, amitriptyline and placebo in depression: a Canadian multicentre study. Psychopharmacology (Berl). 1992;106 Suppl:S98-101. doi: 10.1007/BF02246248. Erratum in: Psychopharmacology (Berl) 1993;111(3):389-90. PMID: 1546154.
5. Bellak L, Rosenberg S. Effects of anti-depressant drugs on psychodynamics. Psychosomatics. 1966 Mar-Apr;7(2):106-14. doi: 10.1016/S0033-3182(66)72178-1. PMID: 5325692.
6. Benes H, Mattern W, Peglau I, Dreykluft T, Bergmann L, Hansen C, Kohnen R, Banik N, Schoen SW, Hornyak M. Ropinirole improves depressive symptoms and restless legs syndrome severity in RLS patients: a multicentre, randomized, placebo-controlled study. J Neurol. 2011 Jun;258(6):1046-54. doi: 10.1007/s00415-010-5879-7. Epub 2010 Dec 28. PMID: 21188406.
7. Bodkin JA, Amsterdam JD. Transdermal selegiline in major depression: a double-blind, placebo-controlled, parallel-group study in outpatients. Am J Psychiatry. 2002 Nov;159(11):1869-75. doi: 10.1176/appi.ajp.159.11.1869. PMID: 12411221.
8. Botte L, Evrard JL, Gilles C, Stenier P, Wolfrum C. Controlled comparison of RO 11-1163 (moclobemide) and placebo in the treatment of depression. Acta Psychiatr Belg. 1992;92(6):355-69. PMID: 1345409.

9. Bymaster F, Skolnick P, Huang NY, Bradshaw M, McKinney A, Manthius J, Fava M, Tran P. Efficacy and safety of EB-1010, a triple reuptake inhibitor, in the treatment of patients with major depressive disorder. Biol Psychiatry. 2011; 69:37S.
10. Casacchia M, Carolei A, Barba C, Frontoni M, Rossi A, Meco G, Zylberman MR. A placebo-controlled study of the antidepressant activity of moclobemide, a new MAO-A inhibitor. Pharmacopsychiatry. 1984 Jul;17(4):122-5. doi: 10.1055/s-2007-1017421. PMID: 6382361.
11. Chouinard G, Saxena BM, Nair NP, Kutcher SP, Bakish D, Bradwejn J, Kennedy SH, Sharma V, Remick RA, Kukha-Mohamad SA, et al. Brofaromine in depression: a Canadian multicenter placebo trial and a review of standard drug comparative studies. Clin Neuropharmacol. 1993;16 Suppl 2:S51-4. PMID: 8313397.
12. Clayton AH, Croft HA, Horrigan JP, Wightman DS, Krishen A, Richard NE, Modell JG. Bupropion extended release compared with escitalopram: effects on sexual functioning and antidepressant efficacy in 2 randomized, double-blind, placebo-controlled studies. J Clin Psychiatry. 2006 May;67(5):736-46. doi: 10.4088/jcp.v67n0507. PMID: 16841623.
13. Coleman CC, Cunningham LA, Foster VJ, Batey SR, Donahue RM, Houser TL, Ascher JA. Sexual dysfunction associated with the treatment of depression: a placebo-controlled comparison of bupropion sustained release and sertraline treatment. Ann Clin Psychiatry. 1999 Dec;11(4):205-15. doi: 10.1023/a:1022309428886. PMID: 10596735.
14. Coleman CC, King BR, Bolden-Watson C, Book MJ, Segraves RT, Richard N, Ascher J, Batey S, Jamerson B, Metz A. A placebo-controlled comparison of the effects on sexual functioning of bupropion sustained release and fluoxetine. Clin Ther. 2001 Jul;23(7):1040-58. doi: 10.1016/s0149-2918(01)80090-4. PMID: 11519769.
15. Corrigan MH, Denahan AQ, Wright CE, Ragual RJ, Evans DL. Comparison of pramipexole, fluoxetine, and placebo in patients with major depression. Depress Anxiety. 2000;11(2):58-65. doi: 10.1002/(sici)1520-6394(2000)11:2<58::aid-da2>3.0.co;2-h. PMID: 10812530.
16. Croft H, Settle E Jr, Houser T, Batey SR, Donahue RM, Ascher JA. A placebo-controlled comparison of the antidepressant efficacy and effects on sexual functioning of sustained-release bupropion and sertraline. Clin Ther. 1999 Apr;21(4):643-58. doi: 10.1016/S0149-2918(00)88317-4. PMID: 10363731.
17. Davidson JR, Giller EL, Zisook S, Overall JE. An efficacy study of isocarboxazid and placebo in depression, and its relationship to depressive nosology. Arch Gen Psychiatry. 1988 Feb;45(2):120-7. doi: 10.1001/archpsyc.1988.01800260024003. PMID: 3276281.
18. DelBello MP, Hochadel TJ, Portland KB, Azzaro AJ, Katic A, Khan A, Emslie G. A double-blind, placebo-controlled study of selegiline transdermal system in depressed adolescents. J Child Adolesc Psychopharmacol. 2014 Aug;24(6):311-7.

doi: 10.1089/cap.2013.0138. Epub 2014 Jun 23. PMID: 24955812; PMCID: PMC4137354.

19. Feiger AD, Rickels K, Rynn MA, Zimbroff DL, Robinson DS. Selegiline transdermal system for the treatment of major depressive disorder: an 8-week, double-blind, placebo-controlled, flexible-dose titration trial. J Clin Psychiatry. 2006 Sep;67(9):1354-61. doi: 10.4088/jcp.v67n0905. PMID: 17017821.
20. Feighner JP, Meredith CH, Stern WC, Hendrickson G, Miller LL. A double-blind study of bupropion and placebo in depression. Am J Psychiatry. 1984 Apr;141(4):525-9. doi: 10.1176/ajp.141.4.525. PMID: 6422779.
21. Georgotas A, McCue RE, Hapworth W, Friedman E, Kim OM, Welkowitz J, Chang I, Cooper TB. Comparative efficacy and safety of MAOIs versus TCAs in treating depression in the elderly. Biol Psychiatry. 1986 Oct;21(12):1155-66. doi: 10.1016/0006-3223(86)90222-2. PMID: 3756264.
22. Giller E, Bialos D, Riddle M, Sholomskas A, Harkness L. Monoamine oxidase inhibitor-responsive depression. Psychiatry Res. 1982 Feb;6(1):41-8. doi: 10.1016/0165-1781(82)90036-1. PMID: 7036196.
23. GlaxoSmithKline. A multicenter dose-response evaluation of the safety and efficacy of bupropion HCI sustained-release versus placebo in depressed outpatients. GSK clinical study register [www.gsk-clinicalstudyregister.com]. 1985.
24. GlaxoSmithKline. Multicenter Evaluation of the Efficacy and Safety of Bupropion vs. Placebo in Depressed Inpatients. GSK clinical study register [www.gsk-clinicalstudyregister.com]. 1993.
25. GlaxoSmithKline. A multicenter evaluation of the safety and efficacy of two flexible doses of WELLBUTRIN sustained-release versus placebo in depressed outpatients. GSK clinical study register [www.gsk-clinicalstudyregister.com]. 1994.
26. GlaxoSmithKline. Phase II double-blind evaluation of safety and efficacy of two dose ranges of bupropion vs. placebo in depressed outpatients. GSK clinical study register [www.gsk-clinicalstudyregister.com]. 1980.
27. Han DH, Renshaw PF. Bupropion in the treatment of problematic online game play in patients with major depressive disorder. J Psychopharmacol. 2012 May;26(5):689-96. doi: 10.1177/0269881111400647. Epub 2011 Mar 29. PMID: 21447539; PMCID: PMC4638175.
28. Hewett K, Chrzanowski W, Schmitz M, Savela A, Milanova V, Gee M, Krishen A, Millen L, Leary MO, Modell J. Eight-week, placebo-controlled, double-blind comparison of the antidepressant efficacy and tolerability of bupropion XR and venlafaxine XR. J Psychopharmacol. 2009 Jul;23(5):531-8. doi: 10.1177/0269881108089602. Epub 2008 Jul 17. PMID: 18635695.
29. Hewett K, Gee MD, Krishen A, Wunderlich HP, Le Clus A, Evoniuk G, Modell JG. Double-blind, placebo-controlled comparison of the antidepressant efficacy and tolerability of bupropion XR and venlafaxine XR. J Psychopharmacol. 2010a

Aug;24(8):1209-16. doi: 10.1177/0269881109106953. Epub 2009 Nov 25. PMID: 19939870.

30. Hewett K, Chrzanowski W, Jokinen R, Felgentreff R, Shrivastava RK, Gee MD, Wightman DS, O'Leary MC, Millen LS, Leon MC, Briggs MA, Krishen A, Modell JG. Double-blind, placebo-controlled evaluation of extended-release bupropion in elderly patients with major depressive disorder. J Psychopharmacol. 2010b Apr;24(4):521-9. doi: 10.1177/0269881108100254. Epub 2009 Jan 22. PMID: 19164492.
31. Iosifescu DV, Jones A, O'Gorman C, Streicher C, Feliz S, Fava M, Tabuteau H. Efficacy and Safety of AXS-05 (Dextromethorphan-Bupropion) in Patients With Major Depressive Disorder: A Phase 3 Randomized Clinical Trial (GEMINI). J Clin Psychiatry. 2022 May 30;83(4):21m14345. doi: 10.4088/JCP.21m14345. PMID: 35649167.
32. Jarrett RB, Schaffer M, McIntire D, Witt-Browder A, Kraft D, Risser RC. Treatment of atypical depression with cognitive therapy or phenelzine: a double-blind, placebo-controlled trial. Arch Gen Psychiatry. 1999 May;56(5):431-7. doi: 10.1001/archpsyc.56.5.431. PMID: 10232298; PMCID: PMC1475805.
33. Jefferson JW, Rush AJ, Nelson JC, VanMeter SA, Krishen A, Hampton KD, Wightman DS, Modell JG. Extended-release bupropion for patients with major depressive disorder presenting with symptoms of reduced energy, pleasure, and interest: findings from a randomized, double-blind, placebo-controlled study. J Clin Psychiatry. 2006 Jun;67(6):865-73. doi: 10.4088/jcp.v67n0602. PMID: 16848645.
34. Koshino Y, Bahk WM, Sakai H, Kobayashi T. The efficacy and safety of bupropion sustained-release formulation for the treatment of major depressive disorder: a multi-center, randomized, double-blind, placebo-controlled study in Asian patients. Neuropsychiatr Dis Treat. 2013;9:1273-80. doi: 10.2147/NDT.S48158. Epub 2013 Aug 28. PMID: 24039429; PMCID: PMC3770623.
35. Kusalic M, Engelsmann F, Bradwejn J. Thyroid functioning during treatment for depression. J Psychiatry Neurosci. 1993 Nov;18(5):260-3. PMID: 8297924; PMCID: PMC1188546.
36. Larsen JK, Holm P, Høyer E, Mejlhede A, Mikkelsen PL, Olesen A, Schaumburg E. Moclobemide and clomipramine in reactive depression. A placebo-controlled randomized clinical trial. Acta Psychiatr Scand. 1989 Jun;79(6):530-6. doi: 10.1111/j.1600-0447.1989.tb10299.x. PMID: 2669441.
37. Learned S, Graff O, Roychowdhury S, Moate R, Krishnan KR, Archer G, Modell JG, Alexander R, Zamuner S, Lavergne A, Evoniuk G, Ratti E. Efficacy, safety, and tolerability of a triple reuptake inhibitor GSK372475 in the treatment of patients with major depressive disorder: two randomized, placebo- and active-controlled clinical trials. J Psychopharmacol. 2012 May;26(5):653-62. doi: 10.1177/0269881111424931. Epub 2011 Nov 2. PMID: 22048884.
38. Liebowitz MR, Quitkin FM, Stewart JW, McGrath PJ, Harrison W, Rabkin J, Tricamo E, Markowitz JS, Klein DF. Phenelzine v imipramine in atypical depression. A preliminary

report. Arch Gen Psychiatry. 1984 Jul;41(7):669-77. doi: 10.1001/archpsyc.1984.01790180039005. PMID: 6375621.
39. Mann JJ, Aarons SF, Wilner PJ, Keilp JG, Sweeney JA, Pearlstein T, Frances AJ, Kocsis JH, Brown RP. A controlled study of the antidepressant efficacy and side effects of (-)-deprenyl. A selective monoamine oxidase inhibitor. Arch Gen Psychiatry. 1989 Jan;46(1):45-50. doi: 10.1001/archpsyc.1989.01810010047007. PMID: 2491941.
40. Nair NP, Amin M, Holm P, Katona C, Klitgaard N, Ng Ying Kin NM, Kragh-Sørensen P, Kühn H, Leek CA, Stage KB. Moclobemide and nortriptyline in elderly depressed patients. A randomized, multicentre trial against placebo. J Affect Disord. 1995 Jan 11;33(1):1-9. doi: 10.1016/0165-0327(94)00047-d. PMID: 7714303.
41. Ose E, Holm P. Moclobemide and placebo in mild major depression: a double-blind randomized trial. Psychopharmacology (Berl). 1992;106 Suppl:S114-5. doi: 10.1007/BF02246251. PMID: 1546122.
42. Parnetti L, Sommacal S, Labate AMM, Senin U. Multicentre Controlled Randomised Double-Blind Placebo Study of Minaprine in Elderly Patients Suffering from Prolonged Depressive Reaction. Drug Invest. 1993;6,181-188. doi: 10.1007/BF03259242.
43. Quitkin FM, McGrath PJ, Stewart JW, Harrison W, Tricamo E, Wager SG, Ocepek-Welikson K, Nunes E, Rabkin JG, Klein DF. Atypical depression, panic attacks, and response to imipramine and phenelzine. A replication. Arch Gen Psychiatry. 1990 Oct;47(10):935-41. doi: 10.1001/archpsyc.1990.01810220051006. PMID: 2222132.
44. Raft D, Davidson J, Wasik J, Mattox A. Relationship between response to phenelzine and MAO inhibition in a clinical trial of phenelzine, amitriptyline and placebo. Neuropsychobiology. 1981;7(3):122-6. doi: 10.1159/000117841. PMID: 7231652.
45. Rampello L, Nicoletti G, Raffaele R. Dopaminergic hypothesis for retarded depression: a symptom profile for predicting therapeutical responses. Acta Psychiatr Scand. 1991 Dec;84(6):552-4. doi: 10.1111/j.1600-0447.1991.tb03193.x. PMID: 1792929.
46. Raskin A. Adverse reactions to phenelzine: results of a nine-hospital depression study. J Clin Pharmacol New Drugs. 1972 Jan;12(1):22-5. doi: 10.1002/j.1552-4604.1972.tb00031.x. PMID: 4550222.
47. Ravaris CL, Nies A, Robinson DS, Ives JO, Lamborn KR, Korson L. A multiple-dose, controlled study of phenelzine in depression-anxiety states. Arch Gen Psychiatry. 1976 Mar;33(3):347-50. doi: 10.1001/archpsyc.1976.01770030057008. PMID: 769725.
48. Reimherr FW, Cunningham LA, Batey SR, Johnston JA, Ascher JA. A multicenter evaluation of the efficacy and safety of 150 and 300 mg/d sustained-release bupropion tablets versus placebo in depressed outpatients. Clin Ther. 1998 May-Jun;20(3):505-16. doi: 10.1016/s0149-2918(98)80060-x. PMID: 9663366.

49. Rickels K, Gordon PE, Gansman DH, Weise CC, Pereira-Ogan JA, Hesbacher PT. Pemoline and methylphenidate in midly depressed outpatients. Clin Pharmacol Ther. 1970 Sep-Oct;11(5):698-710. doi: 10.1002/cpt1970115698. PMID: 5455633.
50. Riesenberg R, Rosenthal J, Moldauer L, Peterson C. Results of a Proof-of-Concept, Dose-Finding, Double-blind, Placebo-Controlled Study of Serdaxin in Subjects with Major Depressive Disorder. Poster Session II. Neuropsychopharmacol. 2010;35(S1):S214-S215 (2010). doi: 10.1038/npp.2010.217.
51. Robin AA, Wiseberg S. A controlled trial of methyl phenidate (ritalin) in the treatment of depressive states. J Neurol Neurosurg Psychiatry. 1958 Feb;21(1):55-7. doi: 10.1136/jnnp.21.1.55. PMID: 13514499; PMCID: PMC497294.
52. Rowan P, Paykel ES, Parker PR, West E. Comparative effects of phenelzine and amitriptyline: a placebo controlled trial. Neuropharmacology. 1980 Dec;19(12):1223-5. doi: 10.1016/0028-3908(80)90211-7. PMID: 7003429.
53. Tomarken AJ, Dichter GS, Freid C, Addington S, Shelton RC. Assessing the effects of bupropion SR on mood dimensions of depression. J Affect Disord. 2004 Mar;78(3):235-41. doi: 10.1016/S0165-0327(02)00306-3. PMID: 15013248.
54. Ucha Udabe R, Márquez CA, Traballi CA, Portes N. Double-blind comparison of moclobemide, imipramine and placebo in depressive patients. Acta Psychiatr Scand Suppl. 1990;360:54-6. doi: 10.1111/j.1600-0447.1990.tb05330.x. PMID: 2123367.
55. UK Moclobemide Study Group. A multicentre comparative trial of moclobemide, imipramine and placebo in major depressive disorder. UK Moclobemide Study Group. Int Clin Psychopharmacol. 1994 Summer;9(2):109-13. doi: 10.1097/00004850-199400920-00007. PMID: 8056992.
56. Versiani M, Oggero U, Alterwain P, Capponi R, Dajas F, Heinze-Martin G, Marquez CA, Poleo MA, Rivero-Almanzor LE, Rossel L, et al. A double-blind comparative trial of moclobemide v. imipramine and placebo in major depressive episodes. Br J Psychiatry Suppl. 1989 Oct;(6):72-7. PMID: 2695129.
57. Versiani M, Nardi AE, Mundim FD, Alves A, Schmid-Burgk W. Moclobemide, imipramine and placebo in the treatment of major depression. Acta Psychiatr Scand Suppl. 1990;360:57-8. doi: 10.1111/j.1600-0447.1990.tb05331.x. PMID: 2248073.
58. Versiani M, Amrein R, Stabl M. Moclobemide and imipramine in chronic depression (dysthymia): an international double-blind, placebo-controlled trial. International Collaborative Study Group. Int Clin Psychopharmacol. 1997 Jul;12(4):183-93. doi: 10.1097/00004850-199707000-00001. PMID: 9347378.
59. White K, Razani J, Cadow B, Gelfand R, Palmer R, Simpson G, Sloane RB. Tranylcypromine vs nortriptyline vs placebo in depressed outpatients: a controlled trial. Psychopharmacology (Berl). 1984;82(3):258-62. doi: 10.1007/BF00427786. PMID: 6425910.
60. Zarate CA Jr, Singh JB, Quiroz JA, De Jesus G, Denicoff KK, Luckenbaugh DA, Manji HK, Charney DS. A double-blind, placebo-controlled study of memantine in the

treatment of major depression. Am J Psychiatry. 2006 Jan;163(1):153-5. doi: 10.1176/appi.ajp.163.1.153. PMID: 16390905.
61. Zisook S, Braff DL, Click MA. Monoamine oxidase inhibitors in the treatment of atypical depression. J Clin Psychopharmacol. 1985 Jun;5(3):131-7. PMID: 3889078.

2.3 Primary outcome: reduction in anhedonia scores at 8 weeks (from 4 to 12 weeks)

2.3.1 Pairwise meta-analysis

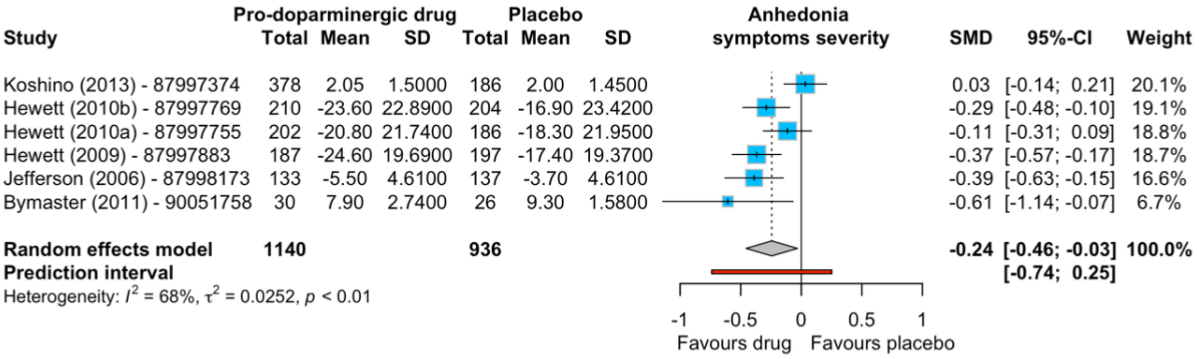

Figure 2. Forest plot for symptoms of anhedonia (primary outcome) comparing pro-dopaminergic interventions vs placebo for individuals with anhedonia at 4-12 weeks (primary timepoint). SMD: standardised mean difference, 95% CI: 95% confidence intervals, SD: standard deviation.

The effect of pro-dopaminergic drugs versus placebo showed an effect favouring pro-dopaminergic interventions with a SMD of -0.244 (95% CI from -0.456 to -0.031). There is some heterogeneity as shown by the 95% prediction interval from -0.74 to 0.252.

2.3.2 Risk of bias

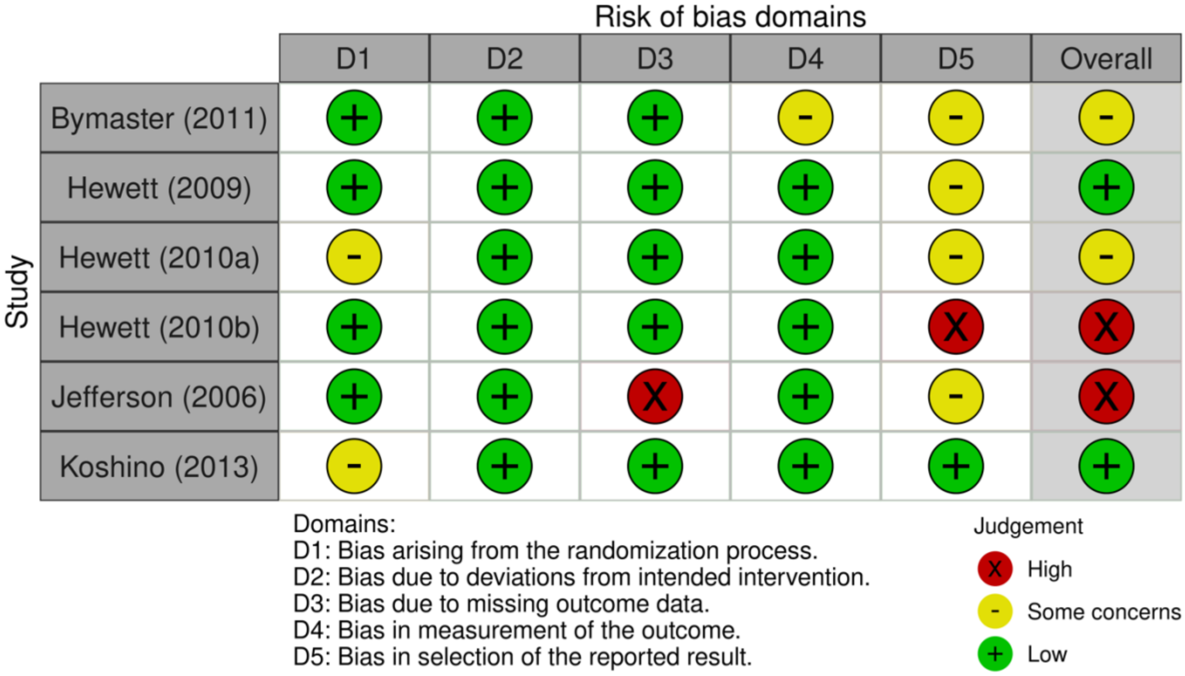

Figure 3. Risk of bias assessment.

Evidence for the efficacy of pro-dopaminergic interventions vs placebo were rated as having a range in their overall risk of bias. Two studies (33%) were assessed as having ‘high’ risk of bias due to having ‘high’ risk of bias in the missing outcome data domain and selection of the reported results domain. Two studies (33%) had an overall ‘moderate’ risk of bias rating as they had ‘some concerns’ in two domain ratings. The remaining two studies were rated as having a ‘low’ overall risk of bias.

2.3.3 Reporting bias

The extent to which the result was affected by reporting biases was rated as low as per the RoB-ME assessment (Page et al. 2023). This was as the potential for missing studies across the review was judged to be low. In addition, none of the included studies were deemed to have generated an eligible result that was not reported, and no studies were judged to be unclear as to whether they generated an eligible result that was not reported. We made this decision based on the results matrix we generated in step 2 of the RoB-ME tool (Page et al. 2023).

2.3.4 Meta-regression analyses

We did not perform any meta-regressions as the total number of studies was below 10.

2.3.5 Post-hoc analyses

We had access to individual participant data (IPD) of randomised controlled trials on depression from a NIHR-funded project. We performed a series of post-hoc analyses on the MADRS “inability to feel” item (aggregated IPD from 34 studies, 14054 participants).

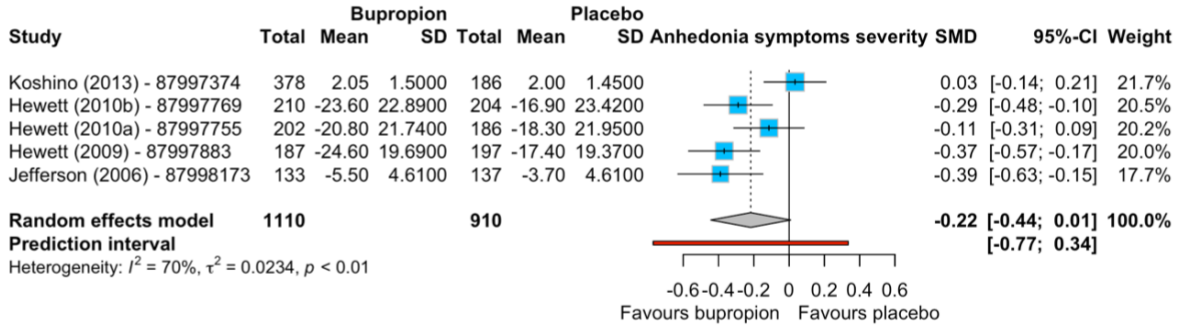

We conducted network meta-analysis. **Figure 4** shows the results of each active drug versus placebo. SMD: standardised mean difference, 95% CI: 95% confidence intervals, SD: standard deviation.

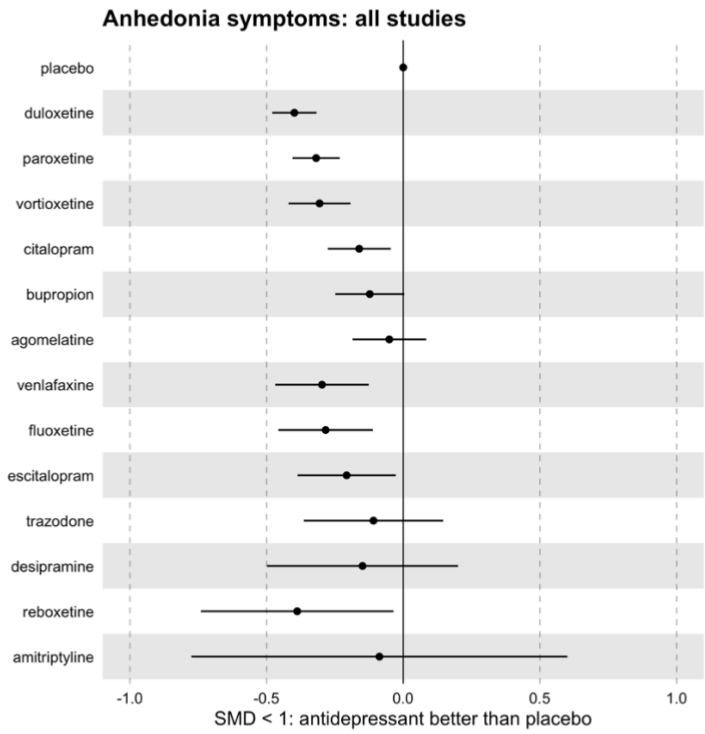

SMD	95% LCI	95% UCI
.	.	.
-0.40	-0.48	-0.32
-0.32	-0.41	-0.23
-0.31	-0.42	-0.19
-0.16	-0.28	-0.05
-0.12	-0.25	0.00
-0.05	-0.19	0.08
-0.30	-0.47	-0.13
-0.28	-0.46	-0.11
-0.21	-0.39	-0.03
-0.11	-0.36	0.15
-0.15	-0.50	0.20
-0.39	-0.74	-0.04
-0.09	-0.78	0.60

Figure 5. Summary SMD for the ‘inability to feel’ item on the MADRS scale, from indirect and direct evidence (network meta-analysis) of antidepressant versus placebo. Buproprion is the only pro-dopaminergic drug shown.

The mean effect of bupropion (pro-dopaminergic intervention) versus placebo was -0.12 (SMD, 95% CI from -0.25 to 0.00; 34 studies, 14054 participants). In comparison, we found effects across non-dopaminergic interventions to range from -0.09 (SMD, 95% CI from -0.19 to 0.08) in agomelatine to -0.50 (SMD, 95% CI from -0.48 to -0.32) in duloxetine. The range of effects found for bupropion verses non-dopaminergic interventions was 0.28 (SMD, 95%CI from -0.15 to 0.41) to -0.07 (SMD, 95%CI from -0.23 to 0.09).

2.4 Secondary outcome: Reduction in mean anxiety score at 8 weeks (from 4 to 12 weeks)

2.4.1 Pairwise meta-analysis

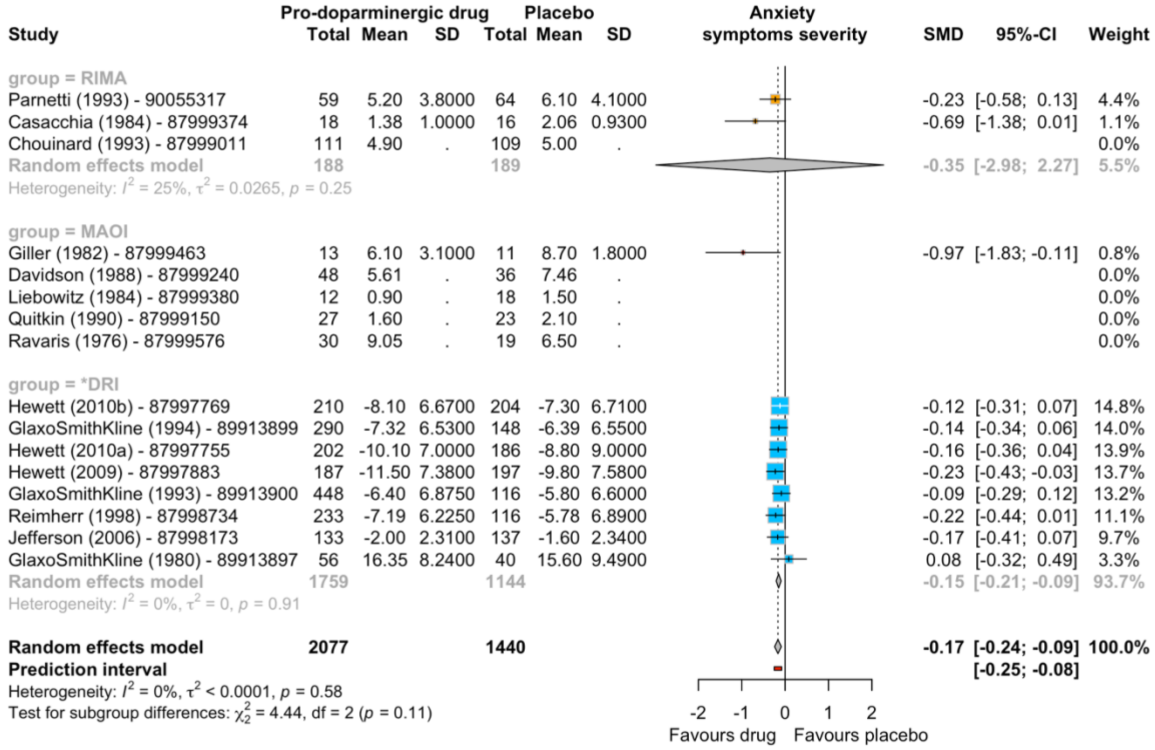

Figure 6 Forest plot for symptoms of anxiety (secondary outcome) comparing pro-dopaminergic interventions vs placebo for individuals with anxiety at 4-12 weeks (primary timepoint). SMD: standardised mean difference, 95% CI: 95% confidence intervals, SD: standard deviation. RIMA: Reversible inhibitors of monoamine oxidase-A, MAOI: monoamine oxidase inhibitors, DRI: dopamine reuptake inhibitor.

2.4.2 Risk of Bias

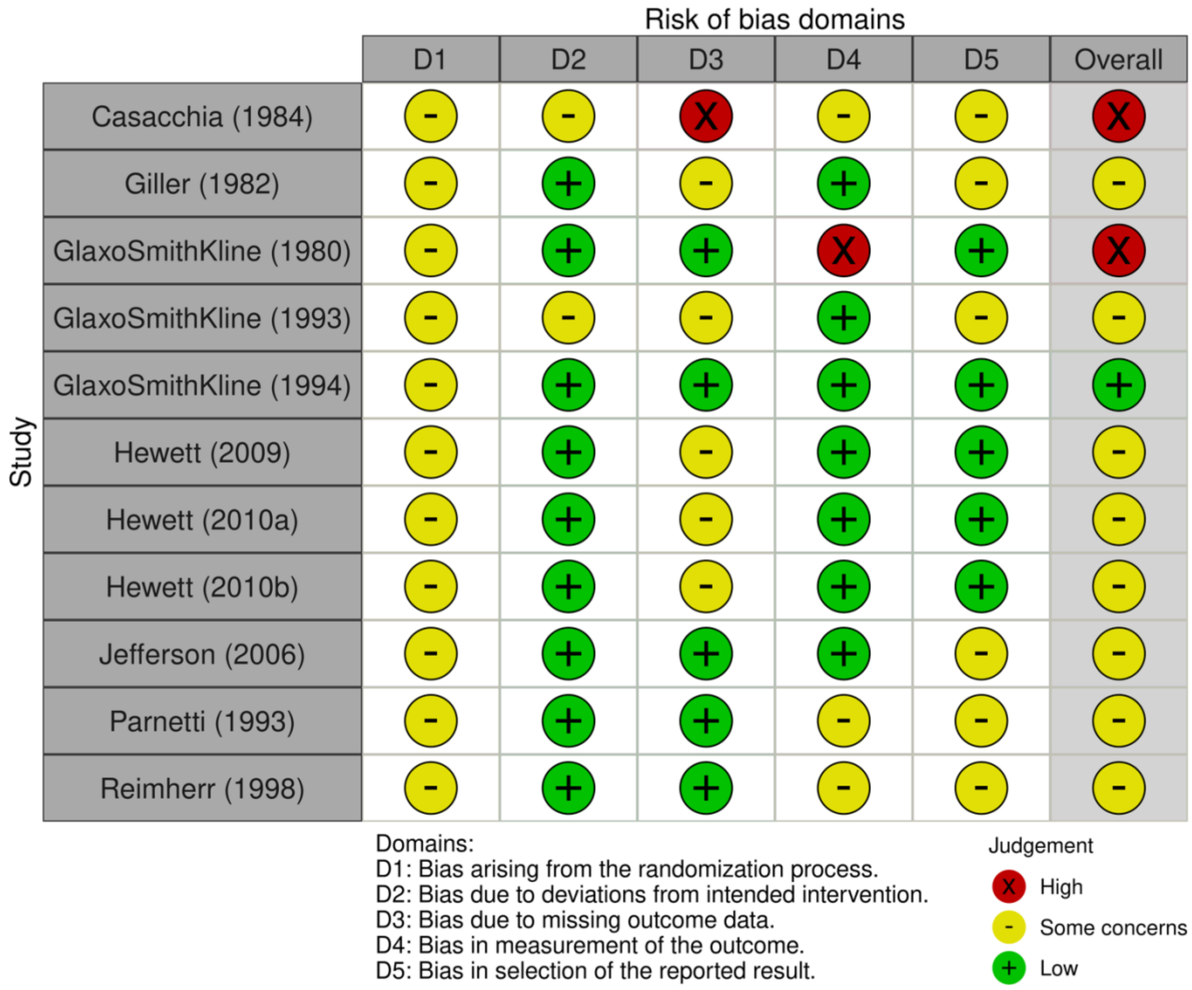

Figure 7. Risk of bias assessment.

2.4.3 Meta-regression analyses

The table below shows which of the covariates could modify the treatment effect of pro-dopaminergic interventions on anxiety symptom severity.

	SMD	95% CI	τ^2
Unadjusted effect	-0.166	-0.243 to -0.088	0
**Moderator**		**95% CI**	τ^2
Anxiety baseline (per point increase)	0.01	-0.01 to 0.03	0
Age (per 10 year increase)	0	-0.06 to 0.06	0
Female proportion (per percentage point increase)	-0.15	-1.85 to 1.55	0
Treatment duration (per week increase)	0.01	-0.05 to 0.08	0

2.5 Secondary outcome: Dropouts due to any reason

2.5.1 Pairwise meta-analysis

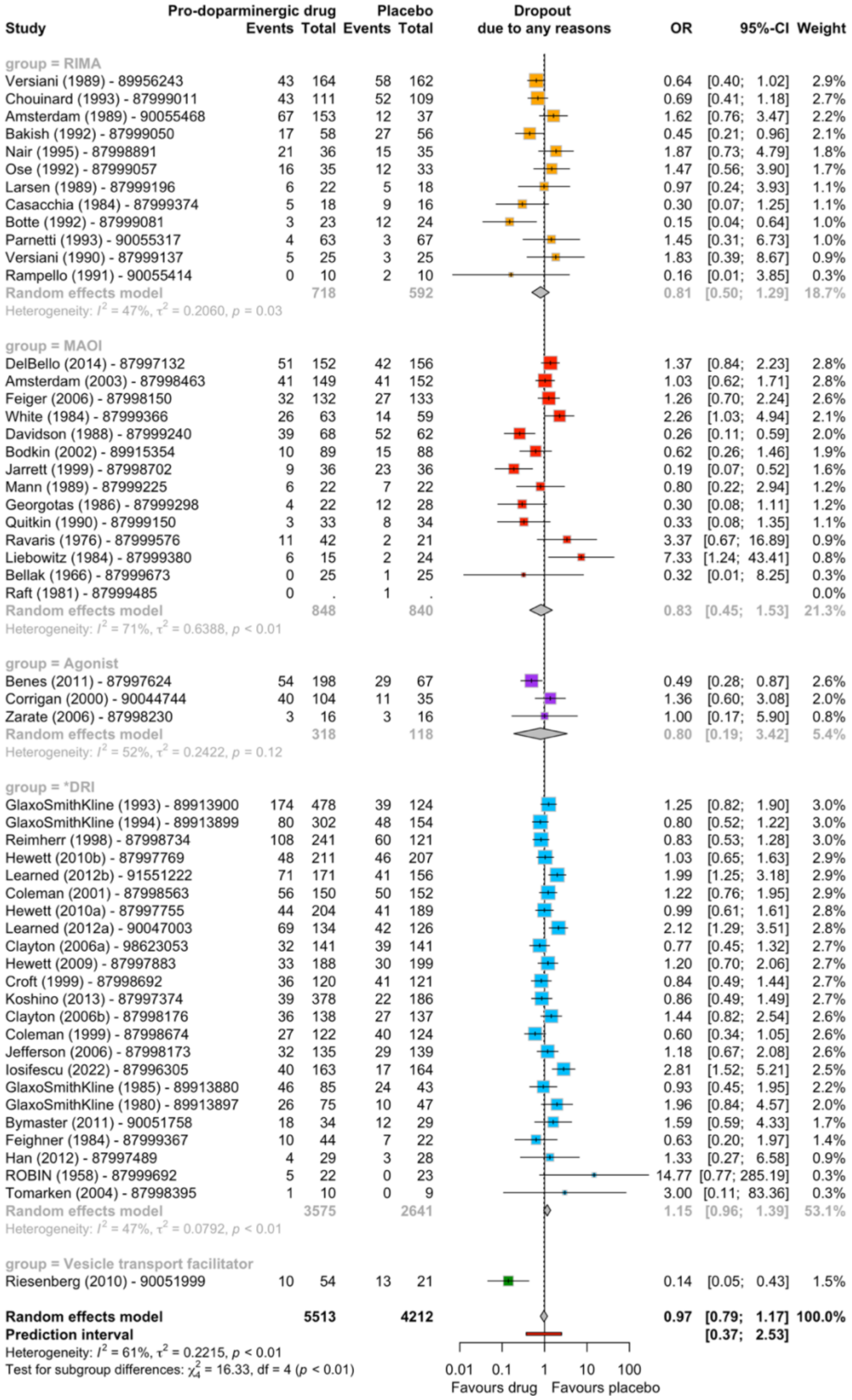

Figure 8 Forest plot for dropouts due to any reason for the comparison of pro-dopaminergic interventions vs placebo at 4-12 weeks. OR: odds ratio, 95% CI: 95% confidence intervals.RIMA:

Reversible inhibitors of monoamine oxidase-A, MAOI: monoamine oxidase inhibitors, DRI: dopamine reuptake inhibitor.

2.5.2 Risk of bias

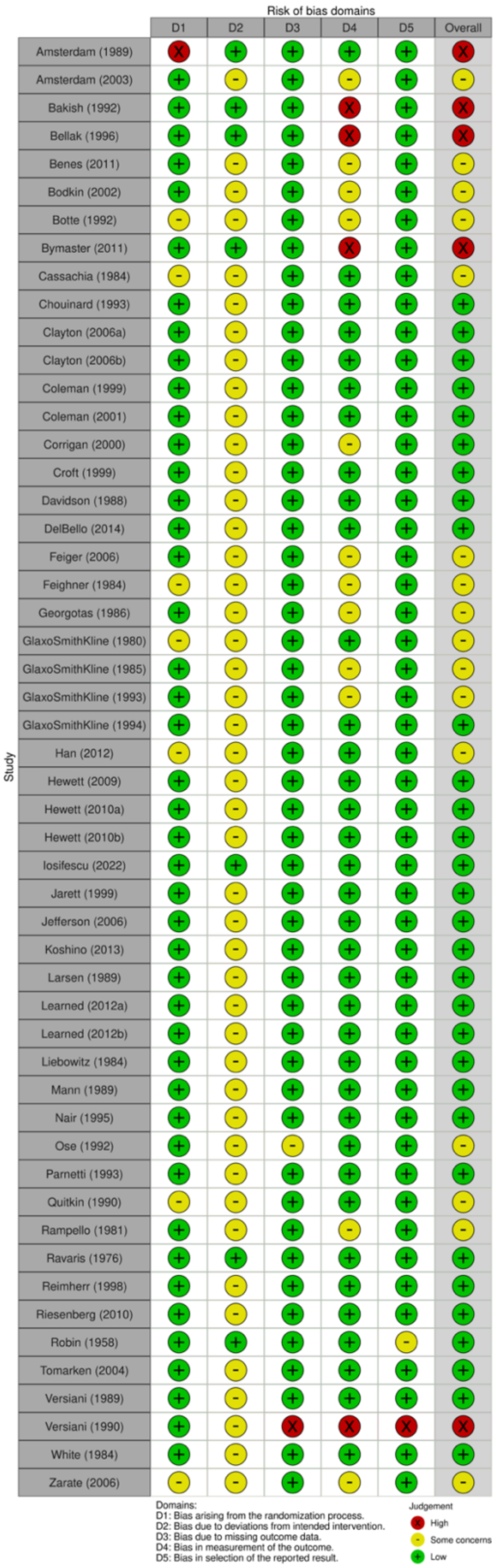

2.5.3 Meta-regression analyses

Figure 9. Risk of bias assessment.

The table below shows which of the covariates, if any, explain some of the heterogeneity (τ^2) observed in the effect sizes of the effect of pro-dopaminergic interventions on acceptability.

	OR	95% CI	τ^2
Unadjusted effect	0.965	0.794 to 1.172	0.22
**Moderator**		**95% CI**	τ^2
Anxiety baseline (per point increase)	1.03	0.99 to 1.07	0.01
Age (per 10 year increase)	0.88	0.71 to 1.09	0.24
Female proportion (per percentage point increase)	0.44	0.09 to 2.13	0.22
Treatment duration (per week increase)	0.99	0.9 to 1.1	0.25

The smaller τ^2 value for anxiety baseline suggests that this variable seem to explain the heterogeneity.

2.6 Secondary outcome: dropouts due to side effects

2.6.1 Pairwise meta-analysis

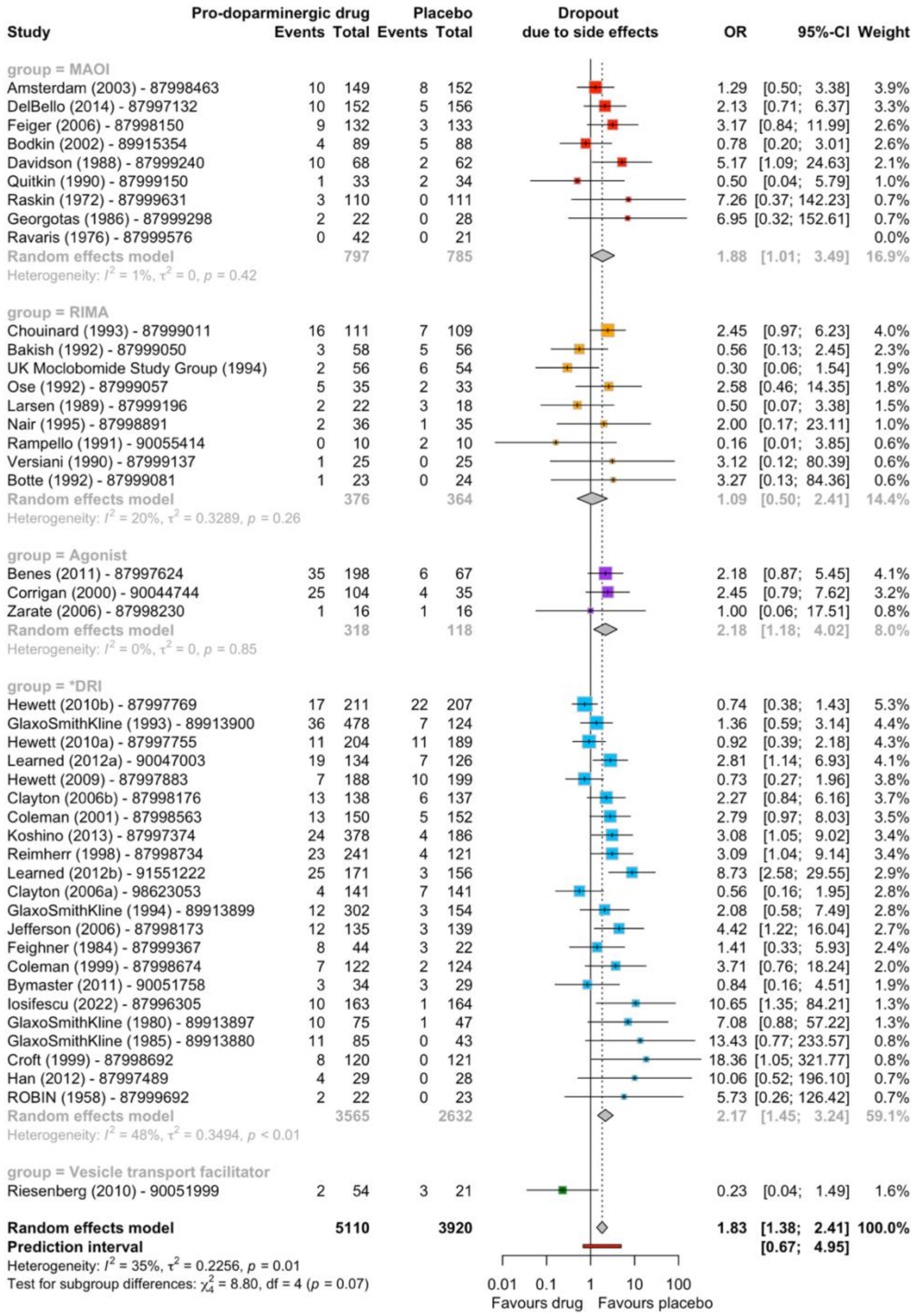

Figure 10 Forest plot for dropouts due to adverse events for the comparison of pro-dopaminergic interventions vs placebo at 4-12 weeks. OR: odds ratio, 95% CI: 95% confidence intervals.RIMA:

Reversible inhibitors of monoamine oxidase-A, MAOI: monoamine oxidase inhibitors, DRI: dopamine reuptake inhibitor.

2.6.2 Risk of bias

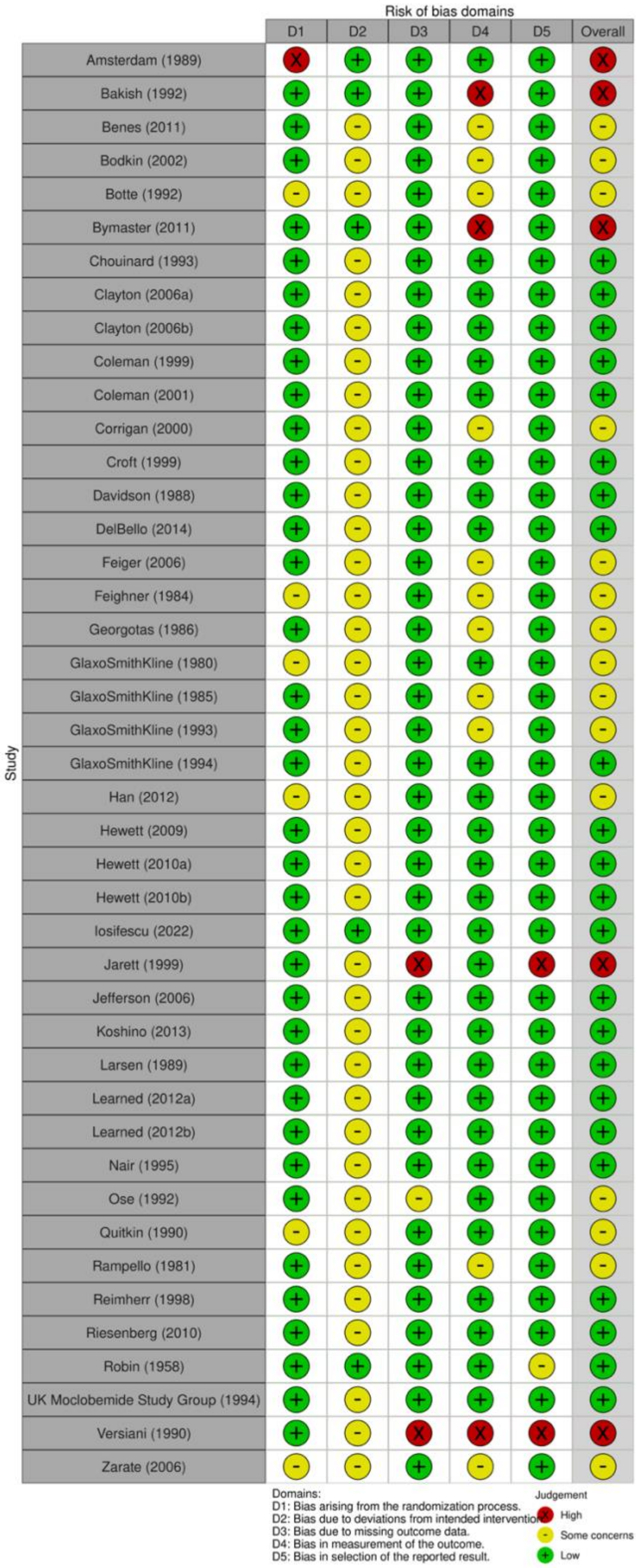

Figure 11. Risk of bias assessment.

2.6.3 Meta-regression analyses

The table below shows which of the covariates, if any, explain some of the heterogeneity (τ^2) observed in the effect sizes of the effect of pro-dopaminergic interventions on tolerability.

	**OR**	**95% CI**	τ^2
Unadjusted effect	1.825	1.382 to 2.41	0.23
**Moderator**		**95% CI**	τ^2
Anxiety baseline (per point increase)	0.93	0.86 to 1.01	0.01
Age (per 10 year increase)	0.81	0.62 to 1.06	0.17
Female proportion (per percentage point increase)	0.06	0.01 to 0.51	0.08
Treatment duration (per week increase)	1.03	0.89 to 1.2	0.26

The smaller τ^2 values for anxiety baseline and female proportion suggest that these predictors seem to explain the heterogeneity.

2.7 Secondary outcome: nausea

2.7.1 Pairwise meta-analysis

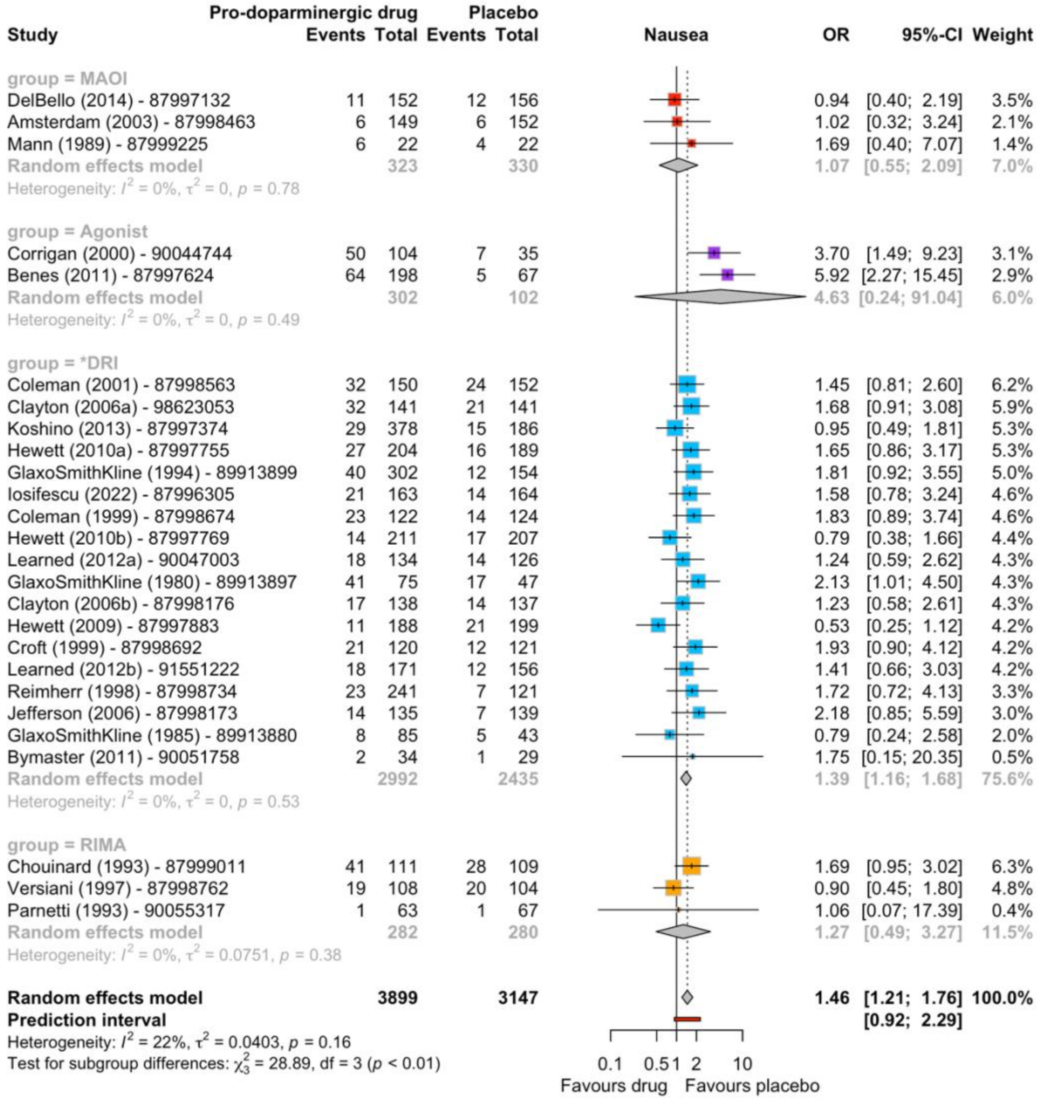

Figure 12 Forest plot for nausea for the comparison of pro-dopaminergic interventions vs placebo at 4-12 weeks. OR: odds ratio, 95% CI: 95% confidence intervals. RIMA: Reversible inhibitors of monoamine oxidase-A, MAOI: monoamine oxidase inhibitors, DRI: dopamine reuptake inhibitor.

2.7.2 Risk of bias

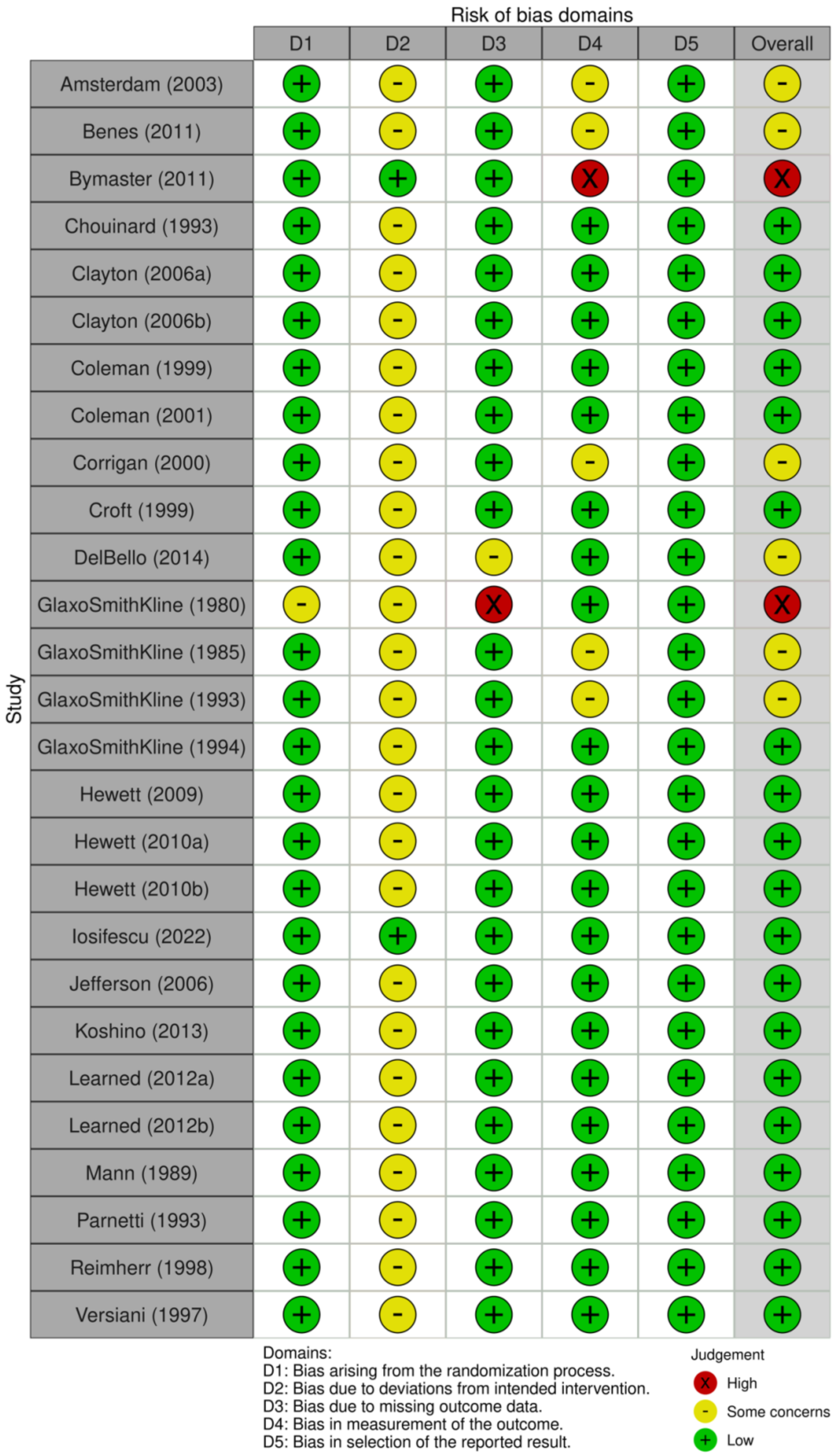

Figure 13. Risk of bias assessment.

2.7.3 Meta-regression analyses

The table below shows which of the covariates, if any, explain some of the heterogeneity (τ^2) observed in the effect sizes of the effect of pro-dopaminergic interventions on nausea.

	**OR**	**95% CI**	**τ^2**
Unadjusted effect	1.455	1.206 to 1.756	0.04
**Moderator**		**95% CI**	**τ^2**
Age (per 10 year increase)	1	0.82 to 1.22	0.03
Female proportion (per percentage point increase)	1.11	0.2 to 6.09	0.03
Treatment duration (per week increase)	1.01	0.9 to 1.13	0.06

2.8 Secondary outcome: headache

2.8.1 Pairwise meta-analysis

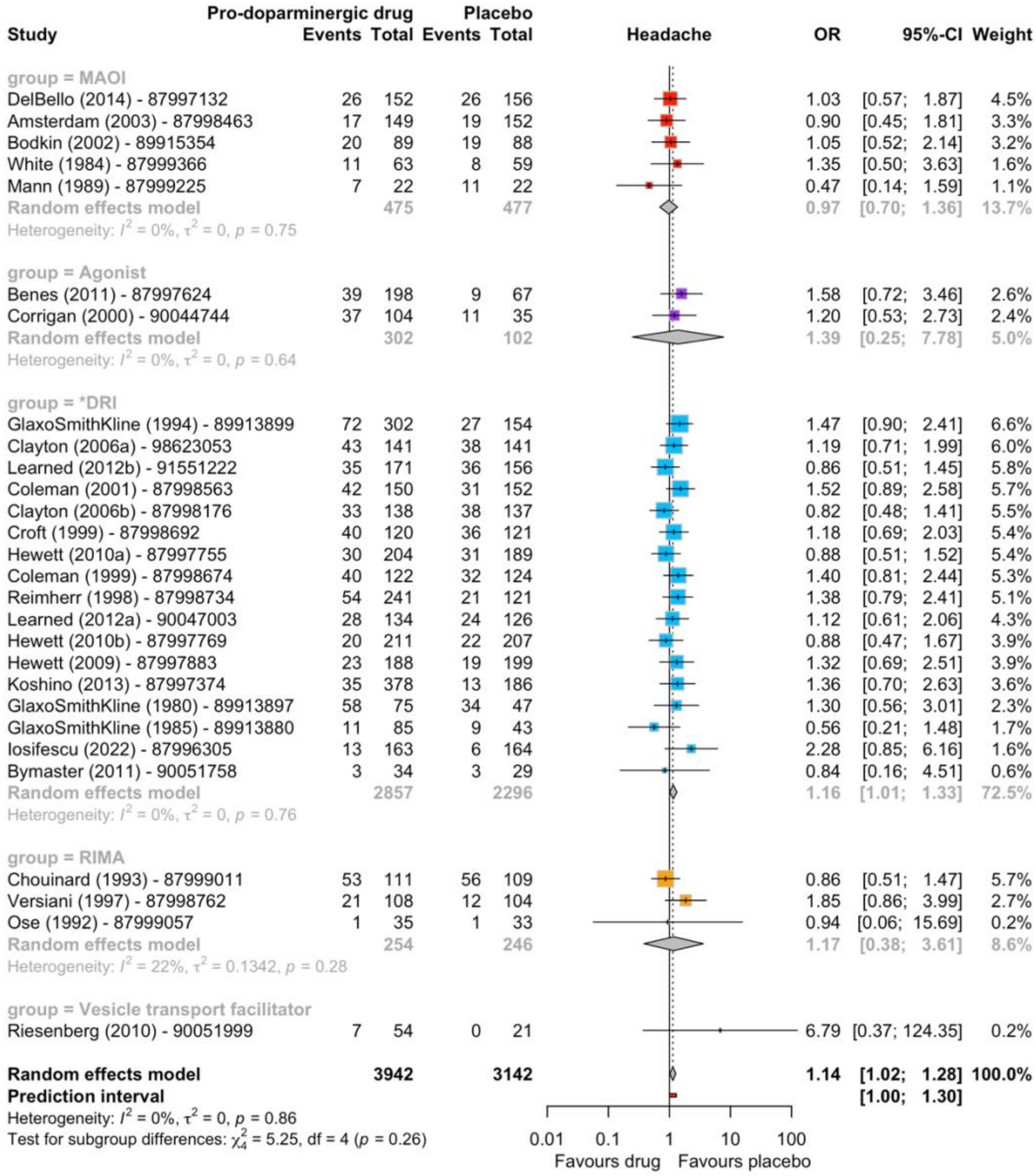

Figure 14 Forest plot for headaches for the comparison of pro-dopaminergic interventions vs placebo at 4-12 weeks. OR: odds ratio, 95% CI: 95% confidence intervals. RIMA: Reversible inhibitors of monoamine oxidase-A, MAOI: monoamine oxidase inhibitors, DRI: dopamine reuptake inhibitor.

2.8.2 Risk of bias

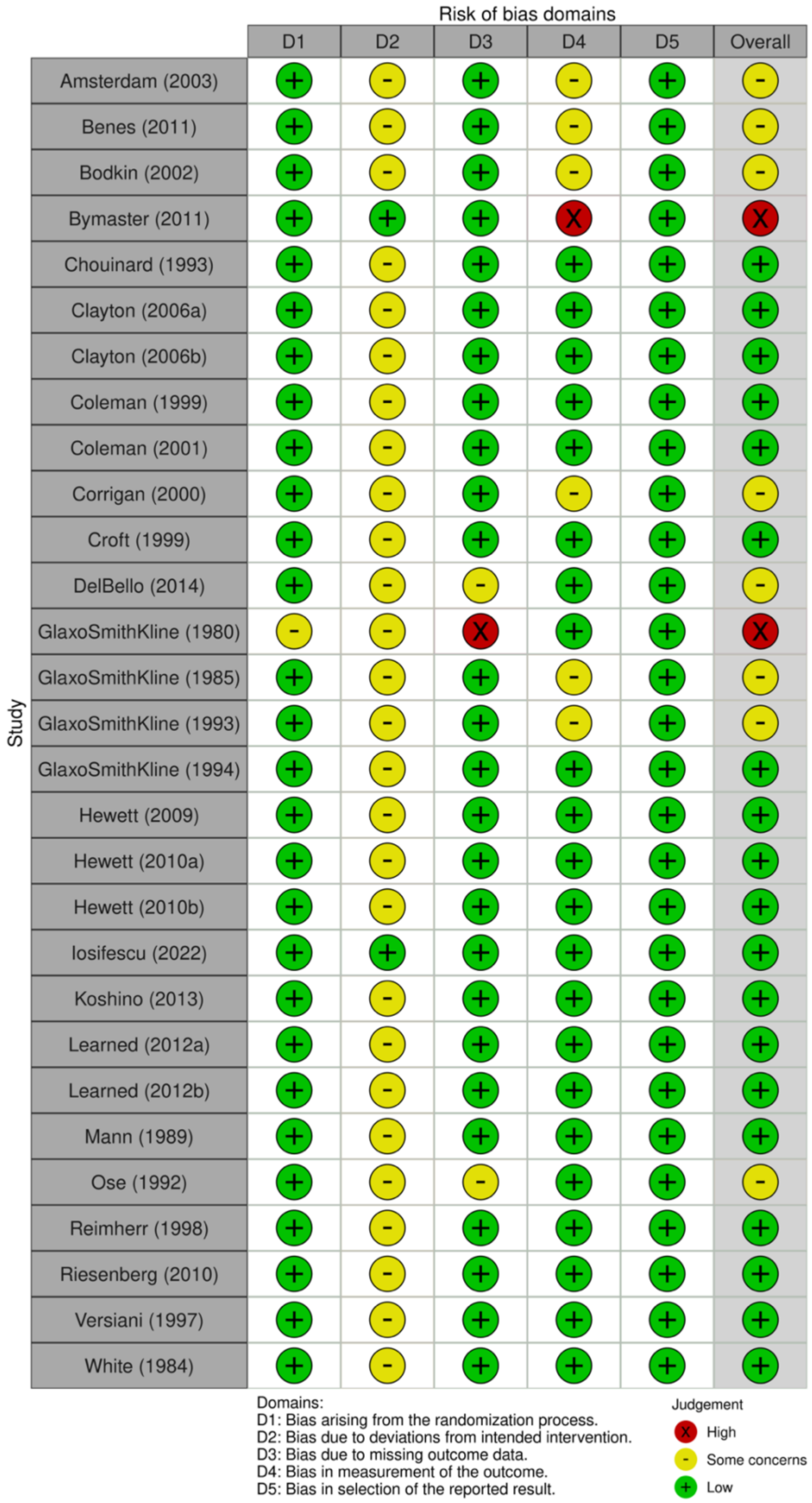

Figure 15. Risk of bias assessment.

2.8.3 Meta-regression analyses

The table below shows which of the covariates, if any, could modify the relative treatment effect of pro-dopaminergic interventions on headache. We did not perform a meta-regression on mean anhedonia and anxiety baseline scores as the total number of studies was below 10.

	**OR**	**95% CI**	**τ^2**
Unadjusted effect	1.14	1.019 to 1.275	0
**Moderator**		**95% CI**	**τ^2**
Age (per 10 year increase)	0.95	0.84 to 1.08	0
Female proportion (per percentage point increase)	1.71	0.64 to 4.56	0
Treatment duration (per week increase)	1.01	0.94 to 1.07	0

2.9 Secondary outcome: insomnia

2.9.1 Pairwise meta-analysis

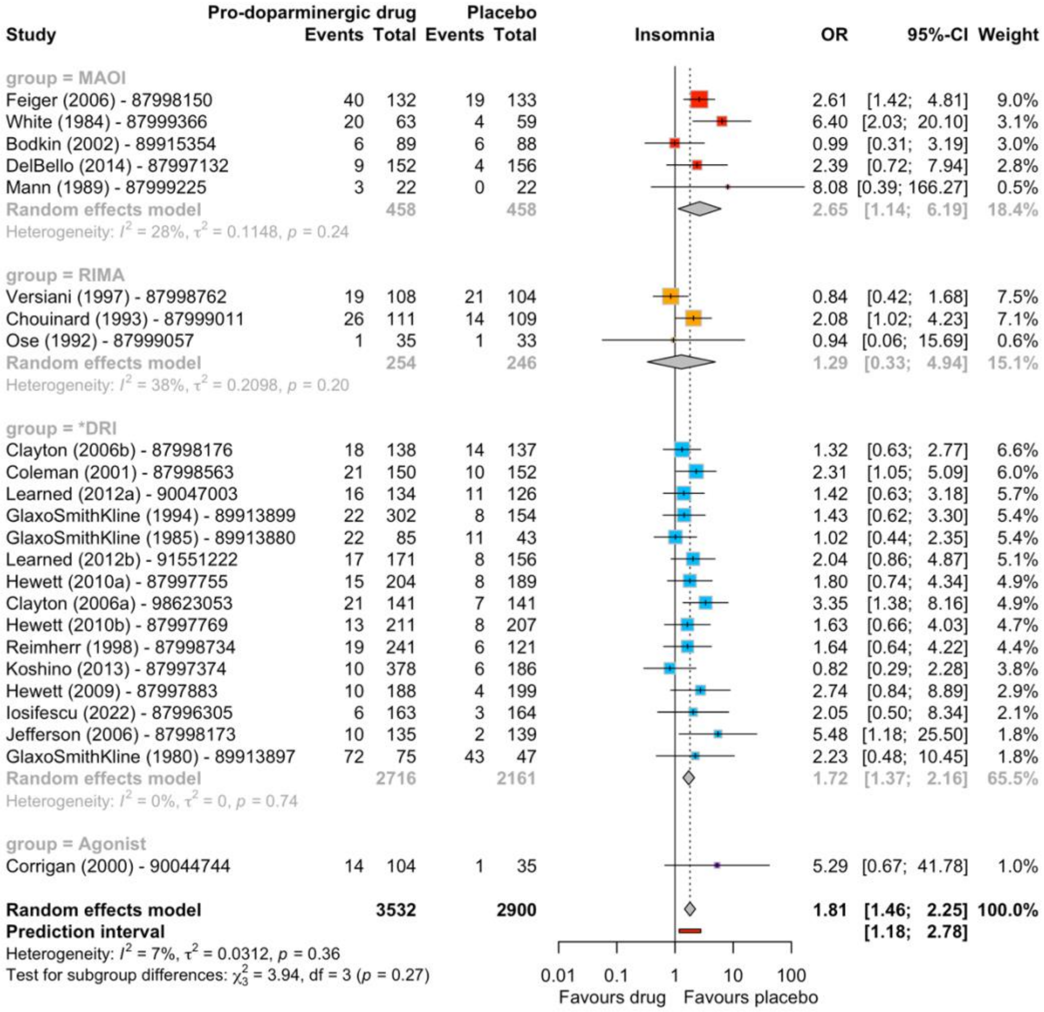

Figure 16 Forest plot for insomnia for the comparison of pro-dopaminergic interventions vs placebo at 4-12 weeks. OR: odds ratio, 95% CI: 95% confidence intervals. RIMA: Reversible inhibitors of monoamine oxidase-A, MAOI: monoamine oxidase inhibitors, DRI: dopamine reuptake inhibitor.

2.9.2 Risk of bias

Study	D1	D2	D3	D4	D5	Overall
Bodkin (2002)	+	-	+	-	+	-
Chouinard (1993)	+	-	+	+	+	+
Clayton (2006a)	+	-	+	+	+	+
Clayton (2006b)	+	-	+	+	+	+
Coleman (2001)	+	-	+	+	+	+
Corrigan (2000)	+	-	+	-	+	-
DelBello (2014)	+	-	-	+	+	-
Feiger (2006)	+	-	+	-	+	-
GlaxoSmithKline (1980)	-	-	X	+	+	X
GlaxoSmithKline (1985)	+	-	+	-	+	-
GlaxoSmithKline (1993)	+	-	+	-	+	-
GlaxoSmithKline (1994)	+	-	+	+	+	+
Hewett (2009)	+	-	+	+	+	+
Hewett (2010a)	+	-	+	+	+	+
Hewett (2010b)	+	-	+	+	+	+
Iosifescu (2022)	+	+	+	+	+	+
Jefferson (2006)	+	-	+	+	+	+
Koshino (2013)	+	-	+	+	+	+
Learned (2012a)	+	-	+	+	+	+
Learned (2012b)	+	-	+	+	+	+
Mann (1989)	+	-	+	+	+	+
Ose (1992)	+	-	-	+	+	-
Reimherr (1998)	+	-	+	+	+	+
Versiani (1997)	+	-	+	+	+	+
White (1984)	+	-	+	+	+	+

Risk of bias domains

Domains:
D1: Bias arising from the randomization process.
D2: Bias due to deviations from intended intervention.
D3: Bias due to missing outcome data.
D4: Bias in measurement of the outcome.
D5: Bias in selection of the reported result.

Judgement
X High
- Some concerns
+ Low

Figure 17. Risk of bias assessment.

2.9.3 Meta-regression analyses

The table below shows which of the covariates, if any, explain some of the heterogeneity (τ^2) observed in the effect sizes of the effect of pro-dopaminergic interventions on insomnia.

	OR	95% CI	τ^2
Unadjusted effect	1.809	1.458 to 2.246	0.03
**Moderator**		**95% CI**	τ^2
Age (per 10 year increase)	0.91	0.7 to 1.17	0.04
Female proportion (per percentage point increase)	0.83	0.13 to 5.2	0.04
Treatment duration (per week increase)	1.01	0.89 to 1.14	0.05

2.10 Secondary outcome: constipation

2.10.1 Pairwise meta-analysis

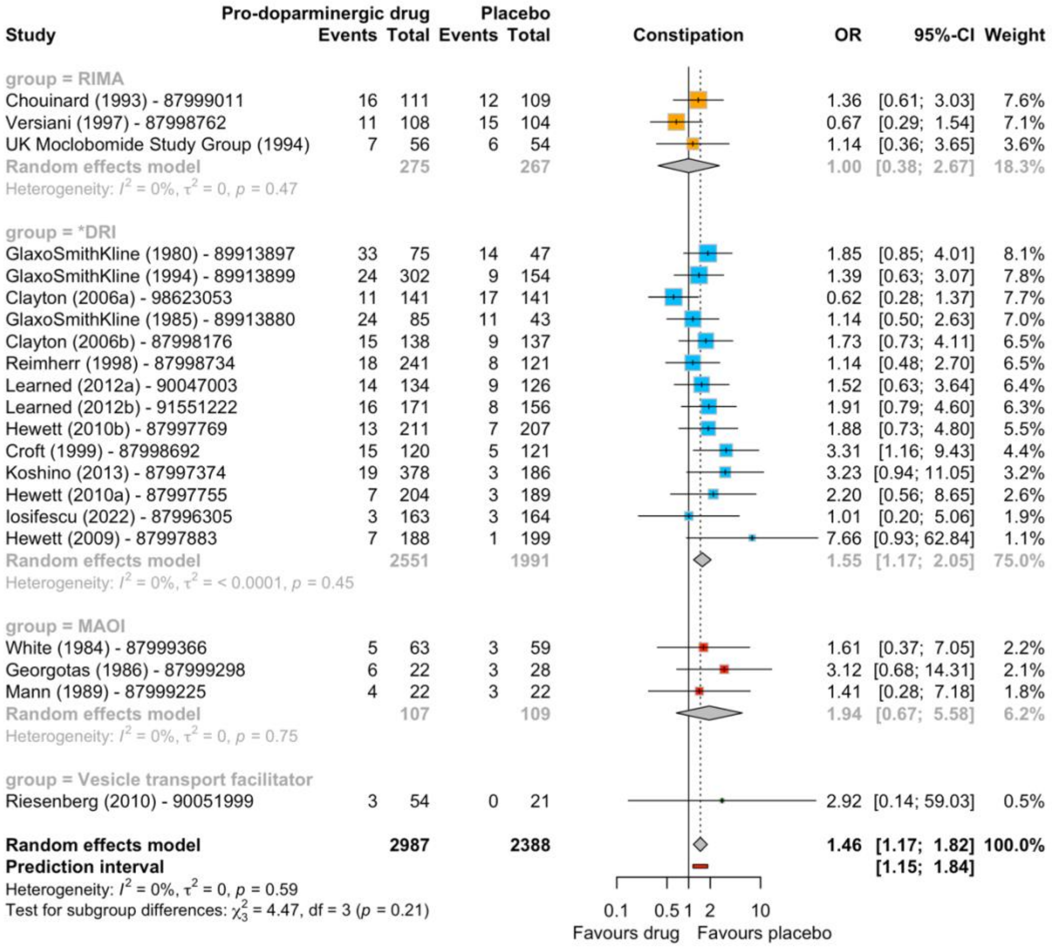

Figure 18 Forest plot for constipation for the comparison of pro-dopaminergic interventions vs placebo at 8 (4-12) weeks. OR: odds ratio, 95% CI: 95% confidence intervals. RIMA: Reversible inhibitors of monoamine oxidase-A, MAOI: monoamine oxidase inhibitors, DRI: dopamine reuptake inhibitor.

2.10.2 Risk of bias

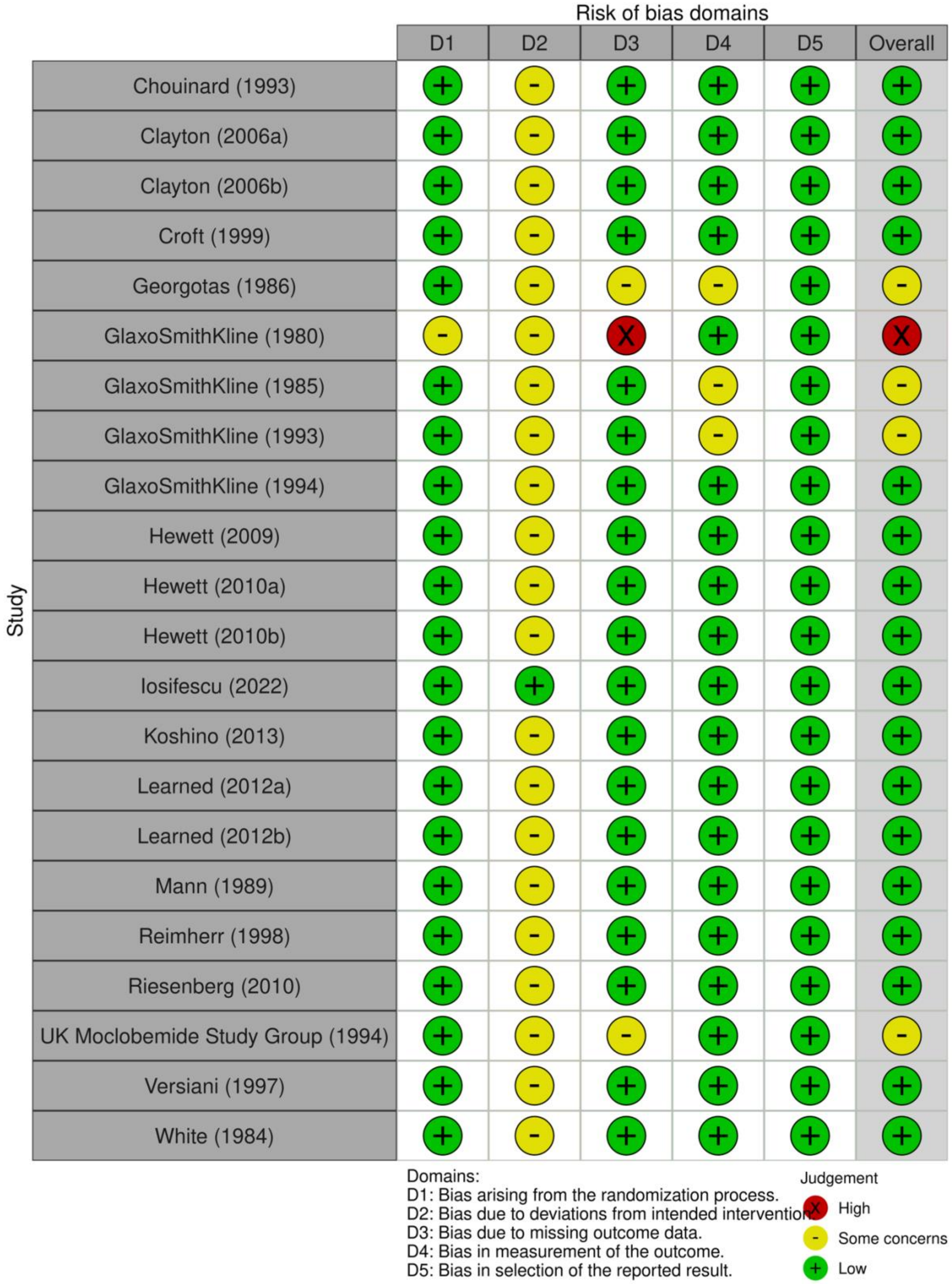

Figure 19. Risk of bias assessment.

2.10.3 Meta-regression analyses

The table below shows which of the covariates, if any, could modify the relative treatment effect of pro-dopaminergic interventions on constipation.

	**OR**	**95% CI**	$\boldsymbol{\tau^2}$
Unadjusted effect	1.456	1.166 to 1.818	0
**Moderator**		**95% CI**	$\boldsymbol{\tau^2}$
Age (per 10 year increase)	1.1	0.84 to 1.44	0.01
Female proportion (per percentage point increase)	0.42	0.07 to 2.6	0
Treatment duration (per week increase)	1.09	0.96 to 1.24	0.01

2.11 Secondary outcome: dizziness

2.12.1 Pairwise meta-analysis

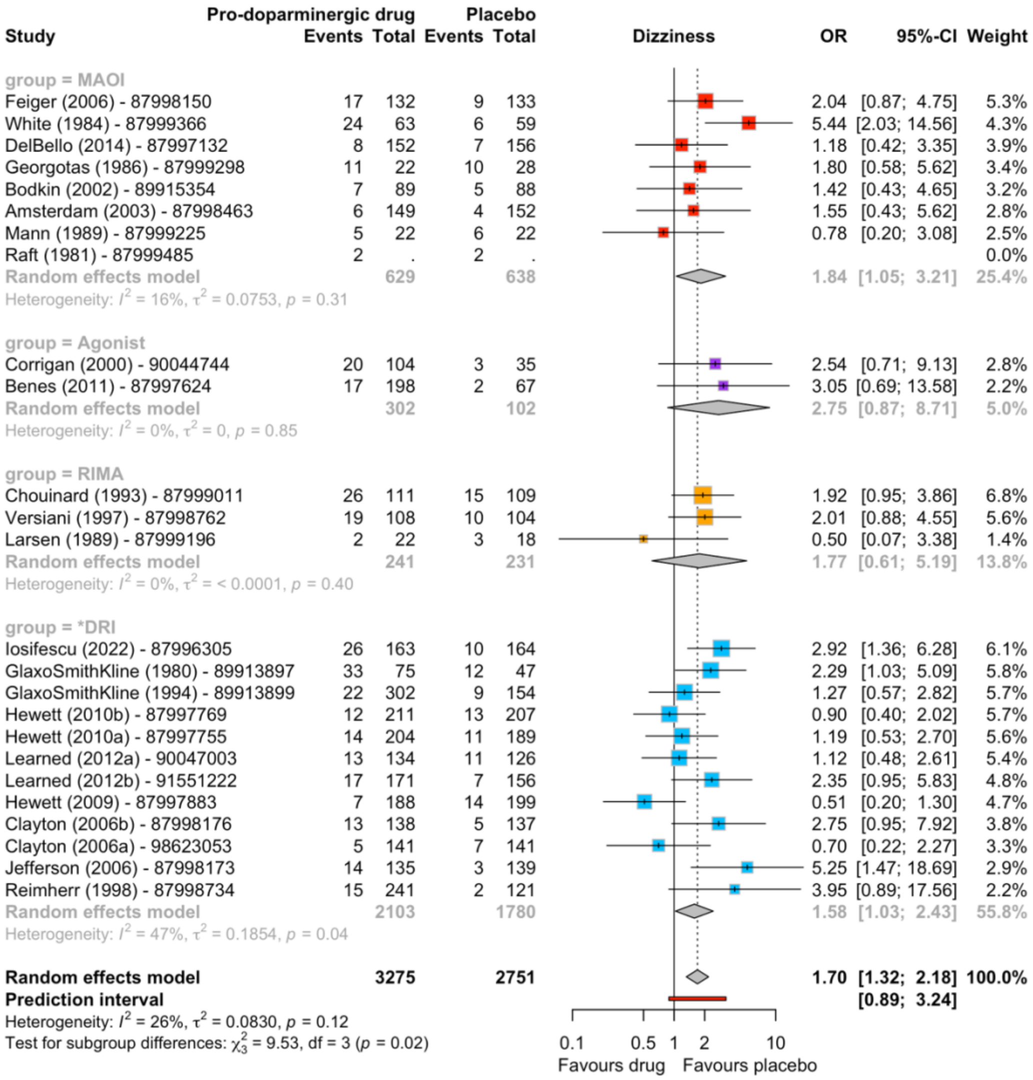

Figure 20 Forest plot for dizziness for the comparison of pro-dopaminergic interventions vs placebo at 8 (4-12) weeks.

OR: odds ratio, 95% CI: 95% confidence intervals. RIMA: Reversible inhibitors of monoamine oxidase-A, MAOI: monoamine oxidase inhibitors, DRI: dopamine reuptake inhibitor.

2.11.2 Risk of bias

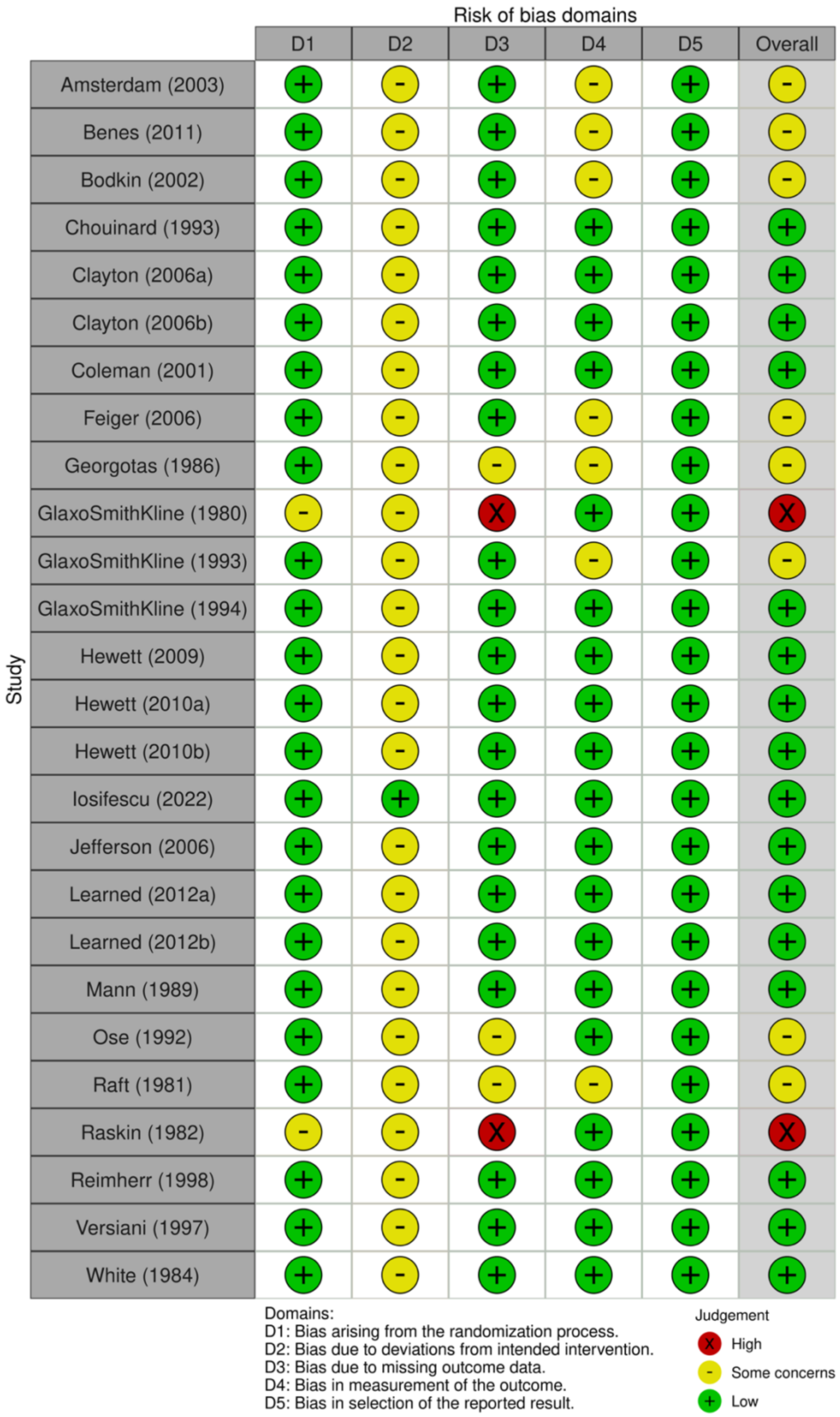

Figure 21. Risk of bias assessment.

2.11.3 Meta-regression analyses

The table below shows which of the covariates, if any, explain some of the heterogeneity (τ^2) observed in the effect sizes of the effect of pro-dopaminergic interventions on dizziness.

	OR	95% CI	τ^2
Unadjusted effect	1.697	1.319 to 2.184	0.08
**Moderator**		**95% CI**	τ^2
Age (per 10 year increase)	0.92	0.72 to 1.18	0.09
Female proportion (per percentage point increase)	0.18	0.03 to 1.29	0.06
Treatment duration (per week increase)	0.9	0.79 to 1.03	0.04

The smaller τ^2 values for female proportion and treatment duration suggest that these predictors may explain some of the heterogeneity.

2.12 Secondary outcome: dry mouth

2.12.1 Pairwise meta-analysis

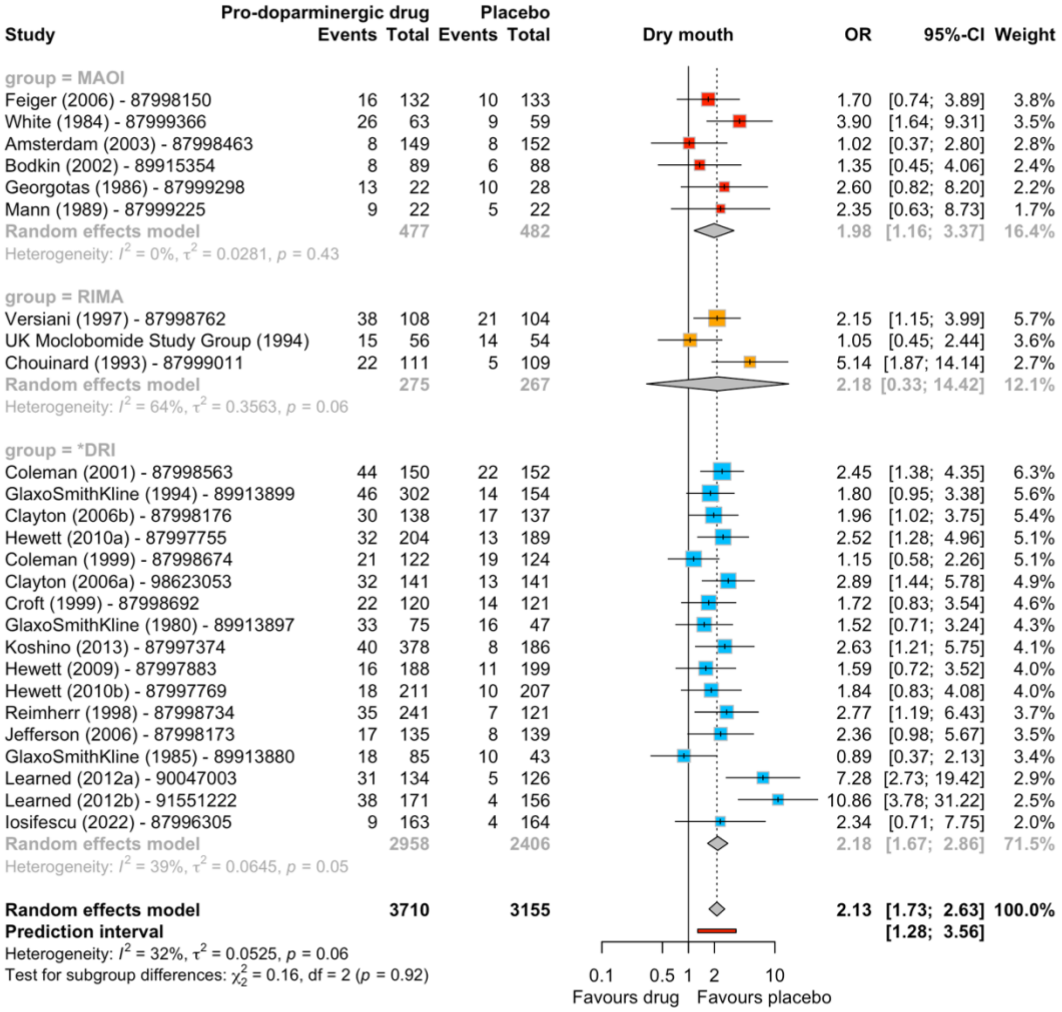

Figure 22 Forest plot for dry mouth for the comparison of pro-dopaminergic interventions vs placebo at 4-12 weeks. OR: odds ratio, 95% CI: 95% confidence intervals. RIMA: Reversible inhibitors of monoamine oxidase-A, MAOI: monoamine oxidase inhibitors, DRI: dopamine reuptake inhibitor.

2.12.2 Risk of bias

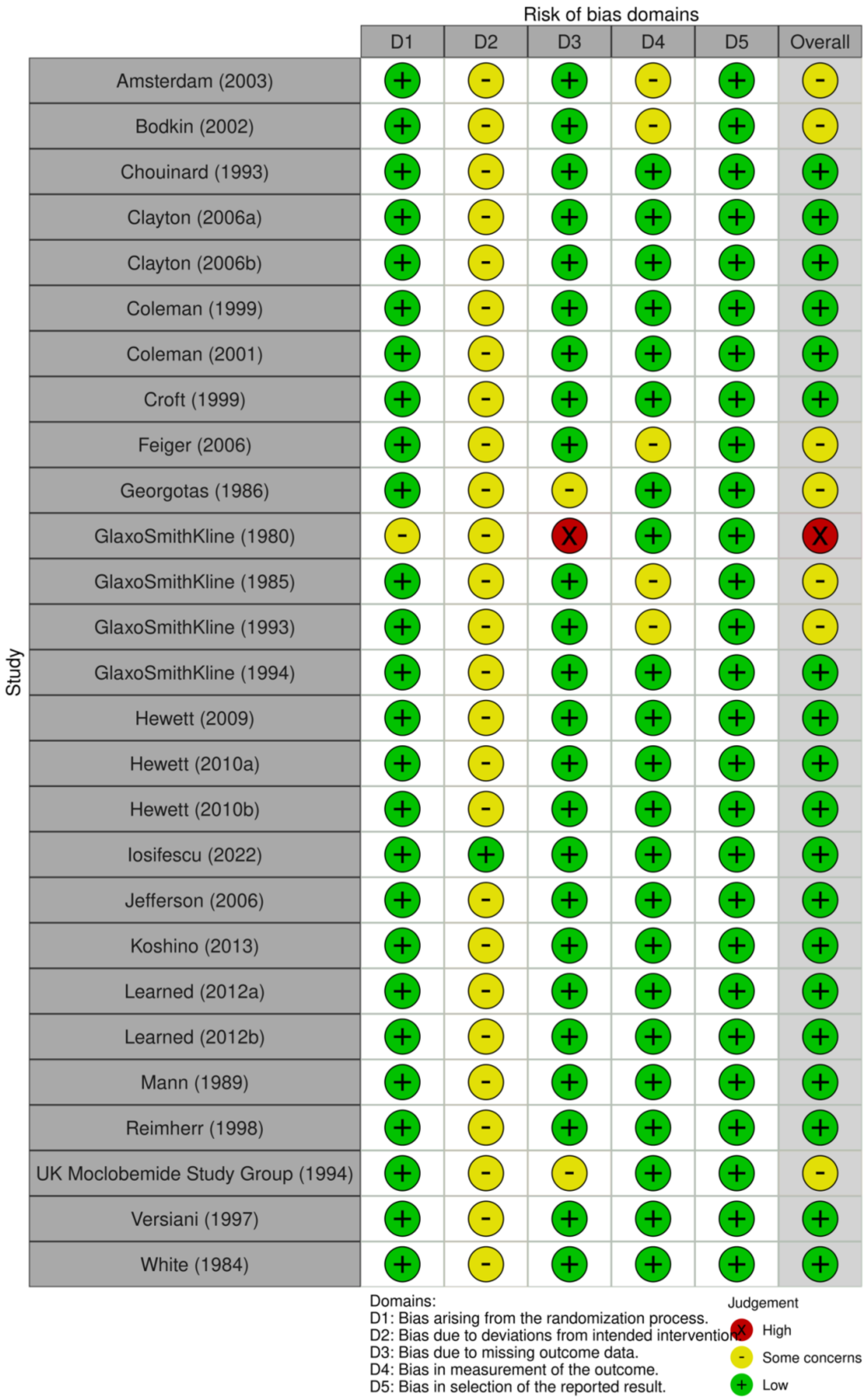

Figure 23. Risk of bias assessment.

2.12.3 Meta-regression analyses

The table below shows which of the covariates, if any, explain some of the heterogeneity (τ^2) observed in the effect sizes of the effect of pro-dopaminergic interventions on dry mouth.

	OR	95% CI	τ^2
Unadjusted effect	2.134	1.731 to 2.631	0.05
**Moderator**	ββ	**95% CI**	τ^2
Age (per 10 year increase)	0.95	0.72 to 1.24	0.05
Female proportion (per percentage point increase)	0.14	0.03 to 0.76	0.02
Treatment duration (per week increase)	1.11	0.97 to 1.27	0.06

The smaller τ^2 value for female proportion suggests that this predictor seems to explain some of the heterogeneity.

2.13 Secondary outcome: vomiting

2.13.1 Pairwise meta-analysis

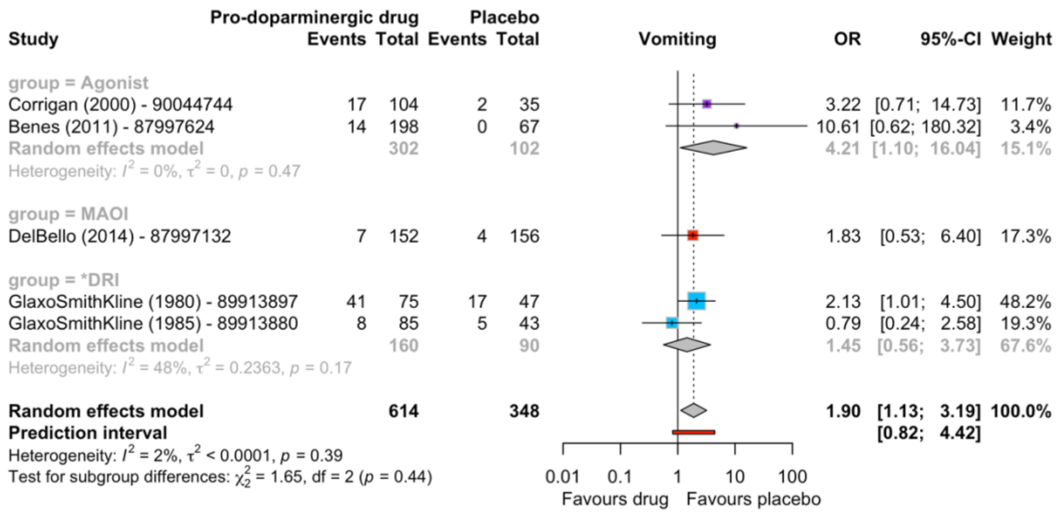

Figure 24 Forest plot for vomiting for the comparison of pro-dopaminergic interventions vs placebo at 4-12 weeks. OR: odds ratio, 95% CI: 95% confidence intervals. RIMA: Reversible inhibitors of monoamine oxidase-A, MAOI: monoamine oxidase inhibitors, DRI: dopamine reuptake inhibitor.

2.13.2 Risk of bias

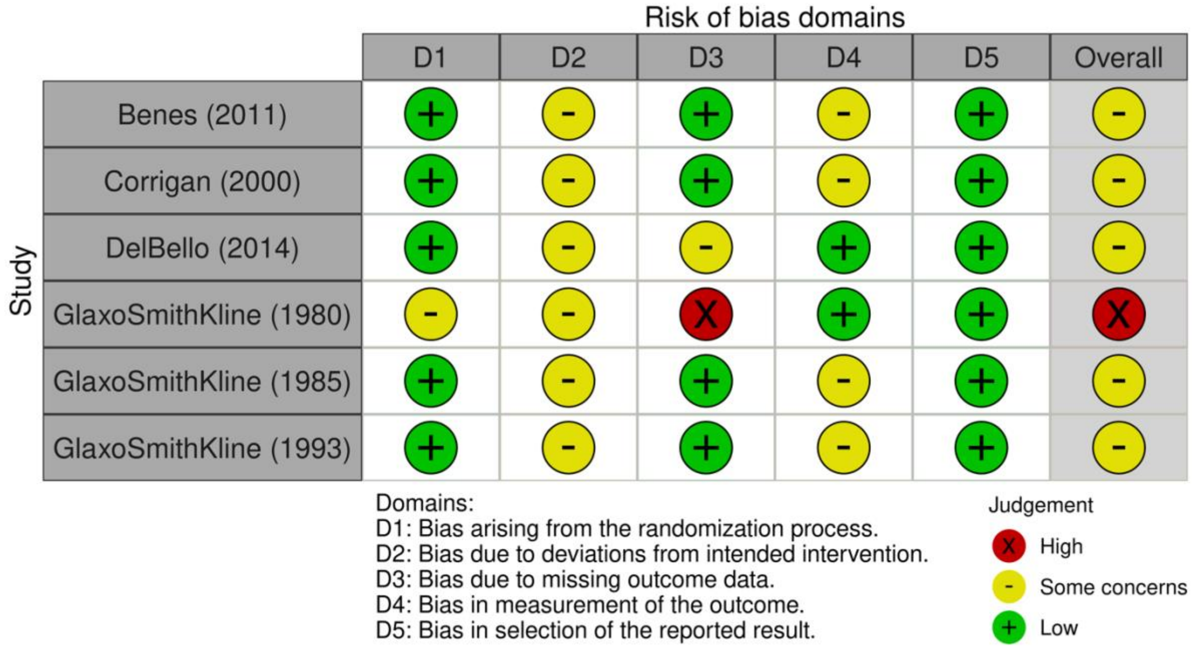

Figure 25. Risk of bias assessment.

2.13.3 Meta-regression analyses

We did not perform any meta-regressions as the total number of studies was below 10.

2.14 Summary of evidence tables

Source of Evidence (Human studies)	Outcome	Timepoint	Summary of the association	Bias due to study limitations	Reporting bias	Indirectness	Bias due to other reasons
Studies on pro-dopaminergic interventions vs placebo	Symptoms of anhedonia	6-10 weeks	N=6, n=2076; SMD= -0.24, 95% CI: -0.46, -0.03 95% PrI: -0.77, 0.34	Moderate risk: 2 studies had high risk of bias, 2 had a moderate risk of bias (Figure 3). The impact of bias on the magnitude and direction of the effects of pro-dopaminergic interventions is unclear.	Low Risk	Moderate risk: 3 of the 6 studies used the Motivation and Energy Inventory, 2 used a single MADRS anhedonia item, and 1 used the IDS-IVR-30/IDS-C-30 Pleasure Scale. As anhedonia is a complex construct, “pleasure” scales and single items may fail at capturing the complexity of anhedonia in the context of depression. The impact of	No clear indication of other biases.

Source of Evidence (Human studies)	Outcome	Timepoint	Summary of the association	Bias due to study limitations	Reporting bias	Indirectness	Bias due to other reasons
						indirectness its direction is unclear.	
Direct and indirect evidence for pro-dopaminergic interventions (i.e., bupropion) vs placebo	Symptoms of anhedonia (MADRS anhedonia item)	6-12 weeks	N=34, n=14054; SMD= -0.12, 95% CI: -0.25 to 0.00	NA	NA	NA	NA
Direct and indirect evidence for non-dopaminergic interventions vs placebo	Symptoms of anhedonia (MADRS anhedonia item)	6-12 weeks	N=34, n=14054; SMD ranges from -0.05 (95% CI: -0.19 to 0.08) to –0.40 (95% CI: -0.48 to –0.32)	NA	NA	NA	NA
Direct and indirect evidence of pro-dopaminergic interventions (i.e. buproprion)	Symptoms of anhedonia (MADRS anhedonia item)	6-12 weeks	N=34, n=14054; SMD ranges from -0.28 (95% CI: -0.15 to 0.41) to -	NA	NA	NA	NA

Source of Evidence (Human studies)	Outcome	Timepoint	Summary of the association	Bias due to study limitations	Reporting bias	Indirectness	Bias due to other reasons
vs non-dopaminergic			0.07 (95% CI: -0.23 to 0.09)				
Studies on pro-dopaminergic interventions vs placebo	Anxiety symptoms	4-12 weeks	N=11, n=3517; SMD=-0.17, 95%CI: -0.24, -0.09 95%PrI: -0.25, -0.08	Moderate risk: 73% of studies were rated as having an overall moderate risk of bias while 18% were rated as high risk of bias.	Low Risk	Low risk: all studies included patients with depression receiving pro-dopaminergic interventions and assessed for anhedonia.	No clear indication of other biases.
Studies on pro-dopaminergic interventions vs placebo	Acceptability (dropout for any reason)	4-12 weeks	N=52, n=9725; OR=0.97, 95% CI: 0.79, 1.17 95% PrI: 0.37, 2.53	Moderate risk: 59% had a low overall risk of bias, 10% of studies were rated as having a high risk of bias, primarily due to concerns over outcome measurement, while 31% were	Moderate risk: The potential for missing studies across the review was deemed to be low in domain 3 of the RoB-ME assessment however it	Low risk: all studies included patients with depression receiving pro-dopaminergic interventions and assessed for anhedonia.	No clear indication of other biases.

Source of Evidence (Human studies)	Outcome	Timepoint	Summary of the association	Bias due to study limitations	Reporting bias	Indirectness	Bias due to other reasons
				rated moderate risk of bias.	was unclear whether some studies generated an eligible result.		
Studies on pro-dopaminergic interventions vs placebo	Tolerability (dropout for side effects)	4-12 weeks	N=43, n=9030; OR=1.83, 95% CI: 1.38, 2.41 95% PrI: 0.67, 4.95	Low risk: 53% had an overall low risk of bias, 12% of studies had an overall high risk of bias, primarily due to concerns over outcome measurement, while 35% were rated moderate risk of bias.	Moderate risk: The potential for missing studies across the review was deemed to be low in domain 3 of the RoB-ME assessment however it was unclear whether some studies generated an eligible result.	Low risk: all studies included patients with depression receiving pro-dopaminergic interventions and assessed for anhedonia.	No clear indication of other biases.

**Source of Evidence (Human studies)**	**Outcome**	**Timepoint**	**Summary of the association**	**Bias due to study limitations**	**Reporting bias**	**Indirectness**	**Bias due to other reasons**
Studies on pro-dopaminergic interventions vs placebo	Constipation reported for pro-dopaminergic interventions vs placebo	4-10 weeks	N=21, n=5375; OR=1.46, 95% CI: 1.17, 1.82 95% PrI: 1.15, 1.84	Low risk: 77% of studies were rated as having an overall low risk of bias and 5% were rated high risk of bias.	Moderate risk: The potential for missing studies across the review was deemed to be low in domain 3 of the RoB-ME assessment however it was unclear whether some studies generated an eligible result.	Low risk: all studies included patients with depression receiving pro-dopaminergic interventions and assessed for anhedonia.	No clear indication of other biases
Studies on pro-dopaminergic interventions vs placebo	Dizziness reported for pro-dopaminergic interventions vs placebo	4-12 weeks	N=24, n=6026; OR=1.70, 95% CI: 1.32, 2.18 95% PrI: 0.89, 3.24	Low risk: 59% of studies were rated as having an overall low risk of bias and only 8% were	Moderate risk: The potential for missing studies across the review was	Low risk: all studies included patients with depression receiving pro-dopaminergic interventions and	No clear indication of other biases

**Source of Evidence (Human studies)**	**Outcome**	**Timepoint**	**Summary of the association**	**Bias due to study limitations**	**Reporting bias**	**Indirectness**	**Bias due to other reasons**
				rated high risk of bias.	deemed to be low in domain 3 of the RoB-ME assessment however it was unclear whether some studies generated an eligible result.	assessed for anhedonia.	
Studies on pro-dopaminergic interventions vs placebo	Dry mouth reported for pro-dopaminergic interventions vs placebo	4-10 weeks	N=26, n=6865; OR=2.13, 95%CI: 1.73, 2.63, 95% PrI: 1.28, 3.56	Low risk: 67% of studies were rated low risk of bias for RoB2 while only 4% were rated high risk of bias.	Moderate risk: The potential for missing studies across the review was deemed to be low in domain 3 of the RoB-ME assessment however it was unclear	Low risk: all studies included patients with depression receiving pro-dopaminergic interventions and assessed for anhedonia.	No clear indication of other biases

Source of Evidence (Human studies)	Outcome	Timepoint	Summary of the association	Bias due to study limitations	Reporting bias	Indirectness	Bias due to other reasons
					whether some studies generated an eligible result.		
Studies on pro-dopaminergic interventions vs placebo	Headache reported for pro-dopaminergic interventions vs placebo	4-12 weeks	N=28, n=7084; OR=1.14, 95%CI: 1.02, 1.28, 95% PrI: 1.00, 1.30	Low risk: 65% of studies were rated low risk for RoB2 while 31% were rated as moderate risk with scores of 'some concerns' spread out across domains in no clear pattern.	Moderate risk: The potential for missing studies across the review was deemed to be low in domain 3 of the RoB-ME assessment however it was unclear whether some studies generated an eligible result.	Low risk: all studies included patients with depression receiving pro-dopaminergic interventions and assessed for anhedonia.	No clear indication of other biases

Source of Evidence (Human studies)	Outcome	Timepoint	Summary of the association	Bias due to study limitations	Reporting bias	Indirectness	Bias due to other reasons
Studies on pro-dopaminergic interventions vs placebo	Nausea reported for pro-dopaminergic interventions vs placebo	4-12 weeks	N=26, n=6489; OR=1.46, 95% CI: 1.21, 1.76, 95% PrI: 0.92, 2.29	Low risk: 73% of studies were rated low risk for RoB2 while 23% were rated moderate.	Moderate risk: The potential for missing studies across the review was deemed to be low in domain 3 of the RoB-ME assessment however it was unclear whether some studies generated an eligible result.	Low risk: all studies included patients with depression receiving pro-dopaminergic interventions and assessed for anhedonia.	No clear indication of other biases
Studies on pro-dopaminergic interventions vs placebo	Insomnia reported for pro-dopaminergic interventions vs placebo	4-12 weeks	N=24, n=6432; OR=1.81, 95% CI: 1.46, 2.25, 95% PrI: 1.18, 2.78	Low risk: 73% of studies were rated low risk for RoB2 while 23% were rated moderate.	Moderate risk: The potential for missing studies across the review was	Low risk: all studies included patients with depression receiving pro-dopaminergic interventions and	No clear indication of other biases.

Source of Evidence (Human studies)	Outcome	Timepoint	Summary of the association	Bias due to study limitations	Reporting bias	Indirectness	Bias due to other reasons
					deemed to be low in domain 3 of the RoB-ME assessment however it was unclear whether some studies generated an eligible result.	assessed for anhedonia.	
Studies on pro-dopaminergic interventions vs placebo	Vomiting reported for pro-dopaminergic interventions vs placebo	6-12 weeks	N=5, n=962; OR=1.90, 95%CI: 0.90, 4.00 95%PrI: 0.82, 4.42	Moderate risk: 4of 5 studies were rated as moderate risk and 1 study as high risk due to concerns in RoB2 domains 3 (missing outcome data) and 4 (measuring the outcome).	Moderate risk: The potential for missing studies across the review was deemed to be low in domain 3 of the RoB-ME assessment however it was unclear	Low risk: all studies included patients with depression receiving pro-dopaminergic interventions and assessed for anhedonia.	No clear indication of other biases.

Source of Evidence (Human studies)	Outcome	Timepoint	Summary of the association	Bias due to study limitations	Reporting bias	Indirectness	Bias due to other reasons
					whether some studies generated an eligible result.		

3. Abbreviations

- CI: Confidence Interval
- GALENOS: Global Alliance for Living Evidence on aNxiety depressiOn and pSychosis
- IPD: Individual Participant Data
- OR: Odds Ratio
- N: number of studies
- n: number of participants
- NI: No Information
- SD: Standard Deviation
- SMD: Standard Mean Difference
- REML: Restricted Maximum Likelihood
- RoB2: Risk of Bias 2
- ROB-ME: Risk of Bias for Missing Evidence

4. Software Used

We used R version 4.3.1 (R Core Team 2023) and the following packages; meta (Balduzzi, Rucker, and Schwarzer, 2019); dplyr (Wickham et al. 2023); readxl (Wickham and Bryan, 2023); kableExtra (Zhu, 2024).

5. References

- Higgins, J. P., Savović, J., Page, M. J., Elbers, R. G., & Sterne, J. A. (2019). Assessing risk of bias in a randomized trial. *Cochrane handbook for systematic reviews of interventions*, 205-228.
- Page, M. J., Sterne, J. A., Boutron, I., Hróbjartsson, A., Kirkham, J. J., Li, T., ... & Higgins, J. P. (2023). ROB-ME: a tool for assessing risk of bias due to missing evidence in systematic reviews with meta-analysis. *bmj*, *383*

6 Table of included studies

Author (Year)	Country	Sponsor	Treatment duration	Intervention group	Participants (n)	Females (n)	Age (mean)	Dosage fixed or flexible	Intervention format	Planned dosage range (min-max mg/day)	Delivered dosage range (min-max mg/day)	Delivered dosage (mean mg/day)
Agosti (1991)	USA	NA	6 weeks	Phenelzine 60-90mg	10	NA	NA	Flexible	Oral	60mg to 90mg	NA	NA
Agosti (1991)	USA	NA	6 weeks	Placebo	23	NA	NA	Flexible	Oral	NA	NA	NA
Agosti (1991)	USA	NA	6 weeks	Selegiline 40mg	12	NA	NA	Fixed	Oral	40mg	40mg	NA
Amsterdam (1989)	USA	Sanofi Research (partial grant)	4 weeks	Minaprine 100mg	34	12	41	Flexible	Oral	100mg to 400mg	NA	93mg
Amsterdam (1989)	USA	Sanofi Research (partial grant)	4 weeks	Minaprine 200mg	39	25	37	Flexible	Oral	100mg to 400mg	NA	186mg
Amsterdam (1989)	USA	Sanofi Research (partial grant)	4 weeks	Minaprine 300mg	43	22	40	Flexible	Oral	100mg to 400mg	NA	279mg

Author (Year)	Country	Sponsor	Treatment duration	Intervention group	Participants (n)	Females (n)	Age (mean)	Dosage fixed or flexible	Intervention format	Planned dosage range (min-max mg/day)	Delivered dosage range (min-max mg/day)	Delivered dosage (mean mg/day)
Amsterdam (1989)	USA	Sanofi Research (partial grant)	4 weeks	Minaprine 400mg	37	22	37	Flexible	Oral	100mg to 400mg	NA	352mg
Amsterdam (1989)	USA	Sanofi Research (partial grant)	4 weeks	Placebo	37	18	39	NA	Oral	NA	NA	NA
Amsterdam (2003)	USA	Somerset Pharmaceuticals, Inc.	8 weeks	Selegiline 20mg	149	94	41.2	Fixed	Transdermal	20mg	20mg	NA
Amsterdam (2003)	USA	Somerset Pharmaceuticals, Inc.	8 weeks	Placebo	152	99	43.5	Fixed	Transdermal	NA	NA	NA
Bakish (1992)	Canada	NA	6 weeks	Moclobemide up to 600mg	58	26	42	Flexible	Oral	NA to 600mg	NA to 600mg	492mg
Bakish (1992)	Canada	NA	6 weeks	Placebo	56	20	44	Flexible	Oral	NA	NA	NA
Bellak (1966)	USA	*MH	4 weeks	Placebo	25	NA	NA	NA	NA	NA	NA	NA
Bellak (1966)	USA	*MH	4 weeks	Phenelzine	25	NA	NA	NA	NA	NA	NA	NA

Author (Year)	Country	Sponsor	Treatment duration	Intervention group	Participants (n)	Females (n)	Age (mean)	Dosage fixed or flexible	Intervention format	Planned dosage range (min-max mg/day)	Delivered dosage range (min-max mg/day)	Delivered dosage (mean mg/day)
Benes (2011)	Germany	GSK	12 weeks	Placebo	67	45	59.5	Flexible to week 7, then fixed	Oral	NA	NA	NA
Benes (2011)	Germany	GSK	12 weeks	Ropinirole 0.5-4mg	198	144	58.2	Flexible to week 7, then fixed	Oral	0.5 mg to 4 mg	NA	1.9 mg
Bodkin (2002)	NA	Somerset Pharmaceuticals	6 weeks	Placebo	88	53	43.2	Fixed	Transdermal	NA	NA	NA
Bodkin (2002)	NA	Somerset Pharmaceuticals	6 weeks	Selegiline 20mg	89	53	41.4	Fixed	Transdermal	20mg	20mg	20mg
Botte (1992)	NA	NA	6 weeks	Placebo	24	16	43.3	NA	NA	NA	NA	NA

Author (Year)	Country	Sponsor	Treatment duration	Intervention group	Participants (n)	Females (n)	Age (mean)	Dosage fixed or flexible	Intervention format	Planned dosage range (min-max mg/day)	Delivered dosage range (min-max mg/day)	Delivered dosage (mean mg/day)
Botte (1992)	NA	NA	6 weeks	Moclobemide 300-600mg	23	13	51.4	Flexible	Oral	300mg to 600mg	200mg to 600mg	NA
Bymaster (2011)	Romania, Serbia, USA	DOV Pharmaceuticals, Euthymics Bioscience	6 weeks	Placebo	29	19	49.5	Fixed	Oral	NA	NA	NA
Bymaster (2011)	Romania, Serbia, USA	DOV Pharmaceuticals, Euthymics Bioscience	6 weeks	Amitifadine 50mg	34	25	48.2	Fixed	Oral	50mg	50mg	NA
Casacchia (1984)	Italy	NA	4 weeks	Moclobemide 150-450 mg	18	8	49.5	Flexible	Oral	NA	150mg to 450mg	297.2mg
Casacchia (1984)	Italy	NA	4 weeks	Placebo	16	11	49	Flexible	Oral	150 to 400mg	150mg to 400mg	212.5mg
Chouinard (1993)	Canada, UK	NA	6 weeks	Placebo	109	66	40.2	Fixed	Oral	NA	NA	NA

Author (Year)	Country	Sponsor	Treatment duration	Intervention group	Participants (n)	Females (n)	Age (mean)	Dosage fixed or flexible	Intervention format	Planned dosage range (min-max mg/day)	Delivered dosage range (min-max mg/day)	Delivered dosage (mean mg/day)
Chouinard (1993)	Canada, UK	NA	6 weeks	Brofaromine 75-100mg	111	57	41.2	Fixed	Oral	75mg to 150mg	NA	NA
Clayton (2006a)	USA	Glaxo Wellcome Inc.	8 weeks	Bupropion	141	85	36.5	Flexible	Oral	300mg to 450mg	300mg to 450mg	323mg
Clayton (2006a)	USA	Glaxo Wellcome Inc.	8 weeks	Placebo	141	88	35.1	Flexible	Oral	NA	NA	NA
Clayton (2006b)	USA	Glaxo Wellcome Inc.	8 weeks	Bupropion	138	76	37	Flexible	Oral	300mg to 450mg	300mg to 450mg	309mg
Clayton (2006b)	USA	Glaxo Wellcome Inc.	9 weeks	Placebo	137	76	37	Flexible	Oral	NA	NA	NA
Coleman (1999)	USA	Glaxo Wellcome Inc.	8 weeks	Placebo	124	73	38.5	Flexible	Oral	NA	NA	NA
Coleman (1999)	USA	Glaxo Wellcome Inc.	8 weeks	Bupropion SR 150-400mg	122	68	38.1	Flexible	Oral	150mg to 400mg	100mg to 365mg	290mg

Author (Year)	Country	Sponsor	Treatment duration	Intervention group	Participants (n)	Females (n)	Age (mean)	Dosage fixed or flexible	Intervention format	Planned dosage range (min-max mg/day)	Delivered dosage range (min-max mg/day)	Delivered dosage (mean mg/day)
Coleman (1999)	USA	Glaxo Wellcome Inc.	8 weeks	Placebo	152	92	36.7	Flexible	Oral	NA	NA	NA
Coleman (1999)	USA	Glaxo Wellcome Inc.	8 weeks	Bupropion 150-400mg	150	95	36.6	Flexible	Oral	150mg to 400mg	NA	NA
Corrigan (2000)	USA	NA	8 weeks	Placebo	35	NA	NA	Fixed	Oral	NA	NA	NA
Corrigan (2000)	USA	NA	8 weeks	Pramipexole 0.375mg	36	NA	NA	Fixed	Oral	0.375mg	0.375mg	0.375mg
Corrigan (2000)	USA	NA	8 weeks	Pramipexole 1mg	35	NA	NA	Fixed	Oral	1mg	1mg	1mg
Corrigan (2000)	USA	NA	8 weeks	Pramipexole 5mg	33	NA	NA	Fixed	Oral	5mg	5mg	5mg
Croft (1999)	NA	Glaxo Wellcome Inc.	8 weeks	Placebo	121	61	37.4	Flexible	Oral	NA	NA	NA

Author (Year)	Country	Sponsor	Treatment duration	Intervention group	Participants (n)	Females (n)	Age (mean)	Dosage fixed or flexible	Intervention format	Planned dosage range (min-max mg/day)	Delivered dosage range (min-max mg/day)	Delivered dosage (mean mg/day)
Croft (1999)	NA	Glaxo Wellcome Inc.	8 weeks	Bupropion SR 150-400mg	120	61	35.9	Flexible	Oral	150mg to 400mg	150mg to 400mg	293mg
Davidson (1988)	NA	Hoffmann La Roche	6 weeks	Isocarboxazid	68	37	41.9	Flexible	Oral	NA	NA	49.3mg
Davidson (1988)	NA	Hoffmann La Roche	6 weeks	Placebo	62	35	41.9	Flexible	Oral	NA	NA	NA
DelBello (2014)	USA	Somerset Pharmaceutical, Inc.	12 weeks	Placebo	156	104	14.7	Flexible	Transdermal	NA	NA	NA
DelBello (2014)	USA	Somerset Pharmaceutical, Inc.	12 weeks	Selegiline 6-12mg	152	93	14.8	Flexible	Transdermal	6mg to 12mg	NA	NA
Feiger (2006)	USA	Somerset Pharmaceutical, Inc.	8 weeks	Selegiline 6-12mg	132	81	42	Flexible	Transdermal	6mg to 12mg	6mg to 12mg	NA
Feiger (2006)	USA	Somerset Pharmaceutical, Inc.	8 weeks	Placebo	133	71	42	Flexible	Transdermal	NA	NA	NA

Author (Year)	Country	Sponsor	Treatment duration	Intervention group	Participants (n)	Females (n)	Age (mean)	Dosage fixed or flexible	Intervention format	Planned dosage range (min-max mg/day)	Delivered dosage range (min-max mg/day)	Delivered dosage (mean mg/day)
Feighner (1984)	NA	NA	4 weeks	Placebo	22	19	49	NA	Oral	NA	NA	NA
Feighner (1984)	NA	NA	4 weeks	Bupropion up to 600mg	44	30	43.9	Flexible	Oral	NA to 600mg	300mg to 600mg	392mg
Georgotas (1986)	USA	NAMH	7 weeks	Placebo	28	15	64.7	Flexible	Oral	NA	NA	NA
Georgotas (1986)	USA	NAMH	7 weeks	Phenelzine	22	13	65.5	Flexible	Oral	NA	NA	53.90mg
Giller (1982)	USA	NAMH, Hoffman-LaRoche	6 weeks	Placebo	NA	NA	NA	Flexible	Oral	NA	NA	73mg
Giller (1982)	USA	NAMH, Hoffman-LaRoche	6 weeks	Isocarboxazid	NA	NA	NA	Flexible	Oral	NA	NA	48mg
GlaxoSmithKline (1980)	USA	GSK	6 weeks	Bupropion 150-450mg	52	35	36.4	Flexible	Oral	150mg to 450mg	NA	NA

Author (Year)	Country	Sponsor	Treatment duration	Intervention group	Participants (n)	Females (n)	Age (mean)	Dosage fixed or flexible	Intervention format	Planned dosage range (min-max mg/day)	Delivered dosage range (min-max mg/day)	Delivered dosage (mean mg/day)
GlaxoSmithKline (1980)	USA	GSK	6 weeks	Bupropion 300-900mg	23	13	37.8	Flexible	NA	300mg to 900mg	NA	NA
GlaxoSmithKline (1980)	USA	GSK	6 weeks	Placebo	47	31	37.4	Flexible	Oral	NA	NA	NA
GlaxoSmithKline (1985)	USA, Canada	GSK	4 weeks	Placebo	43	20	51.9	Fixed	Oral	NA	NA	NA
GlaxoSmithKline (1985)	USA, Canada	GSK	4 weeks	Bupropion 300mg	45	18	52.4	Fixed	Oral	300mg	300mg	NA
GlaxoSmithKline (1985)	USA, Canada	GSK	4 weeks	Bupropion 450mg	40	18	47.5	Fixed	Oral	450mg	450mg	NA
GlaxoSmithKline (1993)	USA	GSK	8 weeks	Placebo	124	80	40.7	NA	NA	NA	NA	NA
GlaxoSmithKline (1993)	USA	GSK	8 weeks	Bupropion SR 100mg	119	77	39.6	Fixed	NA	100mg	NA	NA
GlaxoSmithKline (1993)	USA	GSK	8 weeks	Bupropion SR 200mg	120	65	39.6	Fixed	NA	200mg	NA	NA

Author (Year)	Country	Sponsor	Treatment duration	Intervention group	Participants (n)	Females (n)	Age (mean)	Dosage fixed or flexible	Intervention format	Planned dosage range (min-max mg/day)	Delivered dosage range (min-max mg/day)	Delivered dosage (mean mg/day)
GlaxoSmithKline (1993)	USA	GSK	8 weeks	Bupropion SR 300mg	120	70	39.9	Fixed	NA	300mg	NA	NA
GlaxoSmithKline (1993)	USA	GSK	8 weeks	Bupropion SR 400mg	119	66	38.8	Fixed	NA	400mg	NA	NA
GlaxoSmithKline (1994)	USA	GSK	8 weeks	Bupropion 50-150mg	152	90	39.1	Flexible	Oral	50mg to 150mg	50mg to 150mg	NA
GlaxoSmithKline (1994)	USA	GSK	8 weeks	Placebo	154	99	38.2	NA	Oral	NA	NA	NA
GlaxoSmithKline (1994)	USA	GSK	8 weeks	Bupropion 100-300mg	150	98	37.2	Flexible	Oral	100mg to 300mg	100mg to 300mg	NA
Han (2012)	South Korea	Korea Research Foundation Grant	8 weeks	Placebo	28	0	18.1	NA	NA	NA	NA	NA
Han (2012)	South Korea	Korea Research Foundation Grant	8 weeks	Bupropion 150-300mg	29	0	21.2	Fixed	Oral	150mg to 300mg	NA	NA

Author (Year)	Country	Sponsor	Treatment duration	Intervention group	Participants (n)	Females (n)	Age (mean)	Dosage fixed or flexible	Intervention format	Planned dosage range (min-max mg/day)	Delivered dosage range (min-max mg/day)	Delivered dosage (mean mg/day)
Hewett (2009)	Austria, Belgium, Bulgaria, Croatia, Estonia, Finland, Greece, Ireland, Latvia, Netherlands, Poland, Portugal, Russia, Slovakia, Spain, Sweden and Mexico	GSK	8 weeks	Placebo	199	142	41.8	Flexible	Oral	NA	NA	NA
Hewett (2009)	Austria, Belgium, Bulgaria, Croatia, Estonia, Finland,	GSK	8 weeks	Bupropion XR 150-300mg	188	138	41.8	Flexible	Oral	150mg to 300mg	NA	170.1mg

Author (Year)	Country	Sponsor	Treatment duration	Intervention group	Participants (n)	Females (n)	Age (mean)	Dosage fixed or flexible	Intervention format	Planned dosage range (min-max mg/day)	Delivered dosage range (min-max mg/day)	Delivered dosage (mean mg/day)
	Greece, Ireland, Latvia, Netherlands, Poland, Portugal, Russia, Slovakia, Spain, Sweden and Mexico											
Hewett (2010a)	Australia, France, Germany, the Netherlands, Norway, South Africa and Sweden	GSK	8 weeks	Placebo	189	125	44.5	Flexible	Oral	NA	NA	NA

Author (Year)	Country	Sponsor	Treatment duration	Intervention group	Participants (n)	Females (n)	Age (mean)	Dosage fixed or flexible	Intervention format	Planned dosage range (min-max mg/day)	Delivered dosage range (min-max mg/day)	Delivered dosage (mean mg/day)
Hewett (2010a)	Australia, France, Germany, the Netherlands, Norway, South Africa and Sweden	GSK	8 weeks	Bupropion XR 150-300mg	204	127	45.6	Flexible	Oral	150mg to 300mg	150mg to 300mg	180mg
Hewett (2010b)	Australia, Belgium, Canada, Croatia, Finland, France, Germany, India, Latvia, Netherlands, Norway, Poland, Republic of South	GSK	10 weeks	Placebo	207	144	71.3	Flexible	Oral	NA	NA	NA

Author (Year)	Country	Sponsor	Treatment duration	Intervention group	Participants (n)	Females (n)	Age (mean)	Dosage fixed or flexible	Intervention format	Planned dosage range (min-max mg/day)	Delivered dosage range (min-max mg/day)	Delivered dosage (mean mg/day)
	Africa, Russia and United States											
Hewett (2010b)	Australia, Belgium, Canada, Croatia, Finland, France, Germany, India, Latvia, Netherlands, Norway, Poland, Republic of South Africa, Russia and United States	GSK	10 weeks	Bupropion XR 150-300mg	211	157	70.9	Flexible	Oral	150mg to 300mg	150mg to 300mg	NA

Author (Year)	Country	Sponsor	Treatment duration	Intervention group	Participants (n)	Females (n)	Age (mean)	Dosage fixed or flexible	Intervention format	Planned dosage range (min-max mg/day)	Delivered dosage range (min-max mg/day)	Delivered dosage (mean mg/day)
Iosifescu (2022)	USA	Xsome Therapeutics	6 weeks	Dextromethorphan + Bupropion	156	95	42.1	NA	Oral	45mg to 105mg	45mg to 105mg	NA
Iosifescu (2022)	USA	Xsome Therapeutics	6 weeks	Placebo	162	117	41.2	NA	Oral	NA	NA	NA
Jarrett (1999)	NA	NA	10 weeks	Phenelzine 0.85-1mg/kg	36	25	38.7	NA	NA	0.85mg/kg to 1mg/kg	NA	64mg
Jarrett (1999)	NA	NA	10 weeks	Placebo	36	22	40.3	NA	NA	NA	NA	NA
Jefferson (2006)	NA	GSK	8 weeks	Placebo	139	96	39.8	Flexible	Oral	NA	NA	NA
Jefferson (2006)	NA	GSK	8 weeks	Bupropion XR 150-450mg	135	89	40	Flexible	Oral	150mg to 450mg	150mg to 450mg	NA
Koshino (2013)	Japan, South Korea	GSK	10 weeks	Placebo	186	85	37.9	Fixed	Oral	NA	NA	NA

Author (Year)	Country	Sponsor	Treatment duration	Intervention group	Participants (n)	Females (n)	Age (mean)	Dosage fixed or flexible	Intervention format	Planned dosage range (min-max mg/day)	Delivered dosage range (min-max mg/day)	Delivered dosage (mean mg/day)
Koshino (2013)	Japan, South Korea	GSK	10 weeks	Bupropion 150mg	190	92	36	Fixed	Oral	150mg	150mg	NA
Koshino (2013)	Japan, South Korea	GSK	10 weeks	Bupropion 300mg	188	83	37.5	Fixed	Oral	300mg	300mg	NA
Kusalic (1993)	NA	NA	6 weeks	Placebo	9	NA	NA	Flexible	Oral	NA	NA	NA
Kusalic (1993)	NA	NA	6 weeks	Moclobemide	11	NA	NA	Flexible	Oral	NA	NA	482.60 mg
Larsen (1989)	Denmark	NA	6 weeks	Placebo	18	12	57	Flexible	Oral	NA	NA	NA
Larsen (1989)	Denmark	NA	6 weeks	Moclobemide up to 300mg	22	15	51	Flexible	Oral	NA to 300mg	NA to 300mg	NA
Learned (2012a)	Australia, Belgium, Bulgaria, Canada, Estonia, Finland,	GSK	10 weeks	Placebo	126	46	41.9	Fixed	Oral	NA	NA	NA

Author (Year)	Country	Sponsor	Treatment duration	Intervention group	Participants (n)	Females (n)	Age (mean)	Dosage fixed or flexible	Intervention format	Planned dosage range (min-max mg/day)	Delivered dosage range (min-max mg/day)	Delivered dosage (mean mg/day)
	France, Germany, India, Poland, Slovakia, and South Africa											
Learned (2012a)	Australia, Belgium, Bulgaria, Canada, Estonia, Finland, France, Germany, India, Poland, Slovakia, and South Africa	GSK	10 weeks	GSK372475 1.5-2mg	134	51	43	Fixed	Oral	1mg to 2mg	1mg to 2mg	NA
Learned (2012b)	Bulgaria, Canada, Chile, Costa	GSK	10 weeks	Placebo	156	39	41.8	Fixed	Oral	NA	NA	NA

Author (Year)	Country	Sponsor	Treatment duration	Intervention group	Participants (n)	Females (n)	Age (mean)	Dosage fixed or flexible	Intervention format	Planned dosage range (min-max mg/day)	Delivered dosage range (min-max mg/day)	Delivered dosage (mean mg/day)
	Rica, Croatia, France, Germany, India, Italy, and Poland											
Learned (2012b)	Bulgaria, Canada, Chile, Costa Rica, Croatia, France, Germany, India, Italy, and Poland	GSK	10 weeks	GSK372475 1-1.5mg	171	54	42.4	Fixed	Oral	1mg to 1.5mg	1mg to 1.5mg	NA
Liebowitz (1984)	NA	Public Health Service	6 weeks	Placebo	24	14	37.7	Flexible	Oral	NA	NA	NA
Liebowitz (1984)	NA	Public Health Service	6 weeks	Phenelzine 15-90mg	15	7	33.8	Flexible	Oral	15mg to 90mg	60mg to 90mg	74mg

Author (Year)	Country	Sponsor	Treatment duration	Intervention group	Participants (n)	Females (n)	Age (mean)	Dosage fixed or flexible	Intervention format	Planned dosage range (min-max mg/day)	Delivered dosage range (min-max mg/day)	Delivered dosage (mean mg/day)
Mann (1989)	USA	Irma Hirschl and Mallinckrodt Foundations	6 weeks	Placebo	22	17	40.2	Flexible	NA	NA	NA	NA
Mann (1989)	USA	Irma Hirschl and Mallinckrodt Foundations	6 weeks	Selegiline up to 50mg	22	16	45.2	Flexible	Oral	NA	NA to 50mg	NA
Nair (1995)	Canada, Denmark, UK	NA	7 weeks	Moclobemide 400mg	36	25	67 (median)	Fixed	Oral	400mg	400mg	NA
Nair (1995)	Canada, Denmark, UK	NA	7 weeks	Placebo	35	25	71 (median)	Fixed	Oral	NA	NA	NA
Ose (1992)	NA	NA	4 weeks	Moclobemide 300-500mg	35	21	49 (median)	Fixed	Oral	300mg to 500mg	300mg to 500mg	NA
Ose (1992)	NA	NA	4 weeks	Placebo	33	18	50 (median)	Fixed	Oral	NA	NA	NA

Author (Year)	Country	Sponsor	Treatment duration	Intervention group	Participants (n)	Females (n)	Age (mean)	Dosage fixed or flexible	Intervention format	Planned dosage range (min-max mg/day)	Delivered dosage range (min-max mg/day)	Delivered dosage (mean mg/day)
Parnetti (1993)	Italy	Gruppo Sanofi	12 weeks	Minapramine 200mg	63	36	71.6	Fixed	Oral	200mg	200mg	200mg
Parnetti (1993)	Italy	Gruppo Sanofi	12 weeks	Placebo	67	47	71.3	Fixed	Oral	NA	NA	NA
Quitkin (1990)	USA	*MH; NHCRC	6 weeks	Phenelzine 90mg	33	NA	38.9	Fixed	Oral	90mg	90mg	NA
Quitkin (1990)	USA	*MH; NHCRC	6 weeks	Placebo	34	NA	30.1	Fixed	Oral	NA	NA	NA
Raft (1981)	USA	*H	5 weeks	Phenelzine 90mg	NA	NA	NA	Fixed	Oral	90mg	90mg	90mg
Raft (1981)	USA	*H	5 weeks	Placebo	NA	NA	NA	Fixed	Oral	NA	NA	NA
Rampello (1991)	Italy	NA	6 weeks	Minaprine 100mg	10	NA	NA	Fixed	Oral	100mg	100mh	NA
Rampello (1991)	Italy	NA	6 weeks	Placebo	10	NA	NA	Fixed	Oral	NA	NA	NA
Raskin (1972)	USA	*MH	5 weeks	Placebo	111	NA	NA	Fixed	Oral	NA	NA	NA

Author (Year)	Country	Sponsor	Treatment duration	Intervention group	Participants (n)	Females (n)	Age (mean)	Dosage fixed or flexible	Intervention format	Planned dosage range (min-max mg/day)	Delivered dosage range (min-max mg/day)	Delivered dosage (mean mg/day)
Raskin (1972)	USA	*MH	5 weeks	Phenelzine 45mg	110	72	37 (median)	Fixed	Oral	45mg	45mg	NA
Ravaris (1976)	NA	Public Health Service; Warner Lambert Research Institute	6 weeks	Phenelzine 60mg	21	NA	43.1	NA	Oral	60mg	60mg	NA
Ravaris (1976)	NA	Public Health Service; Warner Lambert Research Institute	6 weeks	Phenelzine 30mg	21	NA	41.2	Fixed	Oral	30mg	30mg	NA
Ravaris (1976)	NA	Public Health Service; Warner Lambert Research Institute	6 weeks	Placebo	21	NA	38.9	Fixed	Oral	NA	NA	NA

Author (Year)	Country	Sponsor	Treatment duration	Intervention group	Participants (n)	Females (n)	Age (mean)	Dosage fixed or flexible	Intervention format	Planned dosage range (min-max mg/day)	Delivered dosage range (min-max mg/day)	Delivered dosage (mean mg/day)
Reimherr (1998)	USA	Glaxo Wellcome Inc.	8 weeks	Placebo	121	69	40.2	Fixed	Oral	NA	NA	NA
Reimherr (1998)	USA	Glaxo Wellcome Inc.	8 weeks	Bupropion SR 150mg	121	86	38.3	Fixed	Oral	150mg	150mg	NA
Reimherr (1998)	USA	Glaxo Wellcome Inc.	8 weeks	Bupropion SR 300mg	120	92	38.6	Fixed	Oral	300mg	300mg	NA
Rickels (1970)	USA	Public Health Service	4 weeks	Methylphenidate 15mg	NA	NA	NA	Fixed	Oral	15mg	15mg	NA
Rickels (1970)	USA	Public Health Service	4 weeks	Placebo	NA	NA	NA	Fixed	Oral	NA	NA	NA
Riesenberg (2010)	USA	Rexahn Pharmaceuticals	8 weeks	Placebo	21	11	42.5	Fixed	Oral	NA	NA	NA
Riesenberg (2010)	USA	Rexahn Pharmaceuticals	8 weeks	RX-10100 5mg	21	11	44.8	Fixed	Oral	5mg	5mg	NA

Author (Year)	Country	Sponsor	Treatment duration	Intervention group	Participants (n)	Females (n)	Age (mean)	Dosage fixed or flexible	Intervention format	Planned dosage range (min-max mg/day)	Delivered dosage range (min-max mg/day)	Delivered dosage (mean mg/day)
Riesenberg (2010)	USA	Rexahn Pharmaceuticals	8 weeks	RX-10100 10mg	16	7	42.6	Fixed	Oral	10mg	10mg	NA
Riesenberg (2010)	USA	Rexahn Pharmaceuticals	8 weeks	RX-10100 15mg	17	9	39.4	Fixed	Oral	15mg	15mg	NA
Robin (1958)	UK	NA	4 weeks	Placebo	23	13	39.5	Flexible	Oral	NA	NA	NA
Robin (1958)	UK	NA	4 weeks	Methylphenidate 20-40mg	22	16	37.5	Flexible	Oral	20mg to 40mg	NA	NA
Rowan (1980)	NA	NA	6 weeks	Phenelzine 45-75mg	NA	NA	NA	Flexible	Oral	45mg to 75mg	NA	NA
Rowan (1980)	NA	NA	6 weeks	Placebo	NA	NA	NA	Fixed	Oral	NA	NA	NA
Tomarken (2004)	USA	Glaxo Wellcome Inc.	6 weeks	Bupropion SR 300-400mg	10	6	39.4	Fixed	Oral	300mg to 400mg	100mg to 400mg	NA

Author (Year)	Country	Sponsor	Treatment duration	Intervention group	Participants (n)	Females (n)	Age (mean)	Dosage fixed or flexible	Intervention format	Planned dosage range (min-max mg/day)	Delivered dosage range (min-max mg/day)	Delivered dosage (mean mg/day)
Tomarken (2004)	USA	Glaxo Wellcome Inc.	6 weeks	Placebo	9	6	37.5	Fixed	Oral	NA	NA	NA
Ucha (1990)	NA	NA	6 weeks	Placebo	24	13	42.2	Flexible	Oral	NA	NA	5.6 tabs per day
Ucha (1990)	NA	NA	6 weeks	Moclobemide 300-600mg	24	16	40.5	Flexible	Oral	300mg to 600mg	NA	405mg
UK Moclobemide Study Group (1994)	UK	NA	6 weeks	Placebo	54	NA	NA	Fixed	Oral	NA	NA	NA
UK Moclobemide Study Group (1994)	UK	NA	6 weeks	Moclobemide 450mg	56	NA	NA	Fixed	Oral	450mg	450mg	NA

Author (Year)	Country	Sponsor	Treatment duration	Intervention group	Participants (n)	Females (n)	Age (mean)	Dosage fixed or flexible	Intervention format	Planned dosage range (min-max mg/day)	Delivered dosage range (min-max mg/day)	Delivered dosage (mean mg/day)
Versiani (1989)	NA	NA	6 weeks	Moclobemide 300-600mg	164	124	44	Flexible	Oral	300mg to 600mg	NA	NA
Versiani (1989)	NA	NA	6 weeks	Placebo	162	123	42	Flexible	Oral	NA	NA	NA
Versiani (1990)	Brazil	NA	6 weeks	Moclobemide 600mg	25	NA	NA	NA	Oral	600mg	600mg	600mg
Versiani (1990)	Brazil	NA	6 weeks	Placebo	25	NA	NA	NA	Oral	NA	NA	NA
Versiani (1997)	NA	NA	6 weeks	Moclobemide 75-750mg	108	73	41	Flexible	Oral	75mg to 750mg	75mg to 750mg	633mg
Versiani (1997)	NA	NA	6 weeks	Placebo	104	71	40	Flexible	Oral	NA	NA	NA
White (1984)	USA	NA	4 weeks	Tranylcypromine 30-60mg	63	14	38	Flexible	Oral	30mg to 60mg	NA	44.4mg
White (1984)	USA	NA	4 weeks	Placebo	59	21	39	Flexible	Oral	NA	NA	NA

Author (Year)	Country	Sponsor	Treatment duration	Intervention group	Participants (n)	Females (n)	Age (mean)	Dosage fixed or flexible	Intervention format	Planned dosage range (min-max mg/day)	Delivered dosage range (min-max mg/day)	Delivered dosage (mean mg/day)
Zarate (2006)	NA	NA	8 weeks	Memantine 5-20mg	16	9	47.1	Flexible	Oral	5mg to 20mg	5mg to 20mg	19.4mg
Zarate (2006)	NA	NA	8 weeks	Placebo	16	7	46.1	Flexible	Oral	NA	NA	NA
Zisook (1985)	NA	NA	6 weeks	Isocarboxazid up to 80mg	NA	NA	NA	Flexible	Oral	NA to 80mg	NA	39.00mg
Zisook (1985)	NA	NA	6 weeks	Placebo	NA	NA	NA	NA	NA	NA	NA	NA

7. Risk of Bias - Missing Evidence Assessment

7.1 ROB-ME Matrix Step 1

**Meta-analysis**	**Population**	**Intervention**	**Comparator**	**Outcome**	**Eligible study designs:**	**Eligible outcome definitions:**	**Eligible methods of analysis:**
Meta-analysis 1	Depressed adults	Pro-dopaminergic intervention	Placebo	Anhedonia	RCTs	Anhedonia measured with any scale, up to 12 weeks post-randomisation	Any method of analyses
Meta-analysis 2	Depressed adults	Pro-dopaminergic intervention	Placebo	Anxiety	RCTs	Anxiety measured with any scale, up to 12 weeks post-randomisation	Any method of analyses
Meta-analysis 3	Depressed adults	Pro-dopaminergic intervention	Placebo	Acceptability	RCTs	Acceptability was measured by any dropouts occuring at any point in the treatment phase following randomisation	Number of participants reporting event
Meta-analysis 4	Depressed adults	Pro-dopaminergic intervention	Placebo	Constipation	RCTs	Constipation was measured by any event of this occuring during the	Number of participants reporting event

Meta-analysis	Population	Intervention	Comparator	Outcome	Eligible study designs:	Eligible outcome definitions:	Eligible methods of analysis:
						treatment phase following randomisation	
Meta-analysis 5	Depressed adults	Pro-dopaminergic intervention	Placebo	Dizziness	RCTs	Dizziness was measured by any event of this occuring during the treatment phase following randomisation	Number of participants reporting event
Meta-analysis 6	Depressed adults	Pro-dopaminergic intervention	Placebo	Dry mouth	RCTs	Dry mouth was measured by any event of this occurring during the treatment phase following randomisation	Number of participants reporting event
Meta-analysis 7	Depressed adults	Pro-dopaminergic intervention	Placebo	Headache	RCTs	Headaches was measured by any event of this occurring during the treatment phase following randomisation	Number of participants reporting event
Meta-analysis 8	Depressed adults	Pro-dopaminergic intervention	Placebo	Insomnia	RCTs	Insomnia was measured by any event of this occurring during the treatment phase following randomisation	Number of participants reporting event

Meta-analysis	Population	Intervention	Comparator	Outcome	Eligible study designs:	Eligible outcome definitions:	Eligible methods of analysis:
Meta-analysis 9	Depressed adults	Pro-dopaminergic intervention	Placebo	Nausea	RCTs	Nausea was measured by any event of this occurring during the treatment phase following randomisation	Number of participants reporting event
Meta-analysis 10	Depressed adults	Pro-dopaminergic intervention	Placebo	Tolerability	RCTs	Tolerability was measured by any dropouts due to adverse events occurring at any point in the treatment phase following randomisation	Number of participants reporting event
Meta-analysis 11	Depressed adults	Pro-dopaminergic intervention	Placebo	Vomiting	RCTs	Vomiting was measured by any event of this occurring during the treatment phase following randomisation	Number of participants reporting event

7.2 ROB-ME Matrix Step 2

1 = A study result is available for inclusion in the meta-analysis, 2 = no study result is available for inclusion in the meta-analysis, for a reason unrelated to the P value, magnitude or direction of the result, 3 = unclear whether an eligible study result was generated, 4 = no study result is available for inclusion in the meta-analysis, likely because of the P value, magnitude or direction of the result generated, . = study did not measure outcome based on information provided in the publication or clinical trial report.

Study ID	Sources used	Anhedonia (MA1)	Anxiety (MA2)	Acceptability (MA3)	Constipation (MA4)	Dizziness (MA5)	Dry Mouth (MA6)	Headache (MA7)	Insomnia (MA8)	Nausea (MA9)	Tolerability (MA10)	Vomiting (MA11)
UK Moclobemide Study Group (1994)	Publication	.	.	3	1	3	1	3	3	3	1	3
Agosti (1991)	Publication	.	.	3	3	3	3	3	3	3	3	3
Amsterdam (1989)	Publication	.	.	1	3	3	3	3	3	3	3	3
Amsterdam (2003)	Publication	.	.	1	3	1	1	1	3	1	1	3
Bakish (1992)	Publication	.	.	1	3	3	3	3	3	3	1	3
Bellak (1966)	Publication	.	.	1	3	3	3	3	3	3	3	3
Benes (2011)	Publication	.	.	1	3	1	3	1	3	1	1	1
Bodkin (2002)	Publication	.	.	1	3	1	1	1	1	3	1	3
Botte (1992)	Publication	.	.	1	3	3	3	3	3	3	1	3

Study ID	Sources used	Anhedonia (MA1)	Anxiety (MA2)	Acceptability (MA3)	Constipation (MA4)	Dizziness (MA5)	Dry Mouth (MA6)	Headache (MA7)	Insomnia (MA8)	Nausea (MA9)	Tolerability (MA10)	Vomiting (MA11)
Bymaster (2011)	Conference abstract, publication	1	.	1	3	3	3	1	1	1	1	3
Casacchia (1984)	Publication	.	1	1	3	3	3	1	3	3	3	.
Chouinard (1993)	Publication	.	1	1	1	1	1	1	1	1	1	3
Clayton (2006a)	Publication, clinical result summary, clinical trial report	.	.	1	1	1	1	1	1	1	1	3
Clayton (2006b)	Publication, clinical result summary, clinical trial report	.	.	1	1	1	1	1	1	1	1	3
Coleman (1999)	Publication	.	.	1	3	3	1	1	3	1	1	3
Coleman (2001)	Publication, clinical trial report	.	.	1	3	3	1	1	1	1	3	3

Study ID	Sources used	Anhedonia (MA1)	Anxiety (MA2)	Acceptability (MA3)	Constipation (MA4)	Dizziness (MA5)	Dry Mouth (MA6)	Headache (MA7)	Insomnia (MA8)	Nausea (MA9)	Tolerability (MA10)	Vomiting (MA11)
Corrigan (2000)	Publication, clinical trial report	.	.	1	3	1	3	1	1	1	1	1
Croft (1999)	Publication, clinical trial report	.	.	1	1	3	1	1	3	1	1	3
Davidson (1988)	Publication	.	1	1	3	3	3	3	3	3	1	3
DelBello (2014)	Publication	.	.	1	3	1	3	1	1	1	1	1
Feiger (2006)	Publication	.	.	1	3	1	1	3	1	3	1	3
Feighner (1984)	Publication, clinical trial report	.	.	1	3	3	3	3	3	3	1	3
Georgotas (1986)	Publication	.	.	1	1	1	1	3	3	3	1	3
Giller (1982)	Publication	.	1	3	3	3	3	3	3	3	3	3
GlaxoSmithKline (1980)	Clinical trial report	.	1	1	1	1	1	1	1	1	1	1
GlaxoSmithKline (1985)	Clinical trial report	.	NA	1	1	3	1	1	1	1	1	1

Study ID	Sources used	Anhedonia (MA1)	Anxiety (MA2)	Acceptability (MA3)	Constipation (MA4)	Dizziness (MA5)	Dry Mouth (MA6)	Headache (MA7)	Insomnia (MA8)	Nausea (MA9)	Tolerability (MA10)	Vomiting (MA11)
GlaxoSmithKline (1993)	Clinical trial report	.	1	1	1	1	1	1	1	1	1	3
GlaxoSmithKline (1994)	Clinical trial report	.	1	1	1	1	1	1	1	1	1	3
Han (2012)	Publication	.	.	1	3	3	3	3	3	3	1	3
Hewett (2009)	Publication, clinical trial report	1	1	1	1	1	1	1	1	1	1	3
Hewett (2010a)	Publication, clinical result summary, clinical trial report	1	1	1	1	1	1	1	1	1	1	3
Hewett (2010b)	Publication, clinical trial report	1	1	1	1	1	1	1	1	1	1	3
Iosifescu (2022)	Conference abstract, publication	.	.	1	3	1	1	1	1	1	1	3
Jarrett (1999)	Conference abstract,	.	.	1	3	3	3	3	3	3	1	3

Study ID	Sources used	Anhedonia (MA1)	Anxiety (MA2)	Acceptability (MA3)	Constipation (MA4)	Dizziness (MA5)	Dry Mouth (MA6)	Headache (MA7)	Insomnia (MA8)	Nausea (MA9)	Tolerability (MA10)	Vomiting (MA11)
	publication, poster											
Jefferson (2006)	Publication, clinical trial report	1	1	1	1	1	1	1	1	1	1	3
Koshino (2013)	Publication, clinical trial report	1	.	1	1	1	1	1	1	1	1	1
Kusalic (1993)	Publication	.	.	3	3	3	3	3	3	3	3	3
Larsen (1989)	Publication	.	.	1	3	1	3	3	3	3	1	3
Learned (2012a)	Publication, clinical result summary	.	.	1	1	1	1	1	1	1	1	3
Learned (2012b)	Publication, clinical result summary	.	.	1	1	1	1	1	1	1	1	3
Liebowitz (1984)	Publication	.	1	1	3	3	3	3	3	3	3	3
Mann (1989)	Publication	.	.	1	1	1	1	1	1	1	3	3

Study ID	Sources used	Anhedonia (MA1)	Anxiety (MA2)	Acceptability (MA3)	Constipation (MA4)	Dizziness (MA5)	Dry Mouth (MA6)	Headache (MA7)	Insomnia (MA8)	Nausea (MA9)	Tolerability (MA10)	Vomiting (MA11)
Nair (1995)	Publication	.	.	3	3	3	3	3	3	3	1	3
Ose (1992)	Publication	.	.	1	3	3	3	1	1	3	1	3
Parnetti (1993)	Publication	.	1	1	3	3	1	3	3	1	.	3
Quitkin (1990)	Publication	.	1	1	3	3	3	3	3	3	1	3
Raft (1981)	Publication	.	.	1	3	1	3	3	3	3	3	3
Rampello (1991)	Publication	.	.	1	3	3	3	3	3	3	1	3
Raskin (1972)	Publication	.	.	3	3	3	3	3	3	3	3	3
Ravaris (1976)	Publication	.	1	1	3	3	3	3	3	3	3	3
Reimherr (1988)	Publication	.	1	1	1	1	1	1	1	1	1	3
Rickels (1970)	Publication	.	.	3	3	3	3	3	3	3	3	3
Riesenberg (2010)	Conference abstract, publication	.	.	1	1	3	3	3	3	3	3	3
Robin (1958)	Publication	.	.	1	3	3	3	3	3	3	1	3
Rowan (1980)	Publication	.	.	3	3	3	3	3	3	3	3	3

Study ID	Sources used	Anhedonia (MA1)	Anxiety (MA2)	Acceptability (MA3)	Constipation (MA4)	Dizziness (MA5)	Dry Mouth (MA6)	Headache (MA7)	Insomnia (MA8)	Nausea (MA9)	Tolerability (MA10)	Vomiting (MA11)
Tomarken (2004)	Publication	.	.	1	3	3	3	3	3	3	3	3
Ucha (1990)	Publication	.	.	3	3	3	3	3	3	3	3	3
Versiani (1989)	Publication	.	.	1	3	3	3	3	3	3	3	3
Versiani (1990)	Publication	.	.	1	3	3	3	3	3	3	1	3
Versiani (1997)	Publication	.	.	3	1	1	1	1	1	1	3	3
White (1984)	Publication	.	.	1	1	1	1	1	1	3	3	3
Zarate (2006)	Publication	.	.	1	3	3	3	3	3	3	1	3
Zisook (1985)	Publication	.	.	3	3	3	3	3	3	3	3	3

7.3 ROB-ME Step 3 & 4

Scores indicate potential for missing studies in the review (3) and risk of bias due to missing evidence in the meta-analysis, as per the ROB-ME tool, abbreviations indicate answers to signalling questions in the tool algorithm N = no, PN = probably no, PY = probably yes, Y = yes, NA = question not applicable.

Outcome	Specify meta-analysis	Specify meta-analysis result	Specify N	Specify n	3.1	3.2	3.3	4.1	4.2	4.3	4.4	4.5	4.6	4.7	4.8	Overall Risk of Bias Judgement
Anhedonia	Random effects meta-analysis of the effect of dopamine agonists on symptoms of anhedonia at 4-12 weeks	SMD -0.24 95%CI -0.46, -0.03	6	2079	N	PY	PY	N	NA	N	PY	N	NA	NA	NA	L
Anxiety	Random effects meta-analysis of the effect of dopamine agonists on symptoms of anxiety at 4-12 weeks	SMD -0.17 95%CI -0.24, -0.09	16	3517	N	PY	PY	N	NA	N	PY	N	NA	NA	NA	L
Acceptability	Random effects meta-analysis of the effect of	OR 0.97 95%CI 0.79, 1.17	52	9725	N	PY	PY	N	NA	Y	PN	N	NA	PN	NA	L

Outcome	Specify meta-analysis	Specify meta-analysis result	Specify N	Specify n	3.1	3.2	3.3	4.1	4.2	4.3	4.4	4.5	4.6	4.7	4.8	Overall Risk of Bias Judgement
	dropouts due to any reason															
Constipation	Random effects meta-analysis of reported constipation	OR 1.46 95%CI 1.17, 1.82	21	5375	N	PY	PY	N	NA	Y	PY	N	NA	PN	NA	SC
Dizziness	Random effects meta-analysis of reported dizziness	OR 1.70 95%CI 1.32, 2.18	24	6026	N	PY	PY	N	NA	Y	PY	N	NA	PN	NA	SC
Dry mouth	Random effects meta-analysis of reported dry mouth	OR 2.13 95%CI 1.73, 2.63	26	6895	N	PY	PY	N	NA	Y	PY	N	NA	PN	NA	SC
Headache	Random effects meta-analysis of	OR 1.14 95%CI 1.02, 1.28	28	7084	N	PY	PY	N	NA	Y	PY	N	NA	PN	NA	SC

Outcome	Specify meta-analysis	Specify meta-analysis result	Specify N	Specify n	3.1	3.2	3.3	4.1	4.2	4.3	4.4	4.5	4.6	4.7	4.8	Overall Risk of Bias Judgement
	reported headache															
Insomnia	Random effects meta-analysis of reported insomina	OR 1.81 95%CI 1.46, 2.25	24	6432	N	PY	PY	N	NA	Y	PY	N	NA	PN	NA	SC
Nausea	Random effects meta-analysis of reported nausea	OR 1.46 95%CI 1.21, 1.76	26	7046	N	PY	PY	N	NA	Y	PY	N	NA	PN	NA	SC
Tolerability	Random effects meta-analysis of dropouts due to adverse events	OR 1.83 95%CI 1.38, 2.41	43	9030	N	PY	PY	N	NA	Y	PY	N	NA	PN	NA	SC
Vomiting	Random effects meta-analysis of	OR 1.90 95%CI	5	962	N	PY	PY	N	NA	Y	PY	N	NA	PN	NA	SC

Outcome	Specify meta-analysis	Specify meta-analysis result	Specify N	Specify n	3.1	3.2	3.3	4.1	4.2	4.3	4.4	4.5	4.6	4.7	4.8	Overall Risk of Bias Judgement
	reported vomiting	1.13, 3.19														

Non-human animal studies

1. Flow of study selection and descriptives

The flow of study selection is shown in Figure 1. Studies included were published between 1992 and 2023. Overall, this analysis includes 27 studies containing 150 comparisons.

Table 1 below gives a summary of the included studies for the effect of model induction. N represents an aggregate of animals contributing to outcomes reported from control and treatment groups, and if the same control group has contributed to more than one experiment, those animals will be counted more than once.

Study	Model	Strain	Outcome	N
AMIRI, 2016	maternal separation	NMRI	Sucrose preference test	24
BORGES, 2013	iuGC	Wistar (rat)	DOPAC concentration	20
~	~	~	Dopamine concentration	20
~	~	~	Sucrose preference test	14
BROCCO, 2006	CMS	Wistar (rat)	Sucrose preference test	48
CARRATALA, 2023	Tetrabenzine	CD-1 (mouse)	Sucrose preference test	132
EREN, 2007	CMS	Wistar (rat)	Sucrose preference test	16
EREN, 2014	CUMS	Wistar (rat)	Sucrose preference test	16
FATIMA, 2020	PNS	Wistar (rat)	Dopamine receptor biology	24
~	~	~	Sucrose preference test	12
JIANG, 2014	Defeat	C57BL/6J (mouse)	Sucrose preference test	100

KOO, 2018	MCAO	C57BL/6 (mouse)	Sucrose preference test	36
KRUPINA, 1995	MPTP	Wistar (rat)	Sucrose preference test	12
MUSCAT, 1992	CMS	Lister hooded (rat)	Sucrose preference test	20
OSACKA, 2022	CMS	Sprague-dawley (rat)	Sucrose preference test	21
PAPP, 1993	CUMS	Lister hooded (rat)	Sucrose preference test	32
PAPP, 1996	CUMS	Wistar (rat)	Sucrose preference test	20
~	CUMS 2	~	~	16
QIAO, 2020	CUS	Sprague-dawley (rat)	Dopamine concentration	24
~	~	~	Dopamine receptor biology	24
~	~	~	Sucrose preference test	24
RANA, 2014	CMS	Swiss albino	Sucrose preference test	12
TAN, 2015	TBI	Sprague-dawley (rat)	DOPAC concentration	15
~	~	~	Dopamine concentration	15
~	~	~	Sucrose preference test	15
TOMAZ, 2020	LPS	Wistar (rat)	Sucrose preference test	16
WANG, 2021	CUS	C57BL/6 (mouse)	Sucrose preference test	16

WEI, 2021	CMS	C57BL/6J (mouse)	Sucrose preference test	12
WILLNER, 1994	CUMS	PVG Hooded	Sucrose preference test	44
WU, 2014	CUMS	Sprague-dawley (rat)	Sucrose preference test	12
YAN, 2022	CUMS	Sprague-dawley (rat)	DA/DOPC ratio	60
~	~	~	Dopamine concentration	60
~	~	~	Sucrose preference test	20
YU, 2016	CUS	Wistar (rat)	Sucrose preference test	12
YUAN, 2011	CUMS	Sprague-dawley (rat)	Sucrose preference test	17
ZHAO, 2018	CMS	Sprague-dawley (rat)	Sucrose preference test	20

Abbreviations

iuGC - intra-uterine glucocorticoid: CMS - chronic mild stress: CUMS - chronic unpredictable mild stress: CUS - chronic unpredicatble stress: PNS - prenatal stress: Defeat - social defeat stress: TBI - traumatic brain injury: LPS - intraperitoneal lipopolysaccharide: MCAO - middle cerebral artery occlusion: WAG/Rij = genetic model of absence epilepsy with co-morbid depression

Table 2 below gives a summary of the included studies for the effect of dopaminergic interventions. N represents an aggregate of animals contributing to outcomes reported from control and treatment groups, and if the same control group has contributed to more than one experiment, it will be counted twice.While some authors considered imipramine to have dopaminergic effects, we considered, given that this was a minor contribution to its pharmacological repertoire, that these studies should not be included in this iteration of the review.

Study	Model	Strain	Comparison	Outcome	N
AMIRI, 2016	maternal separation	NMRI	selegiline, 1 mg/kg	Sucrose preference test	12
~	~	~	selegiline, 3 mg/kg	Sucrose preference test	24
~	~	~	selegiline, 5 mg/kg	Sucrose preference test	12
BORGES, 2013	iuGC	Wistar (rat)	L-DOPA, 24 mg/kg	DOPAC concentration	20
~	~	~	L-DOPA, 24 mg/kg	Dopamine concentration	20
~	~	~	L-DOPA, 24 mg/kg	Sucrose preference test	14
BROCCO, 2006	CMS	Wistar (rat)	piribedil, 2.5 mg/kg	Sucrose preference test	16
~	~	~	piribedil, 10 mg/kg	Sucrose preference test	16
~	~	~	piribedil, 40 mg/kg	Sucrose preference test	16
CARRATALA, 2023	Tetrabenzine	CD-1 (mouse)	buproprion, 10 mg/kg	Sucrose preference test	132

EREN, 2007	CMS	Wistar (rat)	aripiprazole, 2.5 mg/kg	Sucrose preference test	16
EREN, 2014	CUMS	Wistar (rat)	aripiprazole, 2.5 mg/kg	Sucrose preference test	16
FATIMA, 2020	PNS	Wistar (rat)	ropinirole, 10 mg/kg	Dopamine receptor biology	24
~	~	~	ropinirole, 10 mg/kg	Sucrose preference test	12
JIANG, 2014	Defeat	C57BL/6J (mouse)	SKF83959, 0.5 mg/kg	Sucrose preference test	20
~	~	~	SKF83959, 1 mg/kg	Sucrose preference test	100
KOO, 2018	MCAO	C57BL/6 (mouse)	aripiprazole, 1 mg/kg	Sucrose preference test	36
KRUPINA, 1995	MPTP	Wistar (rat)	bromocriptine, 5 mg/kg	Sucrose preference test	12
MUSCAT, 1992	CMS	Lister hooded (rat)	quinpirole, 200 ug/kg	Sucrose preference test	20
OSACKA, 2022	CMS	Sprague-dawley (rat)	aripiprazole, 10 mg/kg	Sucrose preference test	22

PAPP, 1993	CUMS	Lister hooded (rat)	quinpirole, 100 ug/kg	Sucrose preference test	24
~	~	~	quinpirole, 200 ug/kg	Sucrose preference test	24
~	~	~	quinpirole, 400 ug/kg	Sucrose preference test	24
PAPP, 1996	CUMS	Wistar (rat)	D-amphetamine, 0.5 mg/kg	Sucrose preference test	20
~	CUMS 2	Wistar (rat)	D-amphetamine, 1.5 mg/kg	Sucrose preference test	16
QIAO, 2020	CUS	Sprague-dawley (rat)	dopamine, 3.83 ug	Sucrose preference test	24
~	~	~	quinpirole, 0.877 ug	Sucrose preference test	24
RANA, 2014	CMS	Swiss albino	bromocriptine, 2 mg/kg & simvastatin, 10 mg/kg	Sucrose preference test	12
~	~	~	L-DOPA, 200 mg/kg & simvastatin, 10 mg/kg	Sucrose preference test	12
~	~	~	simvastatin, 10 mg/kg	Sucrose preference test	12

RUSSO, 2013	WAG/Rij rat	WAG/Rij	aripiprazole, 0.3 mg/kg	Sucrose preference test	20
~	~	~	aripiprazole, 1 mg/kg	Sucrose preference test	20
~	~	~	aripiprazole, 3 mg/kg	Sucrose preference test	20
TAN, 2015	TBI	Sprague-dawley (rat)	amantadine, 45 mg/kg	DOPAC concentration	14
~	~	~	amantadine, 45 mg/kg	Dopamine concentration	14
~	~	~	amantadine, 45 mg/kg	Sucrose preference test	14
~	~	~	amantadine, 135 mg/kg	DOPAC concentration	14
~	~	~	amantadine, 135 mg/kg	Dopamine concentration	14
~	~	~	amantadine, 135 mg/kg	Sucrose preference test	14
TOMAZ, 2020	LPS	Wistar (rat)	tranylcypromine, 10 mg/kg	Sucrose preference test	16
WANG, 2021	CUS	C57BL/6 (mouse)	cryptotanshinone, 20 mg/kg	Sucrose preference test	16

WEI, 2021	CMS	C57BL/6J (mouse)	pramipexole, 1 mg/kg	Sucrose preference test	12
WILLNER, 1994	CUMS	PVG Hooded	pramipexole, 1 mg/kg	Sucrose preference test	88
~	~	PVG Hooded	pramipexole, 2 mg/kg	Sucrose preference test	88
WU, 2014	CUMS	Sprague-dawley (rat)	SKF38393, 1.12 ug	Sucrose preference test	12
YAN, 2022	CUMS	Sprague-dawley (rat)	P. orientalis seed, 10 mg/kg	DA/DOPC ratio	60
~	~	~	P. orientalis seed, 10 mg/kg	Dopamine concentration	60
~	~	~	P. orientalis seed, 10 mg/kg	Sucrose preference test	20
~	~	~	P. orientalis seed, 33 mg/kg	DA/DOPC ratio	60
~	~	~	P. orientalis seed, 33 mg/kg	Dopamine concentration	60
~	~	~	P. orientalis seed, 33 mg/kg	Sucrose preference test	20
~	~	~	P. orientalis seed, 100 mg/kg	DA/DOPC ratio	60

~	~	~	P. orientalis seed, 100 mg/kg	Dopamine concentration	60
~	~	~	P. orientalis seed, 100 mg/kg	Sucrose preference test	20
YU, 2016	CUS	Wistar (rat)	amantadine, 25 mg/kg	Sucrose preference test	12
YUAN, 2011	CUMS	Sprague-dawley (rat)	SKF38393, 1.12 ug/kg	Sucrose preference test	16
ZHAO, 2018	CMS	Sprague-dawley (rat)	2_HBC, 2.5 mg/kg	Sucrose preference test	20
~	~	~	2_HBC, 10 mg/kg	Sucrose preference test	20
~	~	~	buproprion, 2.5 mg/kg	Sucrose preference test	20

References of included studies are located in the appendix. Included studies used 28 unique disease model induction procedures.

1.1 Description of experiment types and methodological approach

Within the literature we identified distinct categories of experiments and the data presented would allow several meta-analytic contrasts to be drawn:

Effects of disease modelling. These are experiments investigating the effect of models of depression, reported in 58 experiments from 26 publications.

In these studies the:

- **Control group** is a group of animals that is (1) not subjected to a depression model induction paradigm and (2) is administered a control treatment (vehicle) or no treatment.
- **Intervention group** is a group of animals that is (1) subjected to a depression model induction paradigm and (2) is administered a control treatment (vehicle) or no treatment.

Treatment vs control. These were experiments investigating the effect of administering a dopaminergic agent, reported in 92 experiments from 27 publications.

In these studies the:

- **Control group** is a group of animals that is (1) subjected to a depression model induction paradigm and (2) administered a control treatment (vehicle) or no treatment.
- **Intervention group** is a group of animals that is (1) subjected to a depression model induction paradigm and (2) administered a TAAR1 agonist treatment.
- **Sham group** is a group of animals that is (1) not subjected to a depression model induction paradigm and (2) administered a control treatment (vehicle) or no treatment. These data are required to allow a 'normalised mean difference' effect size to be calculated, given by

$\mu^{-}C-\mu^{-}T\mu^{-}C-\mu^{-}S\times100\mu^{-}\mathrm{C}-\mu^{-}\mathrm{T}\mu^{-}\mathrm{C}-\mu^{-}\mathrm{S}\times100$

where $\mu^{-}C\mu^{-}\mathrm{C}$, $\mu^{-}T\mu^{-}\mathrm{T}$, $\mu^{-}S\mu^{-}\mathrm{S}$ are the mean reported scores in the control, treatment, and sham groups respectively.
Outcomes with ≥2 independent effect sizes were considered for meta-analysis. In this iteration of the review, this includes sucrose preference test, dopamine concentration, dopac concentration and dopamine receptor biology.

All analyses were conducted allowing for the following hierarchical levels in a random effects model, which accounts for features common to experimental contrasts such as a shared control group:

- **Level 1: Rodent strain** - effect sizes measured across experiments using the same rodent strain.

- **Level 2: Study** - effect sizes measured from different experiments presented in the same publication.
- **Level 3: Experiment** - effect sizes measured in the same experiment within a study, where often a control group contributes to several effect sizes.

Each level for the hierarchy was only included in the model if more than 4 categories were present for at least one of these levels. Where more than 4 categories are not present for all levels, the variance attributable to that level is reported as zero.

The hierarchical grouping may therefore be considered thus: **Strains** of laboratory animals are included in several **Studies**, each of which can report one or more **Experiments**, and each Experiment is comprised of at least two **Cohorts** which are considered identical except for differing in the experimental manipulation (the **Intervention**) or not being exposed to the disease modelling procedures (a **Sham** cohort, these only being used to provide a baseline for outcome measures to allow Normalised Mean Difference meta-analysis). An **Experiment** can include several **experimental contrasts**, for instance where different doses of drugs are compared to the same control group.

We constructed multilevel models without Knapp-Hartung adjustments as these are not available for rma.mv class objects in the metafor package. Instead, the model is set to test = "t" to use t- and F-distributions for making inferences, and dfs="contain" to improve the method of approximating degrees of freedom of these distributions.

The scales and units used to measure outcomes in preclinical studies often differ between studies although they may measure the same underlying biological construct. The primary effect size used for meta-analysis of preclinical studies is therefore the standardised mean difference (SMD, Hedge’s g). For experiments testing the effects of interventions we also present a sensitivity analysis using normalised mean difference (NMD), where there are sufficient data for sham procedures to allow this. This analysis is not possible for studies of the effect of modelling depression.

For some experimental contrasts, more than one outcome of the same category - for instance dopamine concentrations in different brain regions - was measured in the same cohort of animals. Sometimes, sucrose preference tests were performed in the same cohort at different times. Some publications used the same drug doses with the same outcome measures in independent experiments. For these reasons, some of the forest plots may appear to include ‘duplicate’ Study - Drug - Dose combinations with different outcomes. For the later, these are accounted for in the hierarchical analysis, but for the former there were insufficient levels of the different outcome category measured to allow for hierarchical analysis and so this was not performed.

2 Dopaminergic agent v Control

These experiments test the effect of dopaminergic agents on outcome in animals which have been exposed to a ‘modelling intervention’ intended to recapitulate some features of human depression. Modelling interventions comprise behavioural (47 experiments), genetic (3), pharmacological (9) and surgical (5) approaches. Outcomes include ‘apical’

endpoints (sucrose preference test), and also endpoints which might be considered intermediate, and may give insights to the mechanisms through which apical effects occur. These include concentrations of dopamine and DOPAC (an active metabolite of dopamine) and the ratio between these; and observed changes in dopamine receptor biology.

27 studies (92 comparisons) investigated the effects of dopaminergic agents versus Control. The number of studies and individual effect sizes for each outcome were:

- **Sucrose preference*:** 27 studies and 64 comparisons in 10 strains
- Dopamine concentration: 3 studies and 13 comparisons in 2 strains
- DOPAC concentration: 2 studies and 4 comparisons in 2 strains
- DA/DOPC ratio: 1 studies and 9 comparisons in 1 strain
- Dopamine receptor biology: 1 studies and 2 comparisons in 1 strain

* This outcomes was identified in the study protocol as primary outcomes of interest.

2.1 Outcome 1: Sucrose preference

2.1.1 Risk of bias

Figure 2.1.1 shows the risk of bias traffic light plot for studies investigating the effect of administering a dopaminergic agent on Sucrose preference in animals. The risk of bias assessment was performed using the SyRCLE RoB tool.

Figure 2.1.1

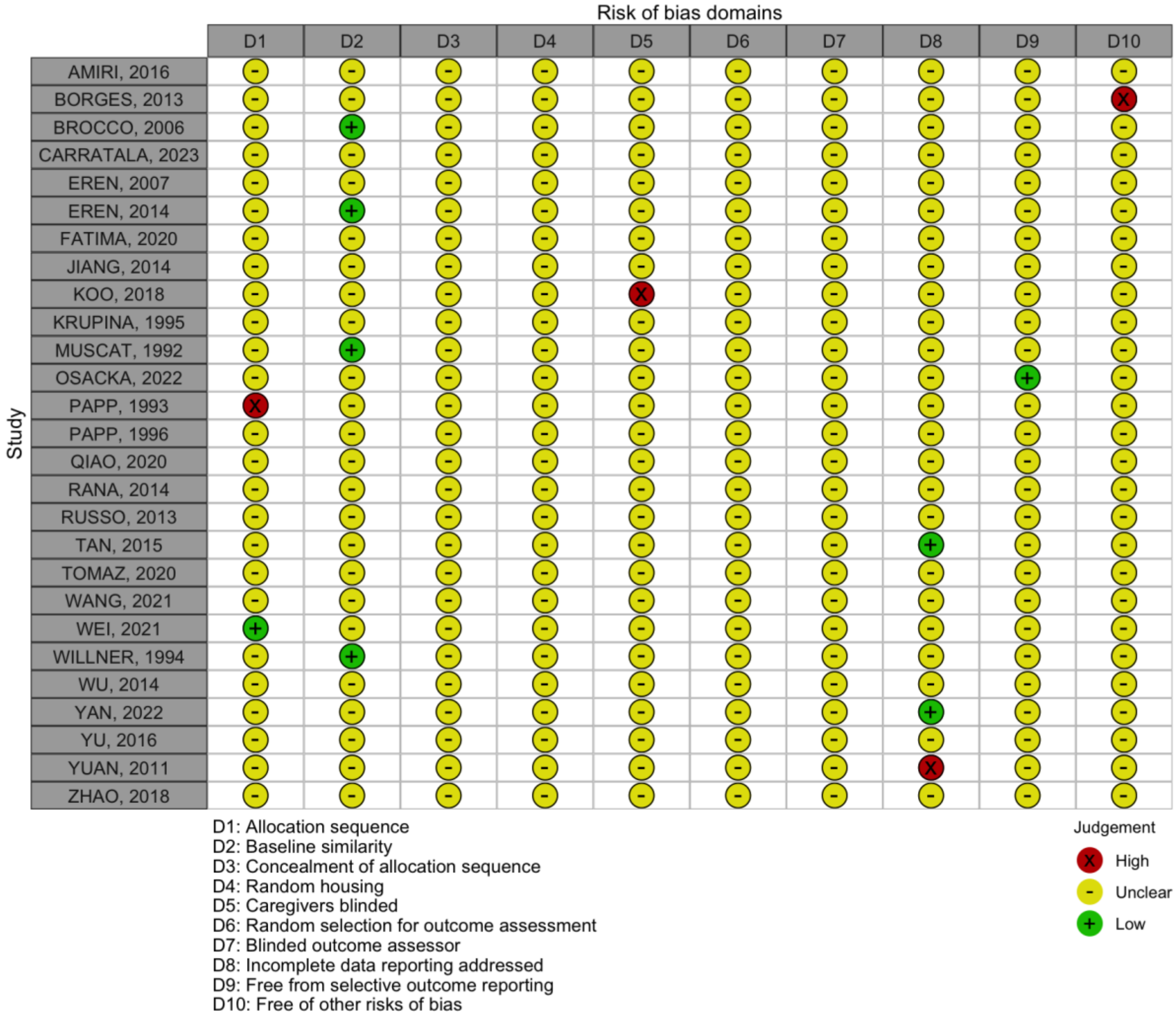

2.1.2 Reporting completeness

Figure 2.1.2 shows the reporting completeness traffic light plot for studies investigating the effect of administering a dopaminergic agent on Sucrose preference in animals. The reporting completeness assessment was performed using the ARRIVE guidelines.

Figure 2.1.2

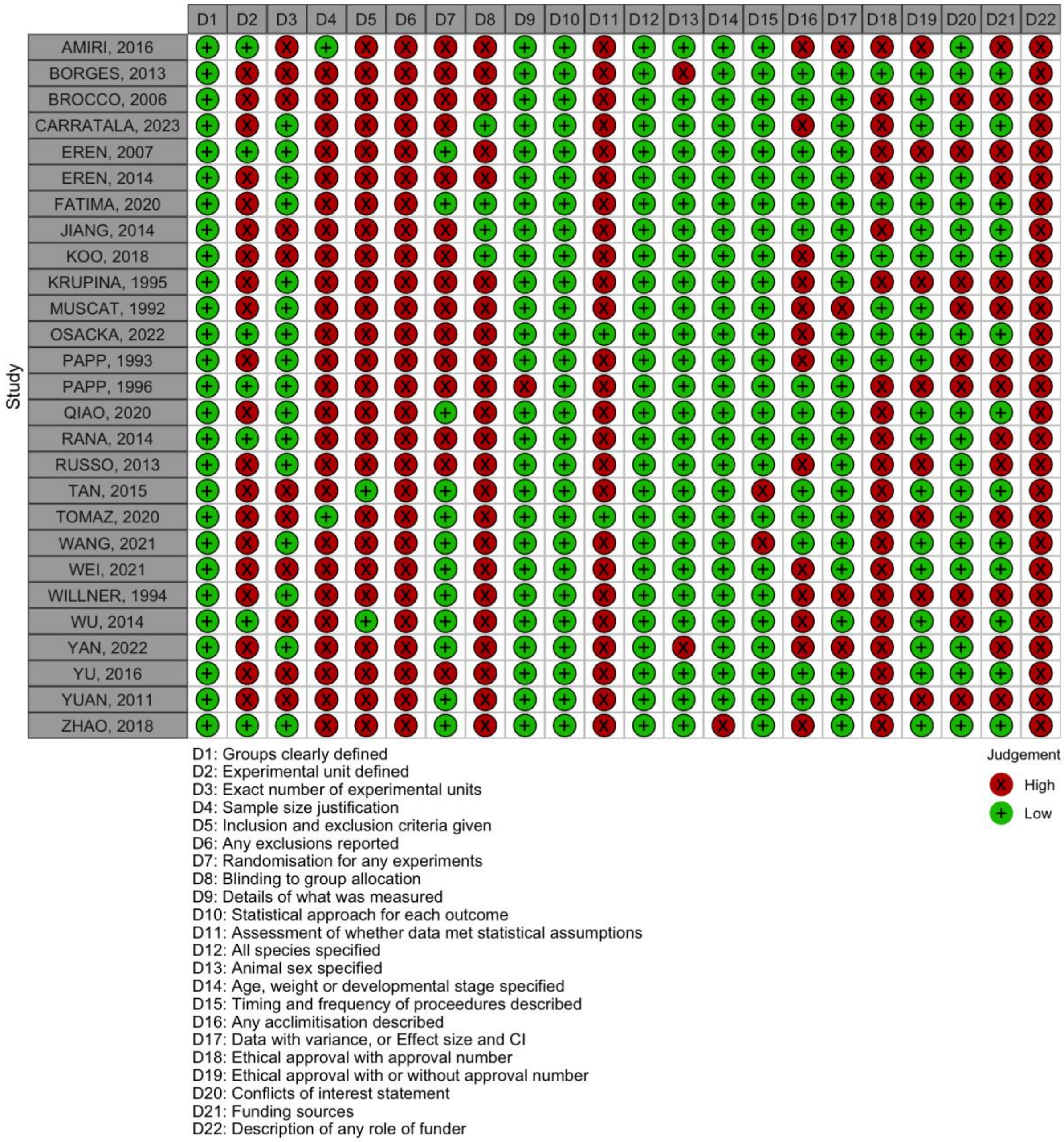

2.1.3 Meta-analysis

The effect of administering a dopaminergic agent on Sucrose preference in animals using SMD as the effect size is shown in Figure 2.1.3. The pooled estimate for SMD across all individual comparisons is displayed as a diamond shape at the bottom of the plot. Grey lines indicate the prediction interval of the pooled estimate.

Figure 2.1.3

Effect of dopaminergic drugs on Sucrose preference test in models of depression (SMD)

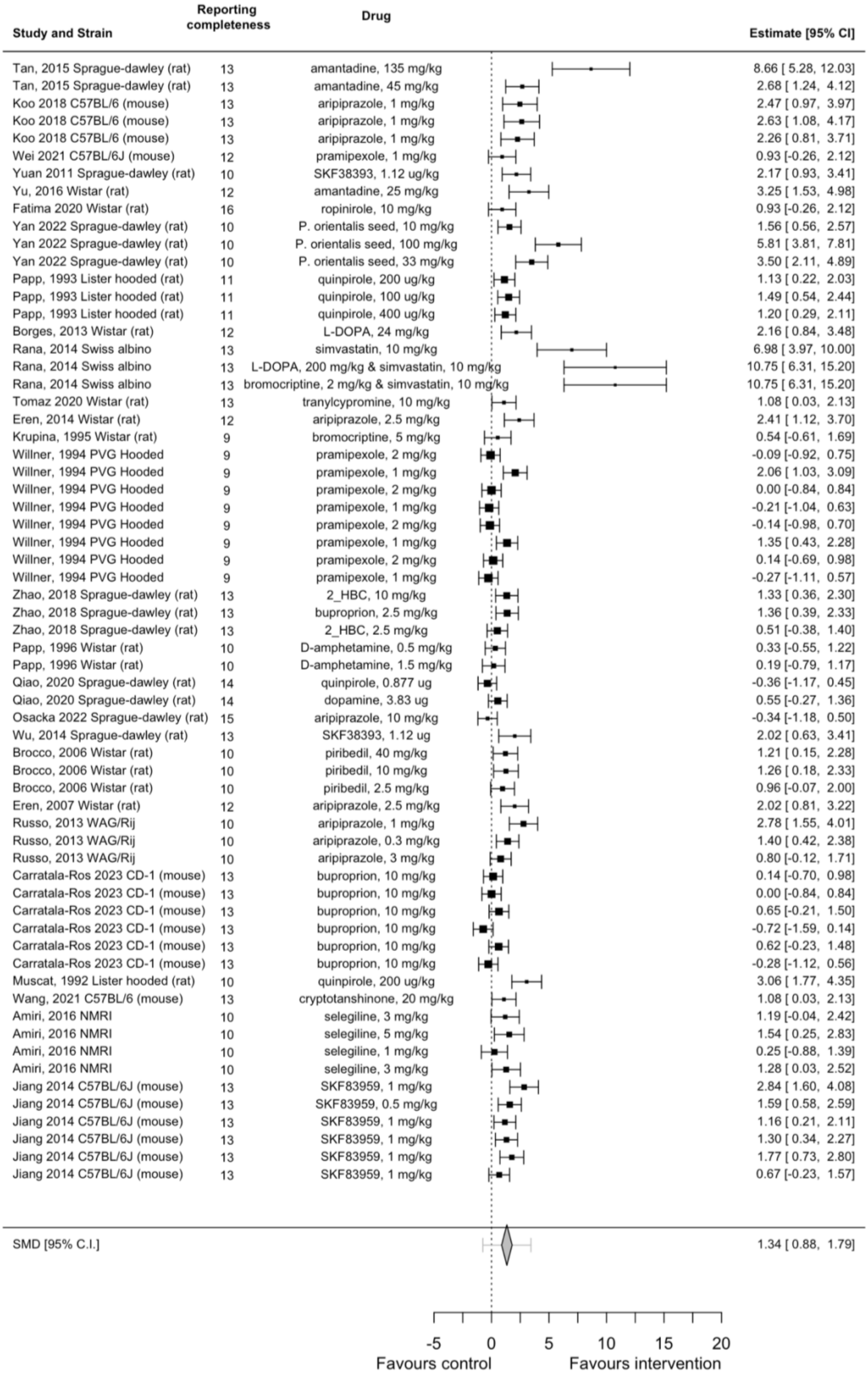

Dopaminergic agents had a pooled effect on Sucrose preference of SMD = 1.335 (95% CI: 0.878 to 1.792; 95% PrI: -0.756 to 3.426).

64 experimental comparisons were reported in 36 experiments reported from 27 publications and involving 10 different animal strains.

The following table structure is used throughout this report and is used to show the different levels contributing to that analysis, the number of unique categories in those levels, and the variance contributed by that level of analysis. Because levels are only included in the analysis where there are five or more unique categories, for some analyses the number of categories is 0, and the variance attributed to those levels in not applicable. Because the model is hierarchical, where for instance there are **Studies** which include different **Strains**, the number of categories for **Study x Strain** will exceed the number of Studies (by which we mean unique publications) referred to in the text.

Level	Number of categories for that level included in this analysis	Attributable variance
Strain	10	0
Study x Strain	27	0.803
Study x Strain x Experiment	36	0.01

2.1.4 Subgroup analyses and meta-regressions

The covariates of interest for subgroup analyses and meta-regressions were:

- **Sex**
- **Category of disease induction**
- **Route of intervention administration**
- **Whether the intervention was prophylactic or therapeutic (i.e. administered before or after disease model induction)**
- **Duration of treatment period**
- **The intervention administered**
- **Dose of intervention**

We also conducted subgroup analyses using **(1) SyRCLE Risk of Bias** and **(2) ARRIVE reporting completeness** assessment scores as covariates to evaluate their influence on effect size estimates. These were not specified in the study protocol, but evaluation of risk of bias is required for the Summary of Evidence table, and no studies were considered at low risk of bias or high reporting completeness to allow such a sensitivity analysis

The significance (p-value) reported is that for a test of whether the moderators are significantly different one from another, rather than whether the effect is significantly different from 0.

Sex

Figure 2.1.4.1 displays the estimates for the pooled SMDs when comparisons are stratified by sex of the animal. Whiskers indicate the 95% confidence interval of each estimate. The overall pooled SMD, not stratified by sex, is displayed as a diamond shape at the bottom of the plot.

Figure 2.1.4.1 - Effect of dopaminergic agent on Sucrose Preference by Sex

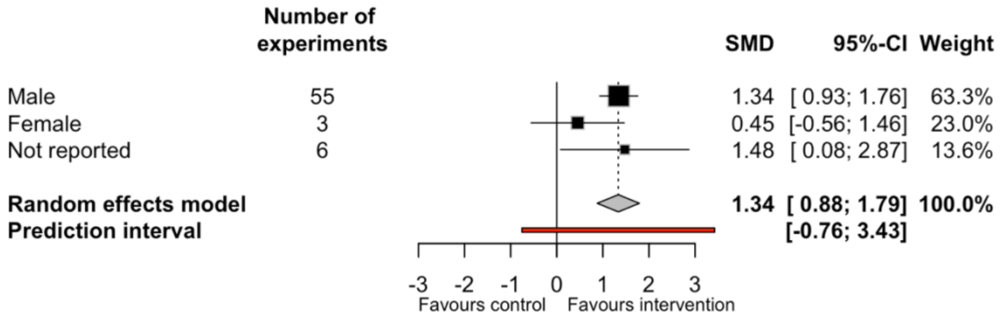

The p-value for the association between the sex of animal groups used and outcome reported was 0.2.

Level	Number of categories for that level included in this analysis	Attributable variance
Strain	10	0
Study x Strain	27	0.804
Study x Strain x Experiment	36	0

Category of disease induction

Figure 2.1.4.2 displays the estimates for the pooled SMDs when comparisons are stratified by the category of disease induction. Whiskers indicate the 95% confidence interval of each estimate. The overall pooled SMD, not stratified by category of disease induction, is displayed as a diamond shape at the bottom of the plot.

Figure 2.1.4.2 - Effect of dopaminergic agent on Sucrose Preference by category of disease induction

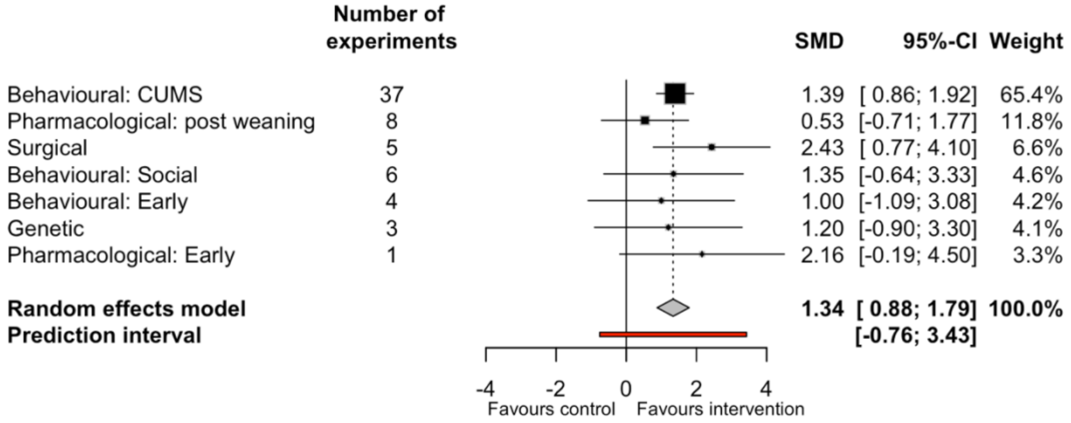

The p-value for the association between whether genetic or pharmacological models were used and outcome reported was 0.7.

Level	Number of categories for that level included in this analysis	Attributable variance
Strain	10	0
Study x Strain	27	0.978
Study x Strain x Experiment	36	0

Route of intervention administration

Figure 2.1.4.3 displays the estimates for the pooled SMDs when comparisons are stratified by the route of intervention administration. Whiskers indicate the 95% confidence interval of each estimate. The overall pooled SMD, not stratified by route of intervention administration, is displayed as a diamond shape at the bottom of the plot.

Figure 2.1.4.3 - Effect of dopaminergic agent on Sucrose Preference by Route of intervention administration

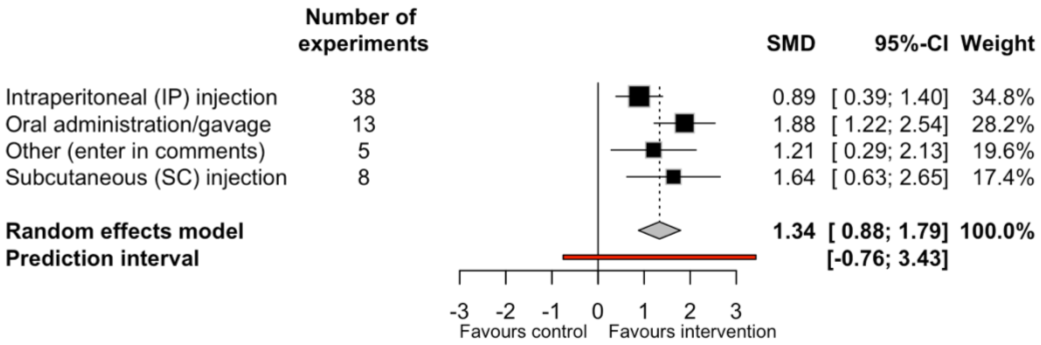

The p-value for the association between the route of intervention administration and outcome reported was 0.214.

Level	Number of categories for that level included in this analysis	Attributable variance
Strain	10	0
Study x Strain	27	0.567
Study x Strain x Experiment	36	0.011

Prophylactic or therapeutic intervention

Figure 2.1.4.4 displays the estimates for the pooled SMDs when comparisons are stratified by whether the intervention was administered prophylactically or therapeutically. Whiskers indicate the 95% confidence interval of each estimate. The overall pooled SMD, not stratified by whether the intervention was administered prophylactically or therapeutically, is displayed as a diamond shape at the bottom of the plot.

Figure 2.1.4.4 - Effect of dopaminergic agent on Sucrose Preference by prophylactic or therapeutic intervention

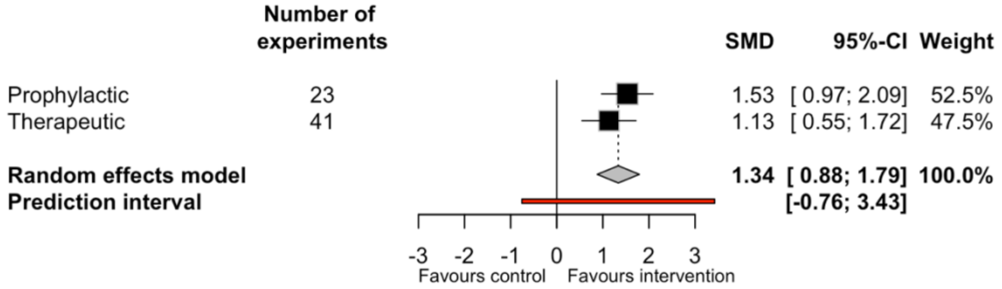

The p-value for the association between whether the intervention was administered prophylactically or therapeutically and outcome reported was 0.344.

Level	Number of categories for that level included in this analysis	Attributable variance
Strain	10	0
Study x Strain	27	0.846
Study x Strain x Experiment	36	0.009

Duration of treatment period

Figure 2.1.4.5 displays the estimates for the pooled SMDs when comparisons are stratified by the duration of treatment. Whiskers indicate the 95% confidence interval of each estimate. The overall pooled SMD, not stratified by duration of treatment, is displayed as a diamond shape at the bottom of the plot.

Figure 2.1.4.5 - Effect of dopaminergic agent on Sucrose Preference by duration of intervention

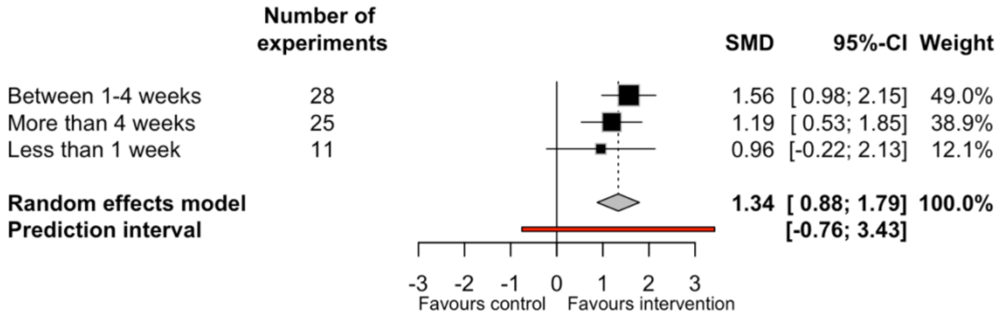

The p-value for the association between the duration of treatment and outcome reported was 0.565.

Level	Number of categories for that level included in this analysis	Attributable variance
Strain	10	0
Study x Strain	27	0.874
Study x Strain x Experiment	36	0.011

The intervention administered

Figure 2.1.4.6 displays the estimates for the pooled SMDs when comparisons are stratified by the intervention administered. Whiskers indicate the 95% confidence interval of each estimate. The overall pooled SMD, not stratified by the intervention administered, is displayed as a diamond shape at the bottom of the plot.

Figure 2.1.4.6 - Effect of dopaminergic agent on Sucrose Preference by intervention administered

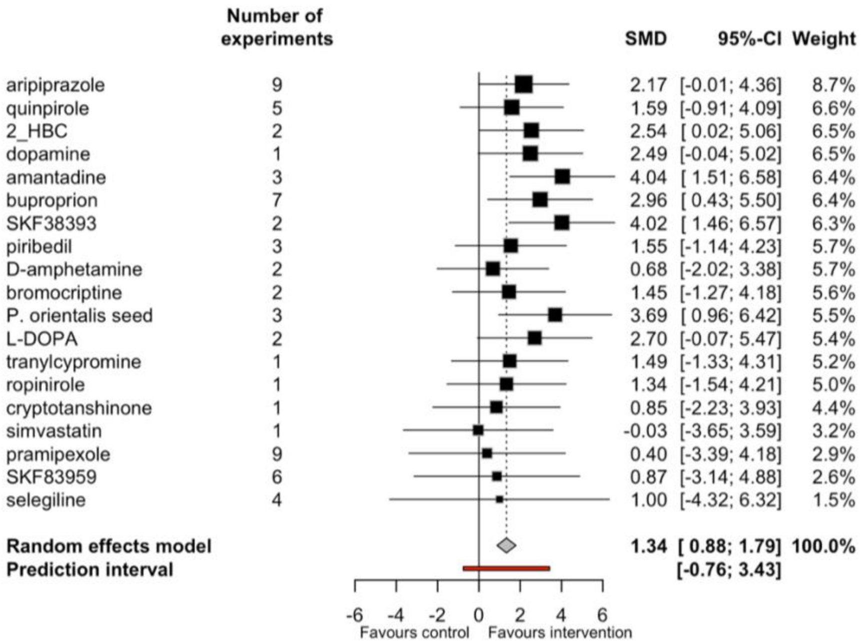

The p-value for the association between the intervention administered and outcome reported was 0.591.

Level	Number of categories for that level included in this analysis	Attributable variance
Strain	10	6.76
Study x Strain	27	0.446

Level	Number of categories for that level included in this analysis	Attributable variance
Study x Strain x Experiment	36	0.005

Dose of intervention

In this iteration of the review, the dopaminergic agents tested against control for their effect on Sucrose preference were: **aripiprazole, pramipexole, buproprion, SKF83959, quinpirole, selegiline, P. orientalis seed, amantadine, piribedil, 2_HBC, D-amphetamine, L-DOPA, SKF38393, bromocriptine, cryptotanshinone, dopamine, ropinirole, simvastatin and tranylcypromine**. Meta-regression using the administered dose as an explanatory variable was conducted for each drug where this had been reported in 10 or more experiments from 3 or more publications. No agent met these criteria.

SyRCLE RoB assessment considered as a categorical variable

Figure 2.1.4.7 displays the estimates for the pooled SMDs when comparisons are stratified by how many of the SyRCLE risk of bias assessment criteria (of which there are 10) that the experiment met. Whiskers indicate the 95% confidence interval of each estimate. The overall pooled SMD, not stratified by SyRCLE Risk of Bias, is displayed as a diamond shape at the bottom of the plot.

Figure 2.1.4.7 - Effect of dopaminergic agent on Sucrose Preference by SyRCLE RoB criteria met

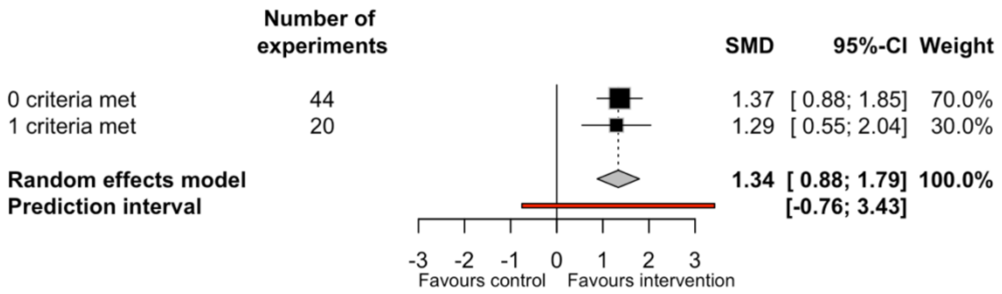

The p-value for the association between SyRCLE Risk of Bias reporting and outcome reported was 0.876.

Level	Number of categories for that level included in this analysis	Attributable variance
Strain	10	0
Study x Strain	27	0.868
Study x Strain x Experiment	36	0.009

SyRCLE RoB assessment considering those studies where any item is at low risk of bias

Figure 2.1.4.8 displays the estimates for the pooled SMDs when comparisons are stratified by whether of not any of the SyRCLE Risk of bias domains were rated as low risk of bias. Whiskers indicate the 95% confidence interval of each estimate. The overall pooled SMD, not stratified by SyRCLE Risk of Bias, is displayed as a diamond shape at the bottom of the plot.

Figure 2.1.4.8 - effect of dopaminergic agent on Sucrose Preference by low SyRCLE RoB reporting

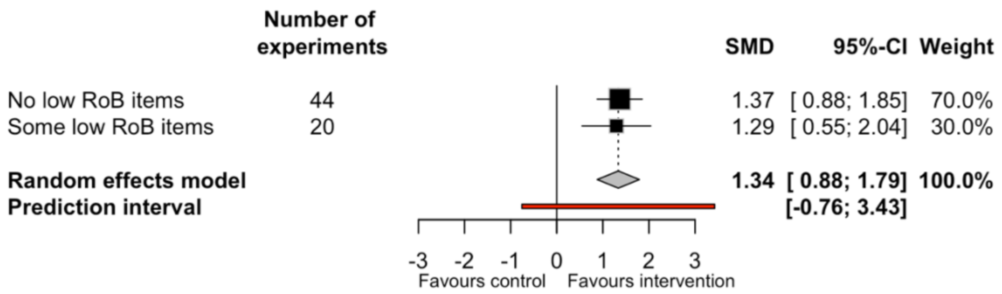

The p-value for the association between low SyRCLE Risk of Bias reporting and outcome reported was 0.876.

Level	Number of categories for that level included in this analysis	Attributable variance
Strain	10	0

Level	Number of categories for that level included in this analysis	Attributable variance
Study x Strain	27	0.868
Study x Strain x Experiment	36	0.009

ARRIVE reporting guidelines performance

We provide a meta-regression where the number of ARRIVE items met is considered as a continuous variable.

Figure 2.1.4.9

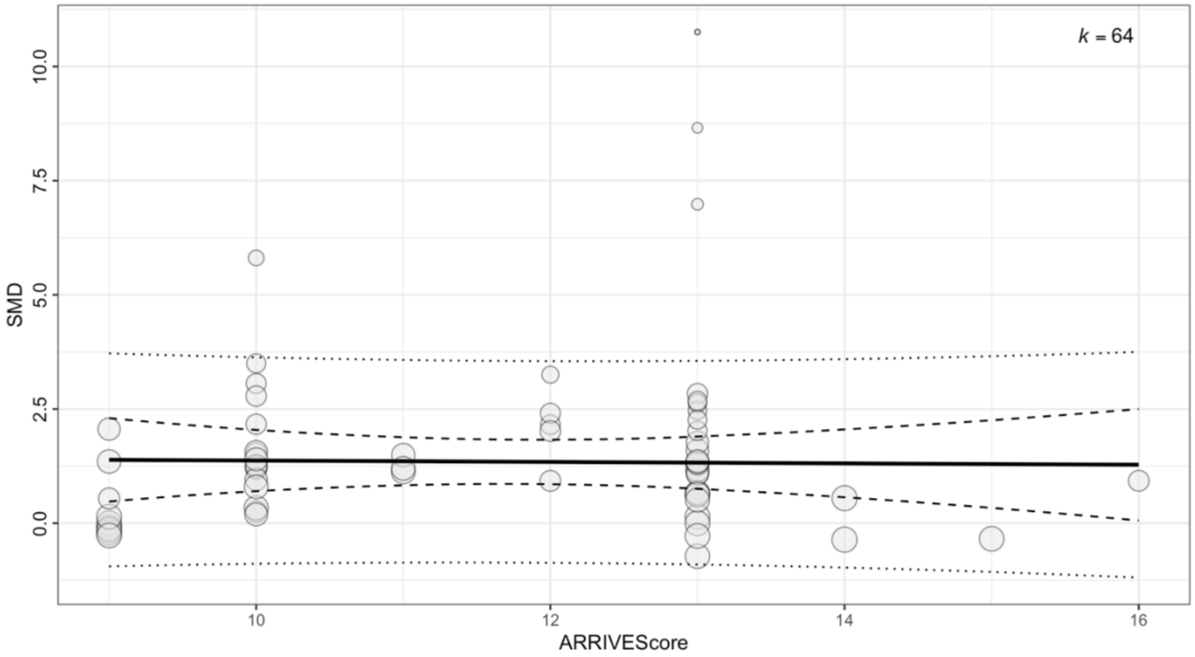

The estimate for ββ was -0.015 (p = 0.894).

Level	Number of categories for that level included in this analysis	Attributable variance
Strain	10	0
Study x Strain	27	0.864
Study x Strain x Experiment	36	0.009

Heterogeneity explained by covariates (Dopaminergic agents and Sucrose preference)

The table below shows which of the covariates, if any, explain some of the heterogeneity observed in the effect sizes of the effect of dopaminergic agents on Sucrose preference. We present marginal R^2, which measures the proportion of variance explained by including moderators in the model (the % change in the between-studies variance when the covariate is included in the model, in other words the % of the heterogeneity explained by the variable). The SMD represent the point estimate and 95% CIs of the effect in each category.

Moderator	Category	SMD	95% CI	Marginal R^2 %
Overall effect (unadjusted model)	-	1.335	0.878 to 1.792	-
Sex	-	-	-	4.6%
-	*Female*	0.454	-0.595 to 1.502	-
-	*Male*	1.345	0.917 to 1.773	-
-	*Not reported*	1.476	0.009 to 2.943	-
Category of disease model induction	-	-	-	16.9%
-	*Behavioural early life stress*	0.998	-2.389 to 3.1	-

Moderator	Category	SMD	95% CI	Marginal R^2 %
-	*Chronic unpredictable mild stress*	1.391	0.831 to 1.952	-
-	*Genetic models*	1.199	-2.212 to 4.611	-
-	*Pharmacological early life stress*	2.158	-0.336 to 4.653	-
-	*Pharmacological post weaning*	0.532	-0.785 to 1.849	-
-	*Social or social defeat stress*	1.345	-0.767 to 3.458	-
-	*Surgical models*	2.434	0.666 to 4.202	-
Administration route	-	-	-	23.3%
-	*Intraperitoneal*	0.894	0.378 to 1.411	-
-	*Oral*	1.879	1.206 to 2.553	-
-	*Other*	1.207	0.234 to 2.18	-
-	*Subcutaneous*	1.638	0.374 to 2.903	-
Prophylactic or therapeutic intervention	-	-	-	4.2%
-	*Prophylactic*	1.53	0.944 to 2.117	-
-	*Therapeutic*	1.132	0.516 to 1.748	-
Duration of treatment period	-	-	-	6%
-	*1 to 4 weeks*	1.564	0.948 to 2.18	-
-	*less than 1 week*	0.956	-0.283 to 2.195	-
-	*4 weeks or more*	1.191	0.5 to 1.882	-

**Moderator**	**Category**	**SMD**	**95% CI**	**Marginal R^2 %**
Intervention administered	-	-	-	15.4%
-	*2-HBC*	2.541	-0.049 to 5.131	-
-	*amantadine*	4.041	1.058 to 7.025	-
-	*aripiprazole*	2.171	-0.399 to 4.741	-
-	*bromocriptine*	1.454	-1.345 to 4.253	-
-	*buproprion*	2.963	0.356 to 5.569	-
-	*cyrptotanshinone*	0.848	-2.774 to 4.47	-
-	D-amphetamine	0.678	-2.496 to 3.852	-
-	*dopamine*	2.494	-0.107 to 5.094	-
-	*L-DOPA*	2.701	-0.147 to 5.549	-
-	*P.orientalis seed*	3.689	0.476 to 6.903	-
-	*piribedil*	1.546	-1.615 to 4.707	-
-	*pramipexole*	0.396	-4.054 to 4.845	-
-	*quinpirole*	1.591	-0.977 to 4.16	-
-	*ropinirole*	1.336	-2.049 to 4.72	-
-	*selegiline*	0.999	-33.48 to 35.477	-
-	*simvastatin*	-0.034	-3.753 to 3.685	-
-	*SKF38393*	4.016	1.011 to 7.021	-

Moderator	Category	SMD	95% CI	Marginal R^2 %
-	*SKF83959*	0.872	-3.847 to 5.591	-
-	*tranylcypromine*	1.491	-1.828 to 4.811	-
Risk of Bias	-	-	-	0.1%
-	*0 criteria met*	1.365	0.853 to 1.878	-
-	*1 criteria met*	1.294	0.511 to 2.077	-
Reporting completeness	-	-	-	0.1%
-	*per unit increase*	-0.015	-0.253 to 0.222	-

2.1.5 Sensitivity Analyses

We examine the robustness of the findings for the primary outcome by performing the following sensitivity analyses

Imputed rho values of 0.2 and 0.8

In the previous analyses for the effect of dopaminergic agents on Sucrose preference, we imputed a ρρ value - the imputed within-study correlation between observed effect sizes - of 0.5. Here, we examine the effect of imputing ρρ values of 0.2 and 0.8.
When the ρρ value is assumed to be 0.2, dopaminergic agents had a pooled effect on Sucrose preference of **SMD = 1.49** (95% CI: 0.95 to 2.03) with a prediction interval of -1.08 to 4.06.
When the ρρ value is assumed to be 0.8, dopaminergic agents had a pooled effect on Sucrose preference of **SMD = 1.07** (95% CI: 0.46 to 1.68) with a prediction interval of -1.16 to 3.3.
For reference the pooled effect size when rho is assumed to be 0.5 is 1.34 (95% CI: 0.88 to 1.79).

NMD

For Sucrose preference, an NMD was calculable for 61 out of 64 comparisons, i.e. 95.31% of comparisons.

The effect of administering a dopaminergic agent on Sucrose preference in animals using NMD as the effect size is shown in Figure 2.1.5. The pooled estimate for NMD across all individual comparisons is displayed as a diamond shape at the bottom of the plot. Dotted lines indicate the prediction interval of the pooled estimate.

Figure 2.1.5

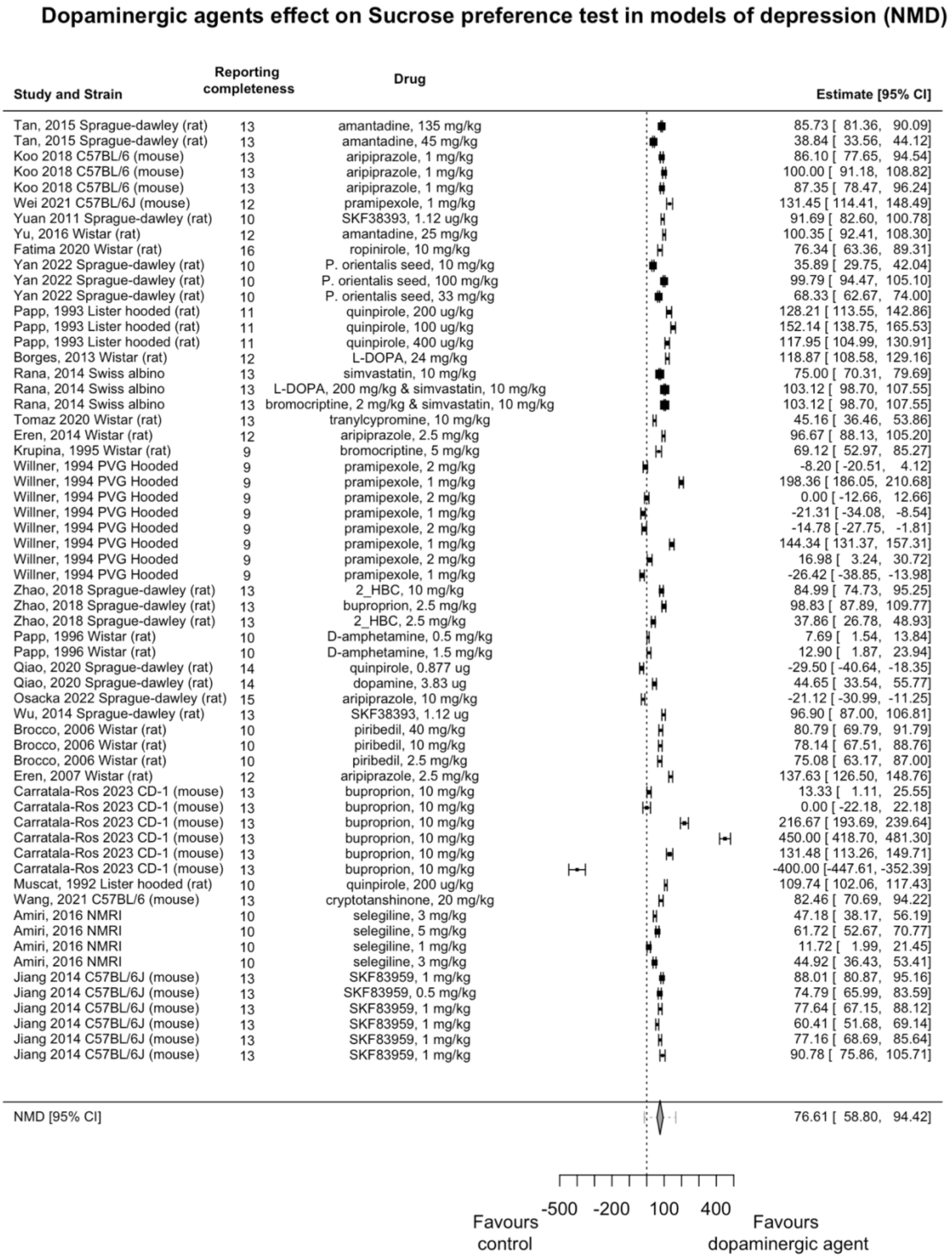

Dopaminergic interventions had a pooled effect on Sucrose preference of NMD = 76.61 (95% CI: 58.8 to 94.42) with a prediction interval of -14 to 167.22).

61 experimental comparisons were reported in 35 experiments reported from 26 publications and involving 9 different animal strains.

Level	Number of categories for that level included in this analysis	Attributable variance
Strain	9	25.54
Study x Strain	26	967.09
Study x Strain x Experiment	35	491.77

2.1.6 Reporting bias/small-study effects

Because of the relationship between SMD effect sizes and variance inherent in their calculation, where study size is small the standard approach to seeking evidence of small-study effects (regression based tests including Egger's regression test for multilevel meta-analysis) can lead to over-estimation of small-study effect (see for instance 10.7554/eLife.24260). To address this we used Egger's regression test for multilevel meta-analysis, with regression of SMD effect size against $1/\sqrt{N}$, where N is the total number of animals involved in an experiment.

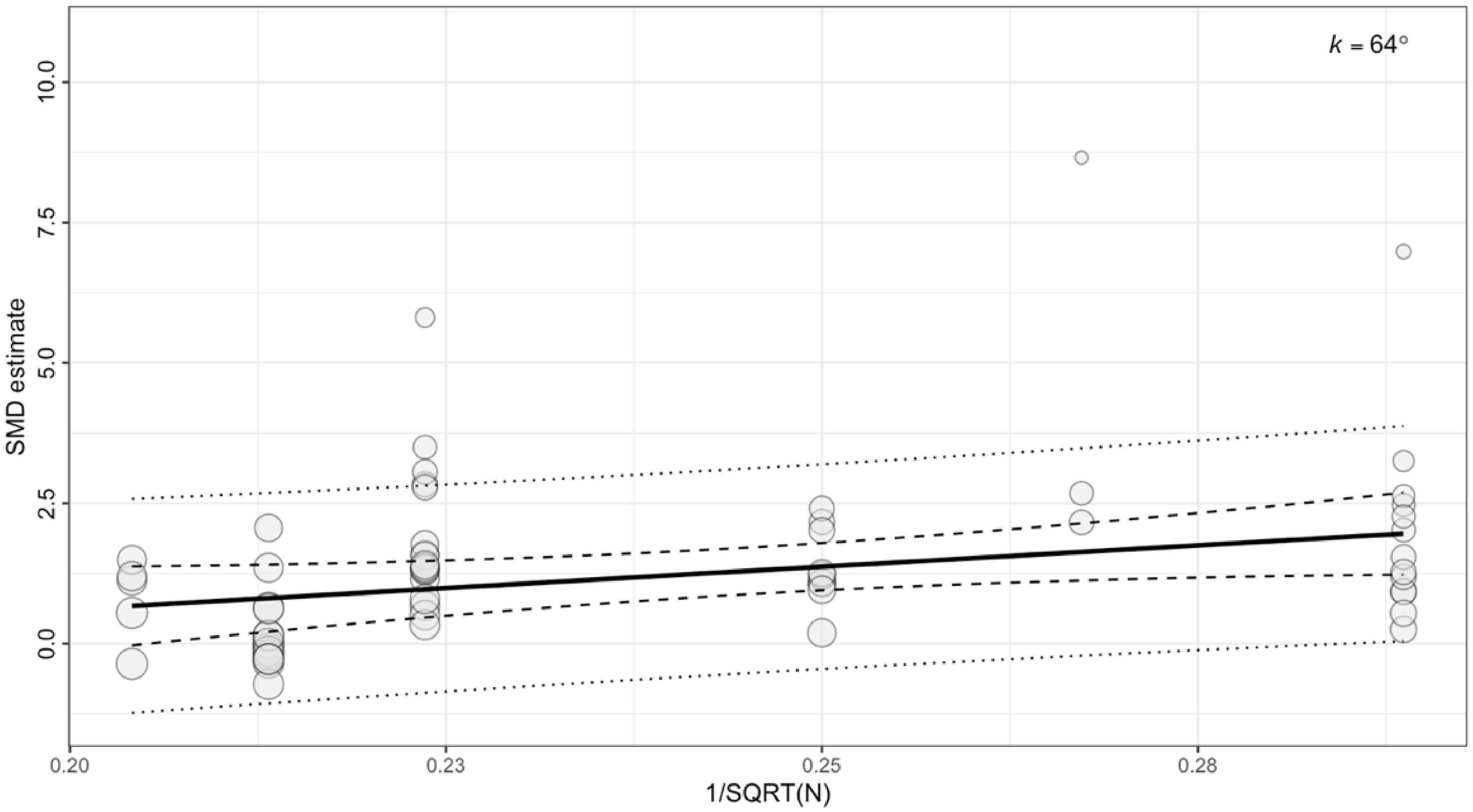

Egger regression based on 64 studies of dopaminergic agents v Control where Sucrose preference was measured showed a coefficient for a small study effect of 15.22 (95% CI: 3.25 to 27.19; p = 0.014) in the context of a baseline estimate of effect of -2.44 (95% CI: -5.79 to 0.92; p = 0.133).

2.2 Outcome 2: Dopamine concentration

2.2.1 Risk of bias

Figure 2.2.1a shows the risk of bias summary for studies investigating the effect of administering a dopaminergic agent on dopamine concentrations in animals. The risk of bias assessment was performed using the SyRCLE's RoB tool. Figure 2.2.1b shows the corresponding traffic light plot.

Figure 2.2.1

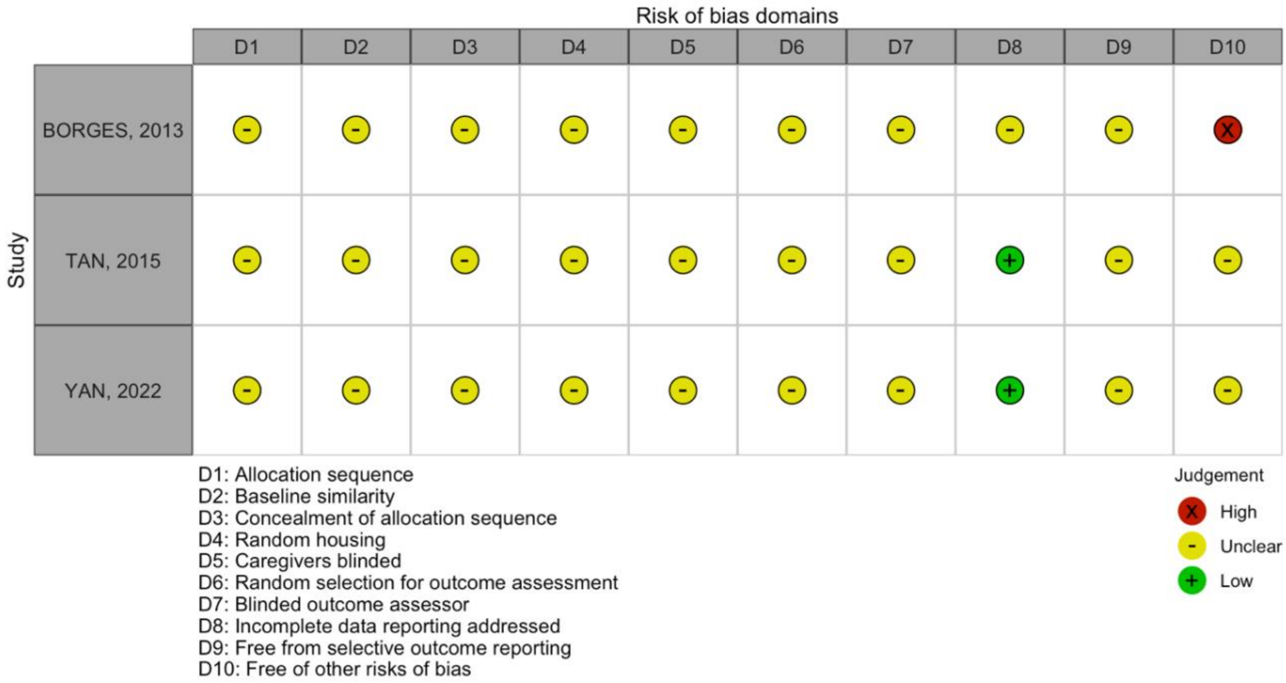

2.2.2 Reporting completeness

Figure 2.2.2a shows the reporting completeness summary for studies investigating the effect of administering a dopaminergic agent on dopamine concentrations in animals. The reporting completeness assessment was performed using the ARRIVE guidelines. Figure 2.2.2b shows the corresponding traffic light plot.

Figure 2.2.2

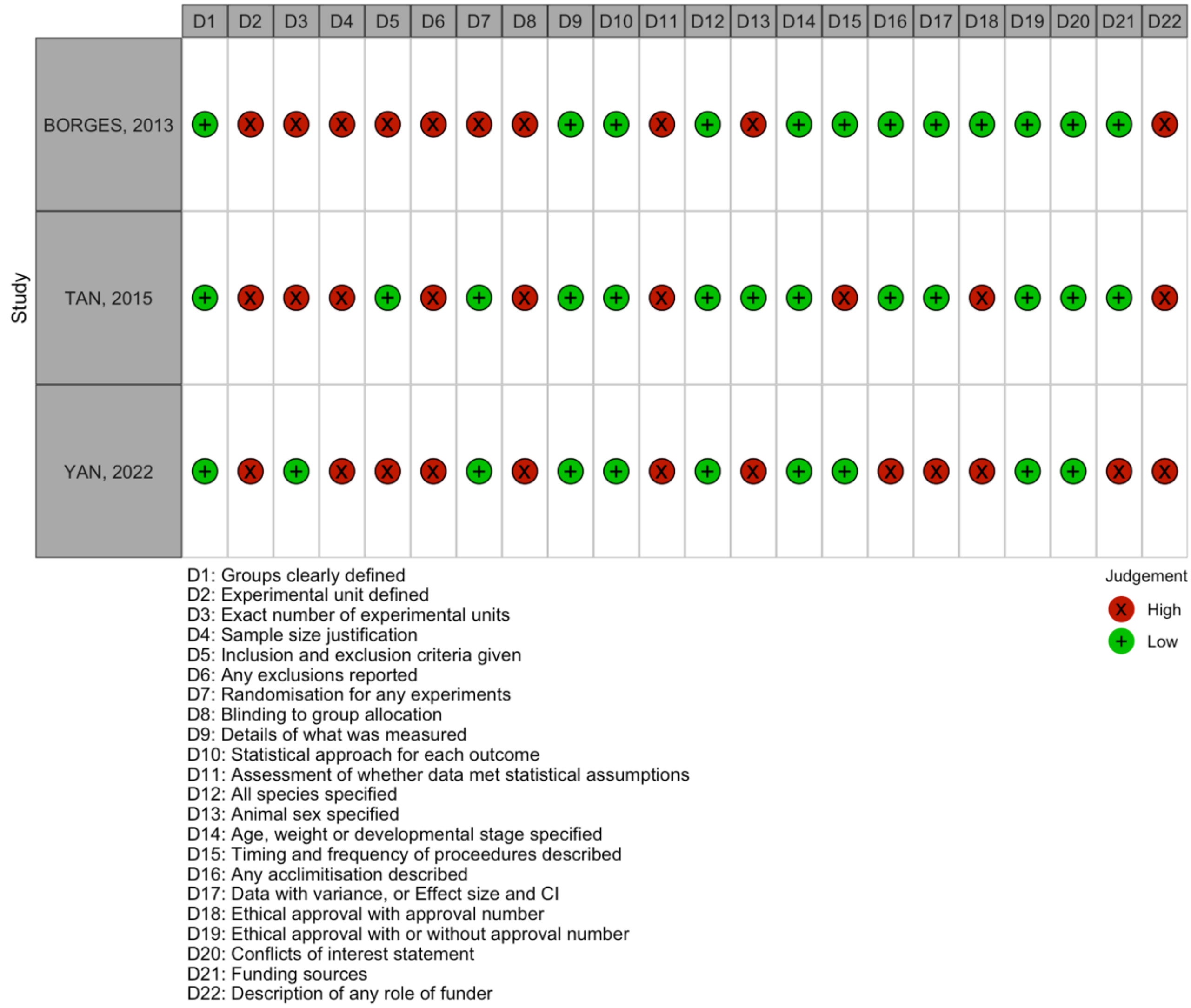

2.2.3 Meta-analysis

Multilevel analysis is only performed if there are 5 levels or more for at least one of Strain, Study and Experiment, and that is not the case here. 13 experimental comparisons were reported in 3 experiments reported from 3 publications and involving 2 different animal strains. We provide a conventional random effects model to illustrate the data. No subgroup analysis is performed.

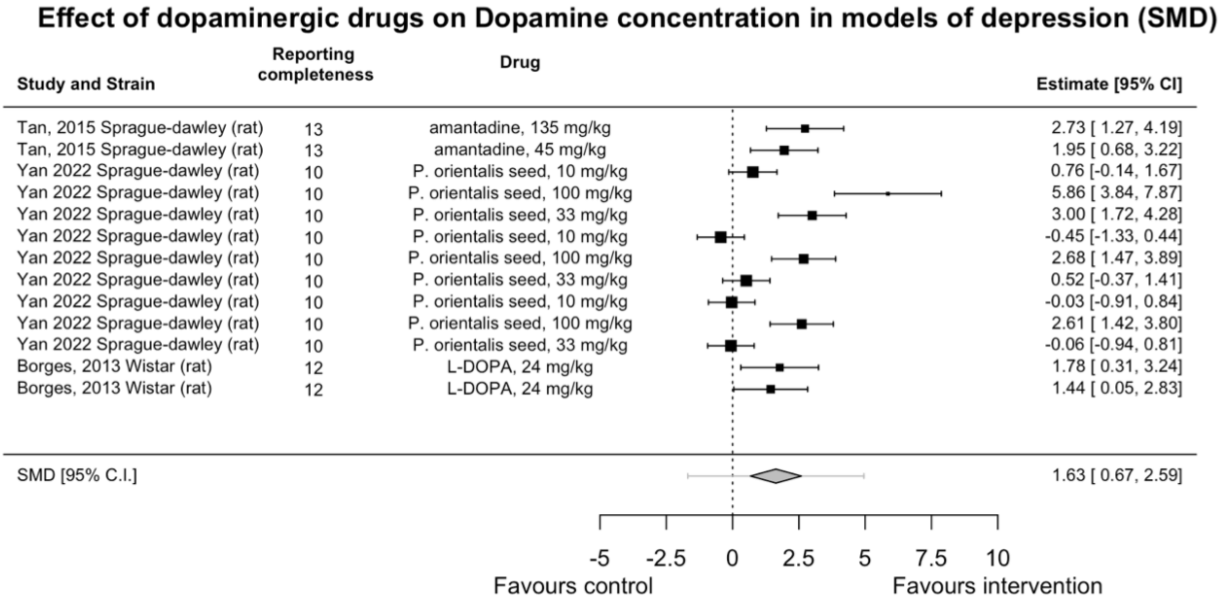

Dopaminergic agents had a pooled effect on dopamine concentration of SMD = 1.63 (95% CI: 0.67 to 2.59; 95% PrI: -1.69 to 4.95). 13 experimental comparisons were reported in 3 experiments reported from 3 publications and involving 2 different animal strains.

2.3 Outcome 3: DOPAC concentration

This was only reported in 2 studies, so no further analysis will be performed.

2.4 Outcome 4: Dopamine / DOPAC ratio

This was only reported in 1 study, so no further analysis will be performed.

3 Effects of model induction

To provide context for the effects of dopaminergic agents described above, we also present findings from expriments where no therapeutic intervention was given, which have simply reported the effects on apical (sucrose preference test) and other (dopamine, DOPAC, Da/DOPAC ratio, dopamine receptor biology) of model induction. Modelling interventions comprise behavioural (31 experiments), pharmacological (9) and surgical (4) approaches.

26 studies (58 comparisons) investigated the effects of model induction. The number of studies and individual effect sizes for each outcome were:

- **Sucrose preference*:** 26 studies and 42 comparisons in 9 strains
- Dopamine concentration: 4 studies and 7 comparisons in 2 strains
- DOPAC concentration: 2 studies and 3 comparisons in 2 strains
- DA/DOPAC ratio: 1 studies and 3 comparisons in 1 strain
- Dopamine receptor biology: 2 studies and 3 comparisons in 2 strains

* This outcome was identified in the study protocol as the primary outcome of interest.

3.1 Outcome 1: Sucrose preference

3.1.1 Risk of bias

Figure 3.1.1 shows the risk of bias summary for studies investigating the effect of modelling depression on sucrose preference in animals. The risk of bias assessment was performed using the SyRCLE RoB tool.

Figure 3.1.1

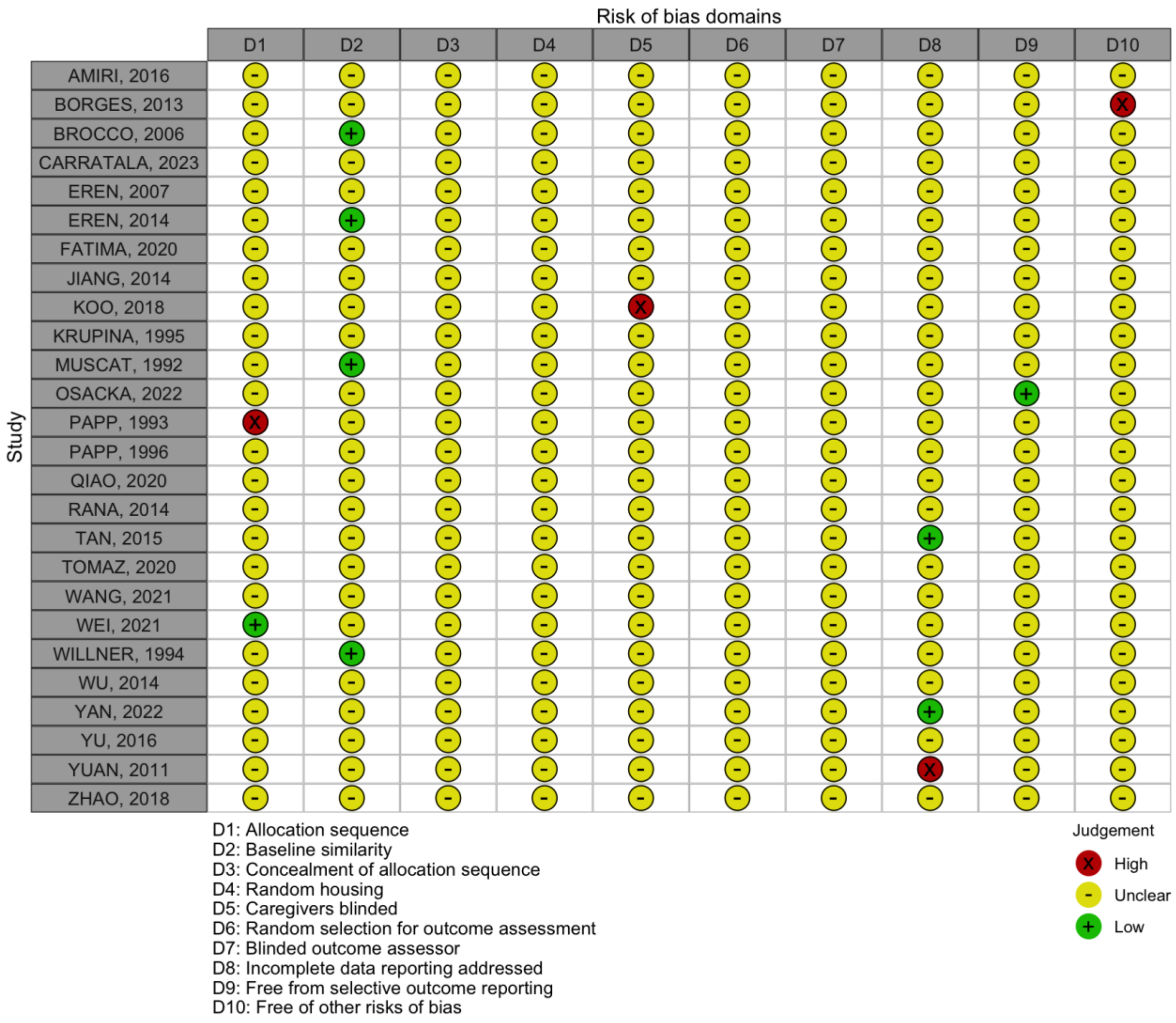

3.1.2 Reporting completeness

Figure 3.1.2 shows the reporting completeness summary for studies investigating the effect of modelling depression on sucrose preference in animals. The reporting completeness assessment was performed using the ARRIVE guidelines.

Figure 3.1.2

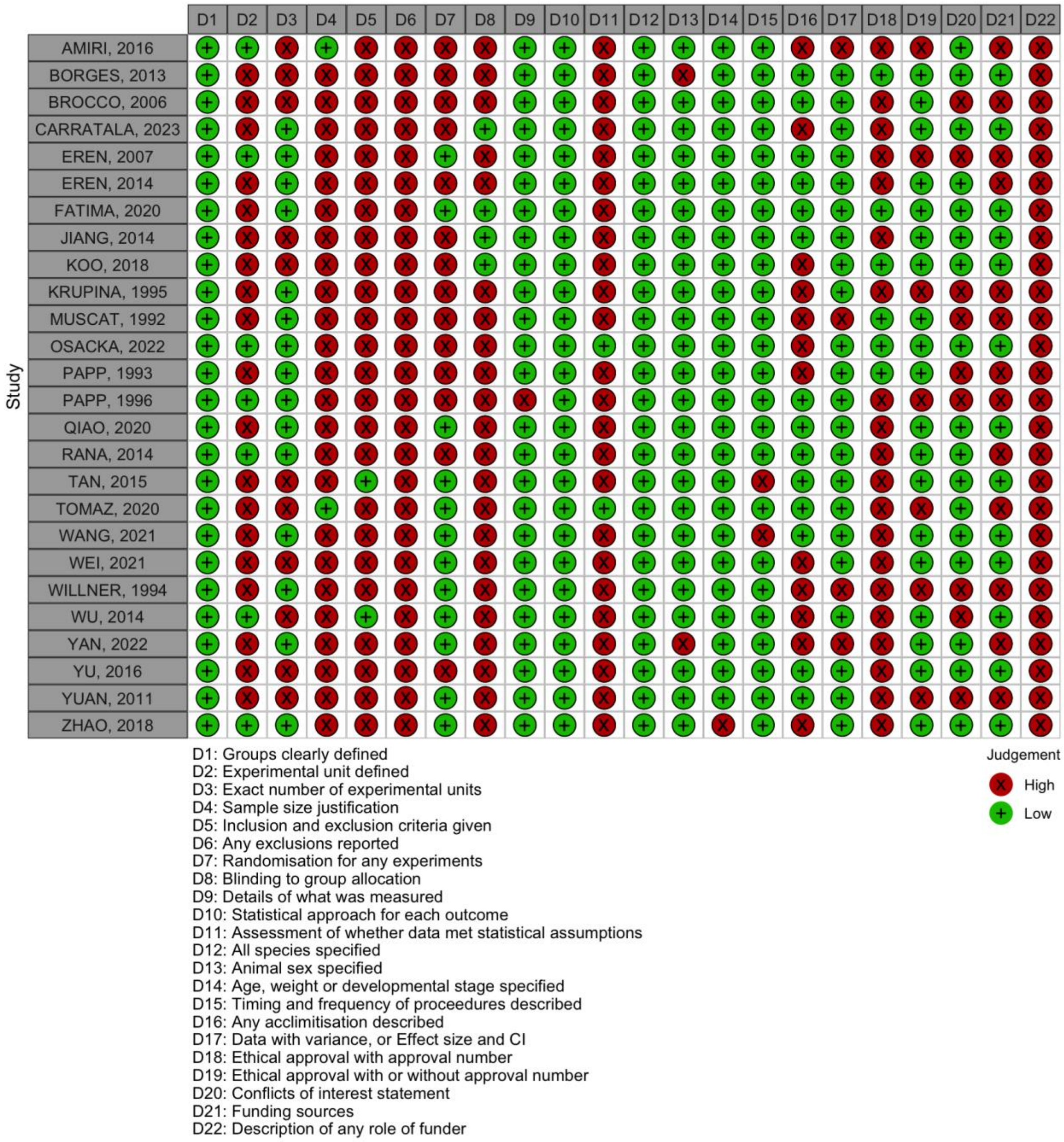

3.1.3 Meta-analysis

The effect of modelling depression on sucrose preference in animals using SMD as the effect size is shown in Figure 3.1.3. The pooled estimate for SMD across all individual comparisons is displayed as a diamond shape at the bottom of the plot. Grey lines indicate the prediction interval (PrI) of the pooled estimate.

Figure 3.1.3

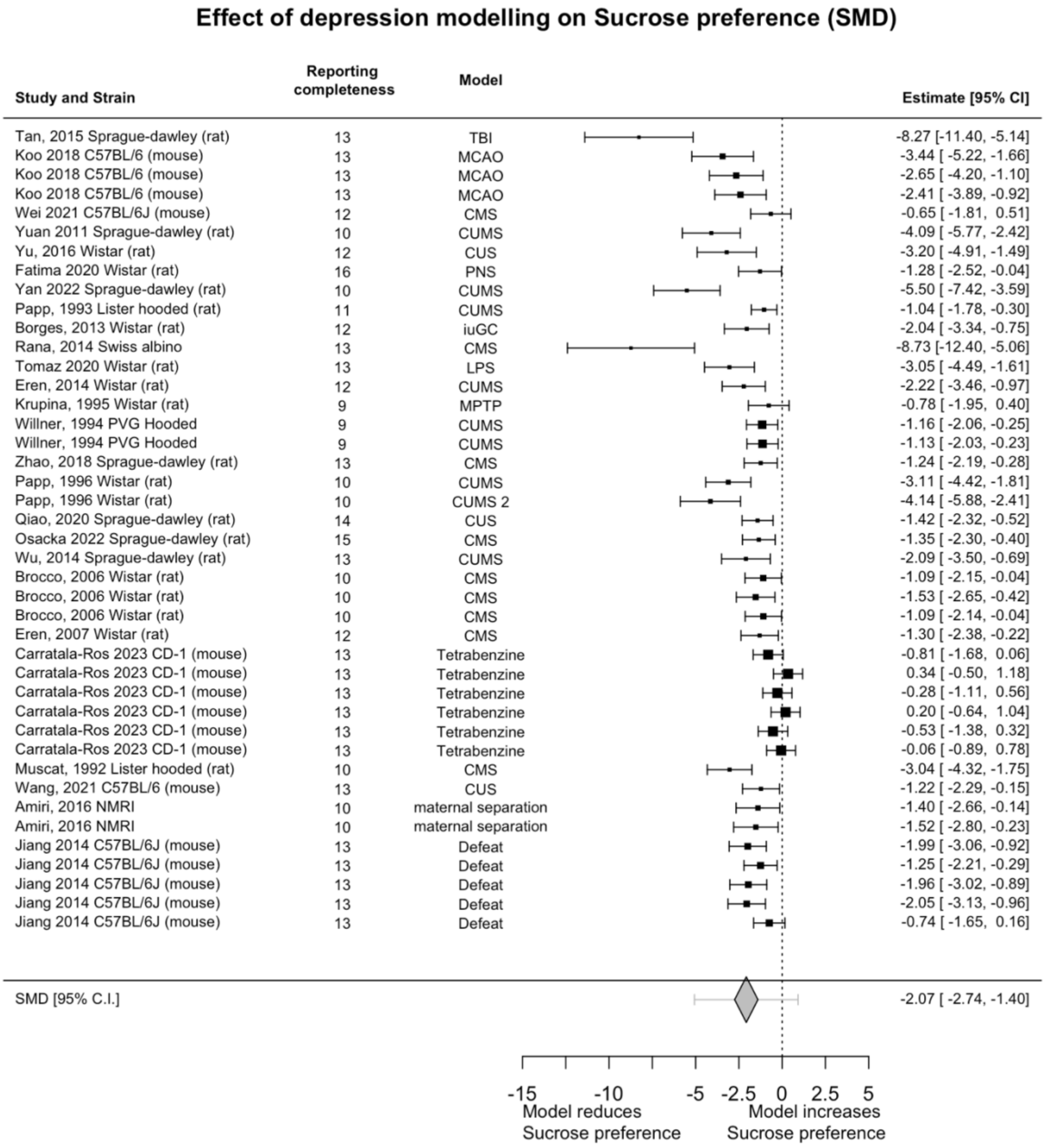

Depression modelling had a pooled effect on sucrose preference of SMD = -2.07 (95% CI: -2.74 to -1.4; 95% PrI: -5.06 to 0.92).

42 experimental comparisons were reported in 35 experiments reported from 26 publications and involving 9 different animal strains.

Level	Number of categories for that level included in this analysis	Attributable variance
Strain	9	0.05
Study x Strain	26	1.52
Study x Strain x Experiment	35	0.03

3.1.4 Subgroup analyses and meta-regressions

The covariates of interest for subgroup analyses and meta-regressions were:

- **Sex**
- **Method of disease induction**

We also conducted subgroup analyses using **(1) SyRCLE Risk of Bias** and **(2) ARRIVE reporting completeness** assessment scores as covariates to evaluate their influence on effect size estimates. These were not specified in the study protocol, but evaluation of risk of bias is required for the Summary of Evidence table, and no studies were considered entirely at low risk of bias or of high reporting completeness to allow such a sensitivity analysis. The significance (p-value) reported is that for a test of whether the moderators are significantly different one from another, rather than whether the effect is significantly different from 0.

Sex

Figure 3.1.4.1 displays the estimates for the pooled SMDs when comparisons are stratified by sex of the animal. Whiskers indicate the 95% confidence interval of each estimate. The overall pooled SMD, not stratified by sex, is displayed as a diamond shape at the bottom of the plot.

Figure 3.1.4.1 - Effect of modelling depression on sucrose preference by sex

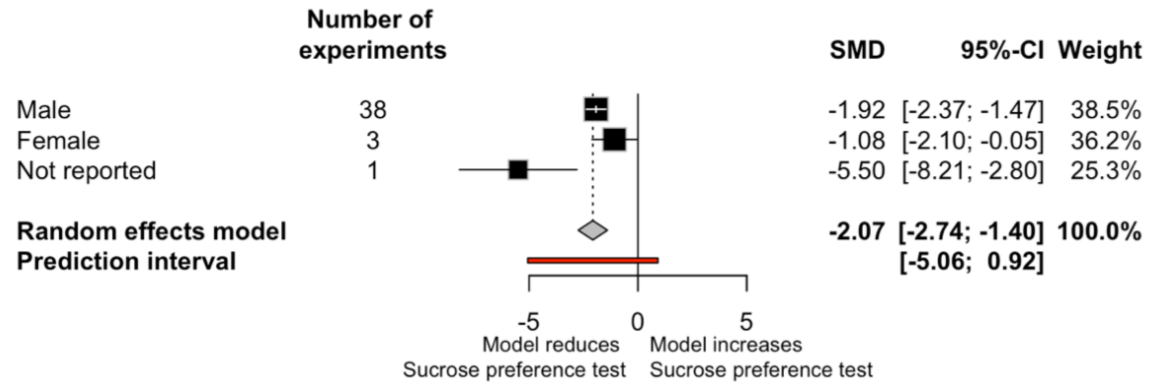

The p-value for the association between the sex of animal groups used and outcome reported was 0.017.

Level	Number of categories for that level included in this analysis	Attributable variance
Strain	9	0
Study x Strain	26	0.944
Study x Strain x Experiment	35	0

Method of disease induction

Figure 3.1.4.2 displays the estimates for the pooled SMDs when comparisons are stratified by the category of disease induction. Whiskers indicate the 95% confidence interval of each estimate. The overall pooled SMD, not stratified by category of disease induction, is displayed as a diamond shape at the bottom of the plot.

Figure 3.1.4.2 - Effect of modelling depression on sucrose preference by category of disease induction

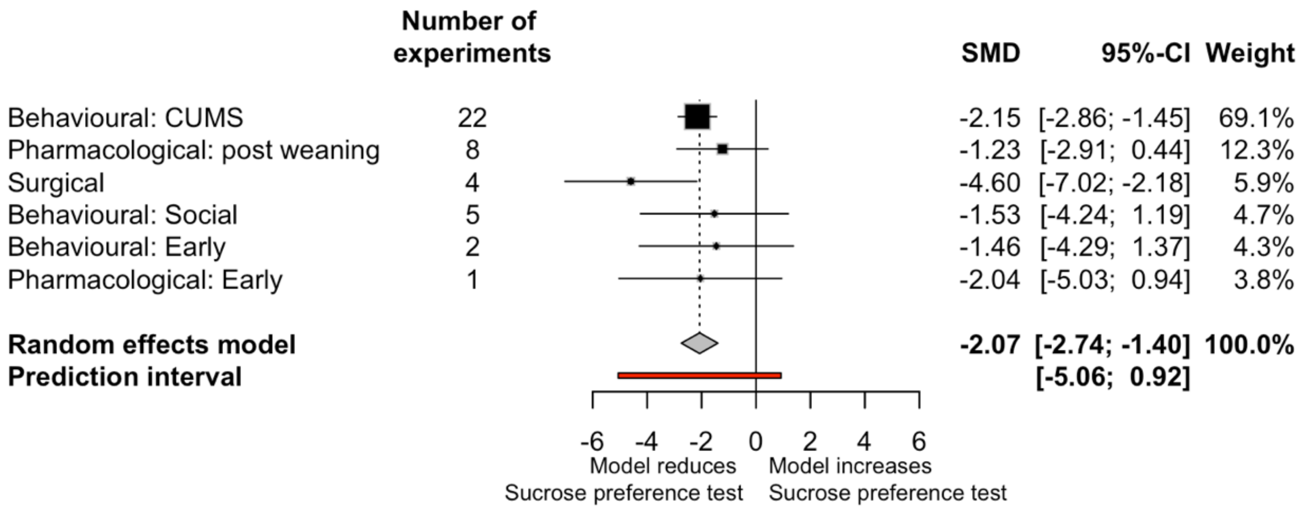

The p-value for the association between the depression model used and the outcome reported was 0.5.

Level	Number of categories for that level included in this analysis	Attributable variance
Strain	9	0
Study x Strain	26	1.862
Study x Strain x Experiment	35	0.029

SyRCLE RoB assessment considered as a categorical variable

Figure 3.1.4.3 displays the estimates for the pooled SMDs when comparisons are stratified by how many of the SyRCLE risk of bias assessment criteria (of which there are 10) that the experiment met. Whiskers indicate the 95% confidence interval of each estimate. The overall pooled SMD, not stratified by SyRCLE Risk of Bias, is displayed as a diamond shape at the bottom of the plot.

Figure 3.1.4.3 - Effect of modelling depression on sucrose preference by SyRCLE RoB criteria met

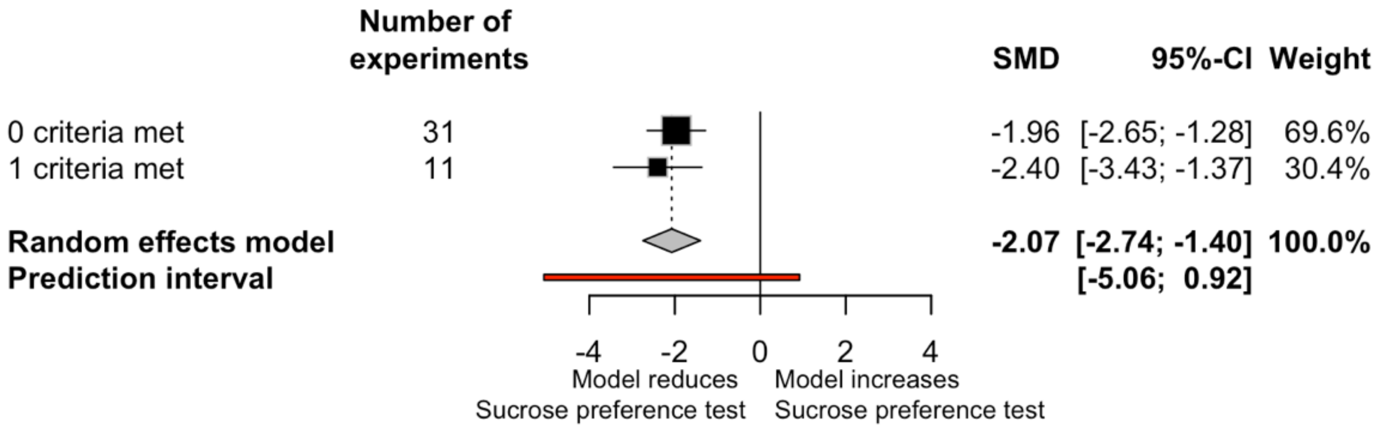

The p-value for the association between SyRCLE Risk of Bias reporting and outcome reported was 0.492.

Level	Number of categories for that level included in this analysis	Attributable variance
Strain	9	0.02
Study x Strain	26	1.667
Study x Strain x Experiment	35	0.03

SyRCLE RoB assessment considering those studies where any item is at low risk of bias

Figure 3.1.4.4 displays the estimates for the pooled SMDs when comparisons are stratified by whether of not any of the SyRCLE Risk of bias domains were rated as low risk of bias. Whiskers indicate the 95% confidence interval of each estimate. The overall pooled SMD, not stratified by SyRCLE Risk of Bias, is displayed as a diamond shape at the bottom of the plot.

Figure 3.1.4.4 - Effect of modelling depression on sucrose preference by low SyRCLE RoB

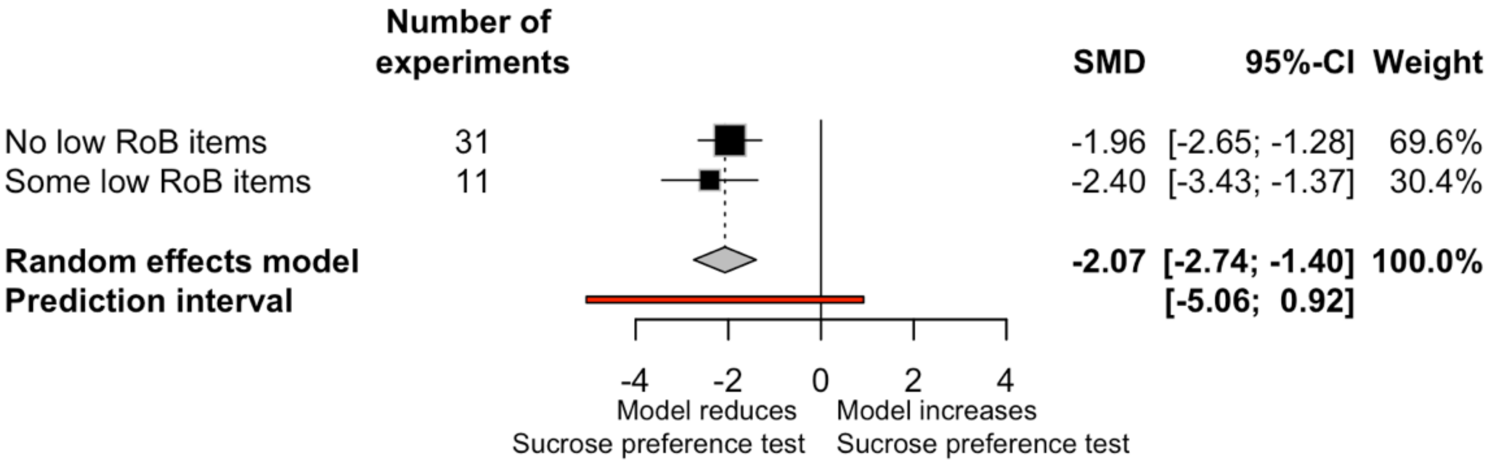

The p-value for the association between low SyRCLE Risk of Bias reporting and outcome reported was 0.492.

Level	Number of categories for that level included in this analysis	Attributable variance
Strain	9	0.02
Study x Strain	26	1.667
Study x Strain x Experiment	35	0.03

ARRIVE reporting completeness guidelines

We provide a metaregression where the number of ARRIVE items met is considered as a continuous variable.

Figure 3.1.4.5

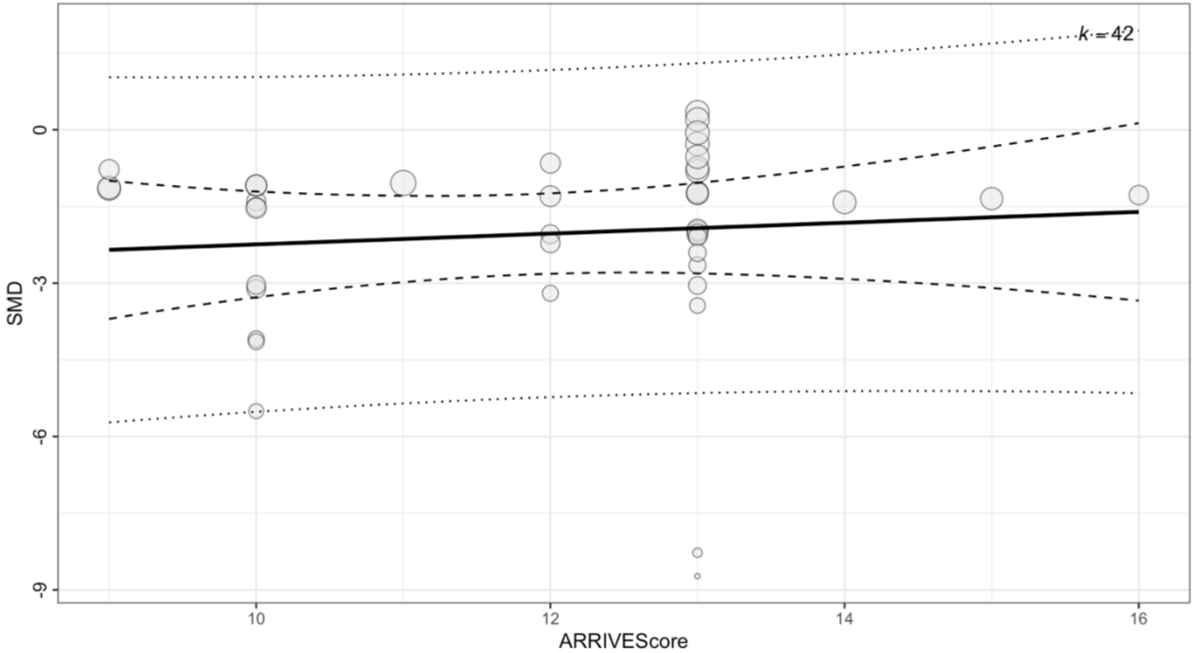

The p-value for the association between ARRIVE reporting completeness and outcome reported was 0.507.

Level	Number of categories for that level included in this analysis	Attributable variance
Strain	9	0.178
Study x Strain	26	1.513
Study x Strain x Experiment	35	0.031

Heterogeneity explained by covariates (Effect of modelling on Sucrose preference)

The table below shows which of the covariates, if any, explain some of the heterogeneity observed in the effect sizes of the effect of TAAR1 agonists on Sucrose preference. We present marginal R^2, which measures the proportion of variance explained by including moderators in the model (the % change in the between-studies variance when the covariate is included in the model, in other words the % of the heterogeneity explained by the variable). The SMD represent the point estimate and 95% CIs of the effect in each category.

Moderator	Category	SMD	95% CI	Marginal R^2 %
Overall effect (unadjusted model)	-	-2.072	-2.745 to -1.399	-
Sex	-	-	-	27.9%
-	*Female*	-1.077	-2.145 to -0.01	-
-	*Male*	-1.922	-2.392 to -1.453	
-	*Not reported*	-5.504	-8.357 to -2.652	
Category of disease model induction	-	-	-	30.2%
-	*Behavioural early life stress*	-1.458	-6.055 to 5.433	-
-	*Chronic unpredictable mild stress*	-2.153	-2.903 to -1.403	-
-	*Pharmacological early life stress*	-2.044	-5.225 to 1.137	-

Moderator	Category	SMD	95% CI	Marginal R^2 %
-	*Pharmacological post weaning*	-1.232	-3.013 to 0.549	-
-	*Social or social defeat stress*	-1.458	-6.055 to 3.14	-
-	*Surgical models*	-4.598	-7.174 to -2.022	-
Risk of Bias	-	-	-	2.1%
-	*0 criteria met*	-1.965	-2.682 to -1.247	-
-	*1 criteria met*	-2.401	-3.488 to -1.315	-
Reporting completeness	-	-	-	1.8%
-	*per unit increase*	0.106	-0.219 to 0.43	-

3.1.5 Sensitivity Analyses

We examine the robustness of the findings for the primary outcome by performing the following sensitivity analyses

Imputed rho values of 0.2 and 0.8

In the previous analyses for the effect of depression modelling on sucrose preference, we imputed a ρρ value - the imputed within-study correlation between observed effect sizes - of 0.5. Here, we examine the effect of imputing ρρ values of 0.2 and 0.8.

When the ρρ value is assumed to be 0.2, depression modelling had a pooled effect on sucrose preference of **SMD = -2.08** (95% CI: -2.75 to -1.41) with a prediction interval of -5.08 to 0.92.

When the ρρ value is assumed to be 0.8, depression modelling had a pooled effect on sucrose preference of **SMD = -2.05** (95% CI: -2.73 to -1.37) with a prediction interval of -5.03 to 0.92.

For reference the pooled effect size when rho is assumed to be 0.5 is -2.07 (95% CI: -2.74 to -1.4), so it is robust to variations in the within-study correlations.

NMD

NMD analysis is not applicable to the analysis of the effects of disease modelling.

3.1.6 Reporting bias/small-study effects

Because of the relationship between SMD effect sizes and variance inherent in their calculation, where study size is small the standard approach to seeking evidence of small-study effects (regression based tests including Egger's regression test for multilevel meta-analysis) can lead to over-estimation of small-study effect (see for instance 10.7554/eLife.24260). To address this we used Egger's regression test for multilevel meta-analysis, with regression of SMD effect size against 1/√N, where N is the total number of animals involved in an experiment.

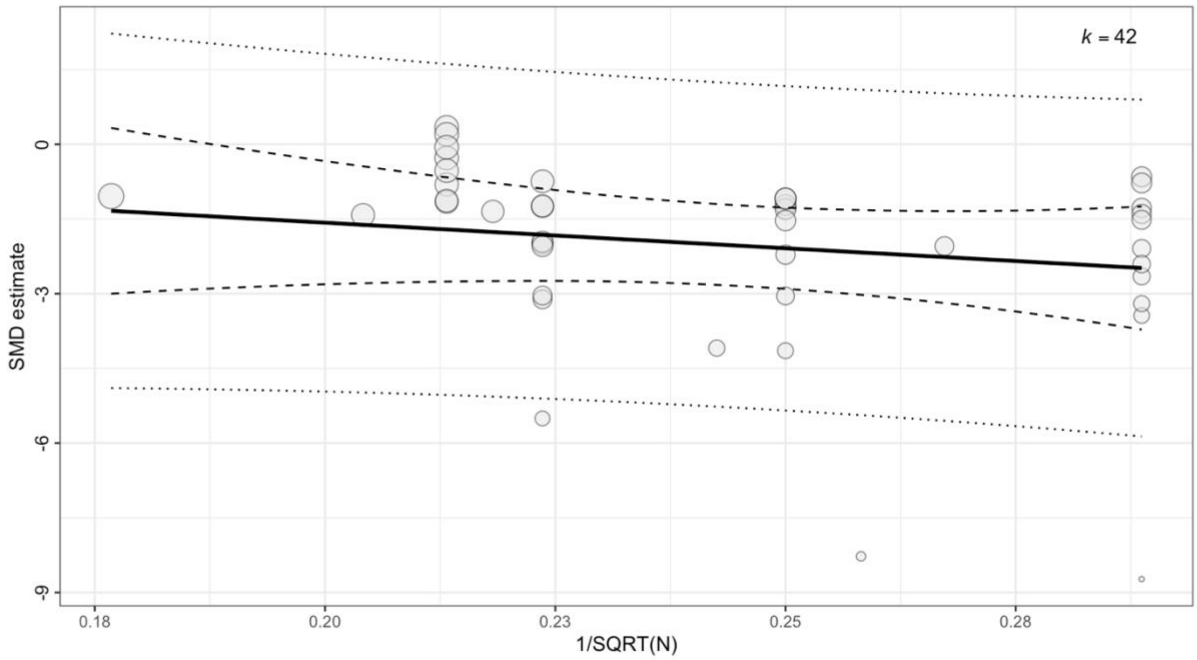

Egger regression based on 42 studies of modelling of depression where sucrose preference was measured showed a coefficient for a small study effect of -10.26 (95% CI: -28.35 to 7.82; p = 0.257).

3.2 Outcome 2: Dopamine concentrations

3.2.1 Risk of bias

Figure 3.2.1 shows the risk of bias traffic light plot for studies investigating the effect of modelling depression on dopamine concentrations in animals. The risk of bias assessment was performed using the SyRCLE RoB tool.

Figure 3.2.1

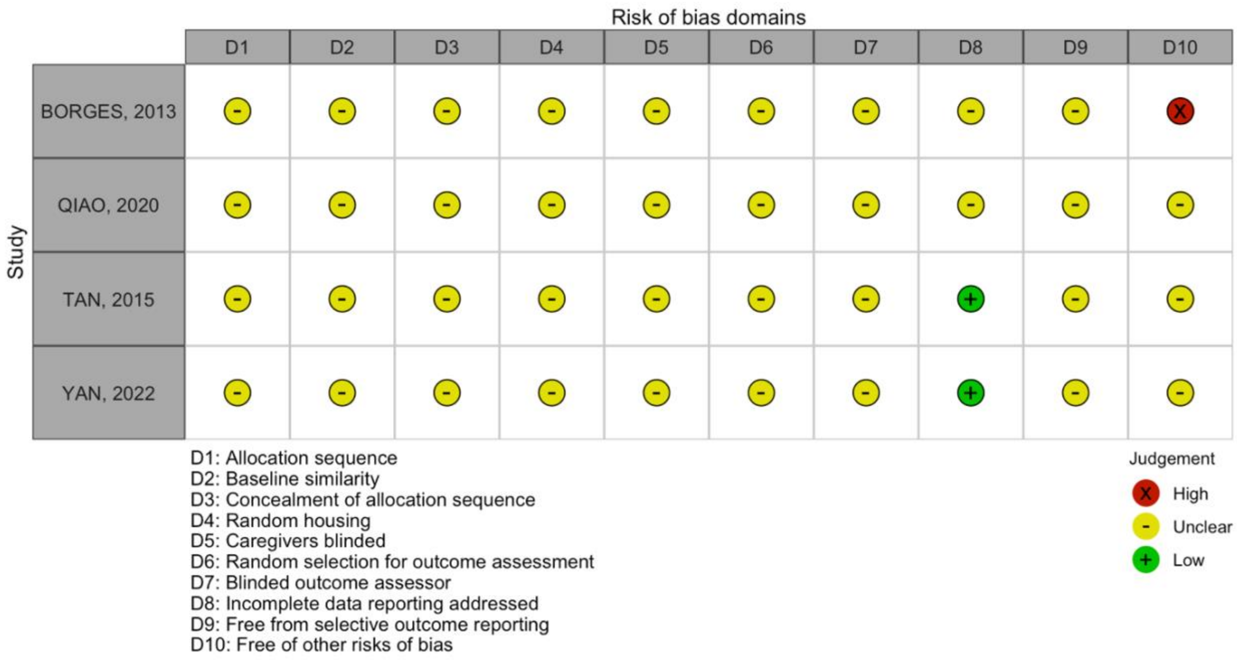

3.2.2 Reporting completeness

Figure 3.2.2 shows the traffic light plot for reporting completeness summary for studies investigating the effect of the modelling of depression on dopamine concentration in animals. The reporting completeness assessment was performed using the ARRIVE guidelines.

Figure 3.2.2

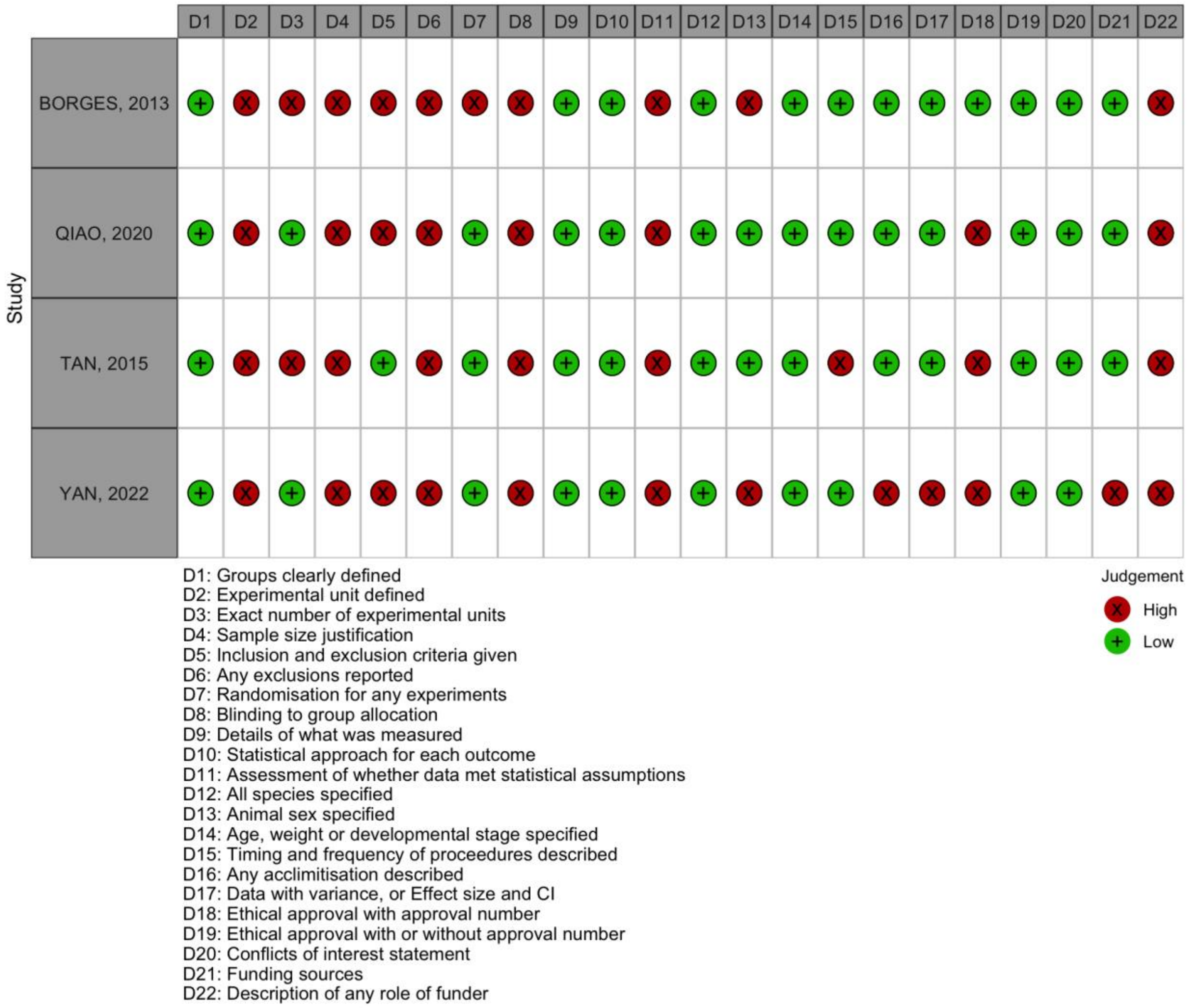

3.2.3 Meta-analysis

Multilevel analysis is only performed if there are 5 levels or more for at least one of Strain, Study and Experiment, and that is not the case here. 7 experimental comparisons were reported in 4 experiments reported from 4 publications and involving 2 different animal strains.

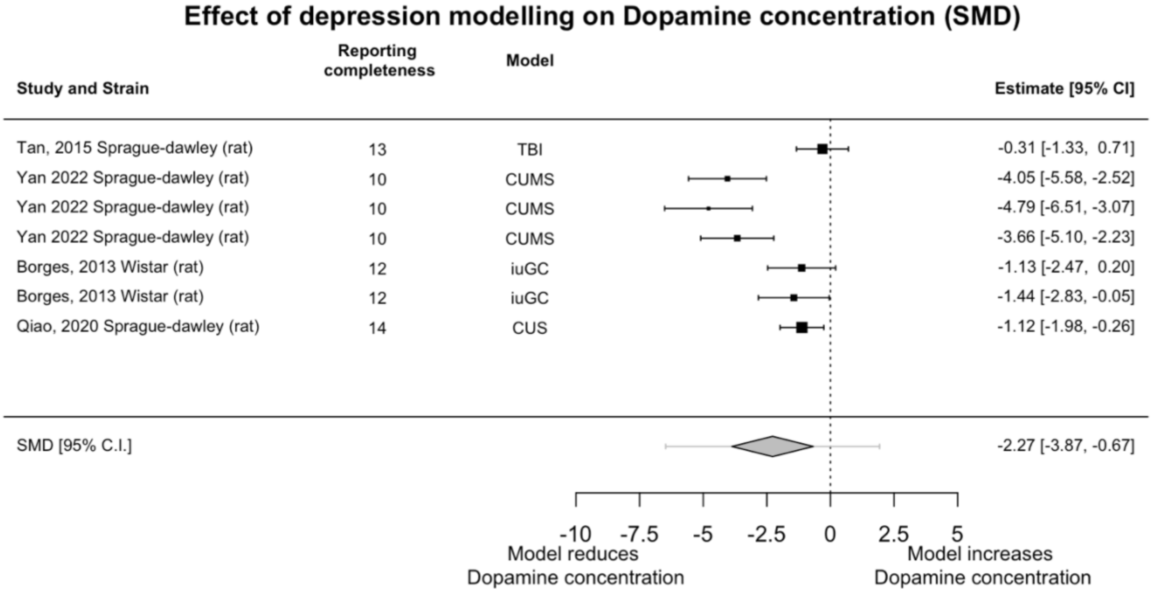

Dopaminergic agents had a pooled effect on dopamine concentration of SMD = -2.27 (95% CI: -3.87 to -0.67; 95% PrI: -6.47 to 1.93).

7 experimental comparisons were reported in 4 experiments reported from 4 publications and involving 2 different animal strains.

3.3 Outcome 3: DOPAC concentration

This was only reported in 2 studies, so no further analysis will be performed.

3.4 Outcome 4: Dopamine / DOPAC ratio

This was only reported in 1 study, so no further analysis will be performed.

3.5 Outcome 5: Dopamine receptor biology

This was only reported in 1 study, so no further analysis will be performed.

4. Observed relationships between different outcomes measures in the same cohorts of animals

We selected cohorts where at least one outcome was presented for at least two outcome types. Where there were two or more of the same outcome type within a cohort we calculated a standardised mean difference effect size for that outcome in that cohort, along with its standard error. Where there was a single effect size within a cohort we took the standard error of that effect size. Then, for each pair of outcome measures we plotted the effect sizes for each cohort, and fitted a regression line weighted on the standard error in the outcome measure represented on the x-axis. Outcome measure pairs are coded according to whether they come from model induction studies (red, expectation of worsening anhedonia) or from intervention studies (green, expectation of improvement in anhedonia). The number of experimental comparisons observed from each cohort is reflected in the size of the symbol, and shown in the figure legend.

4.1 Relationship between change in Sucrose preference test and change in measured dopamine concentrations

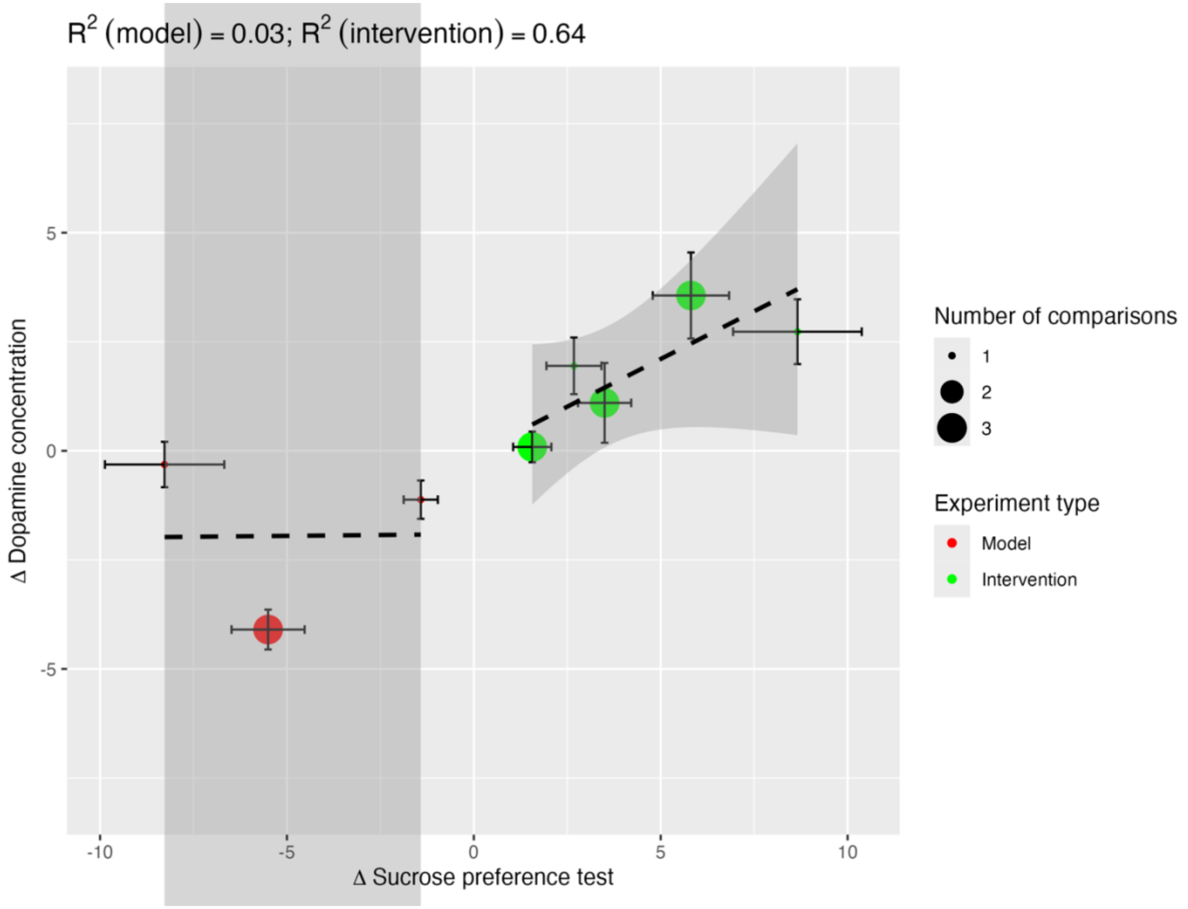

4.2 Relationship between change in Sucrose preference test and change in measured dopamine / DOPAC ratio

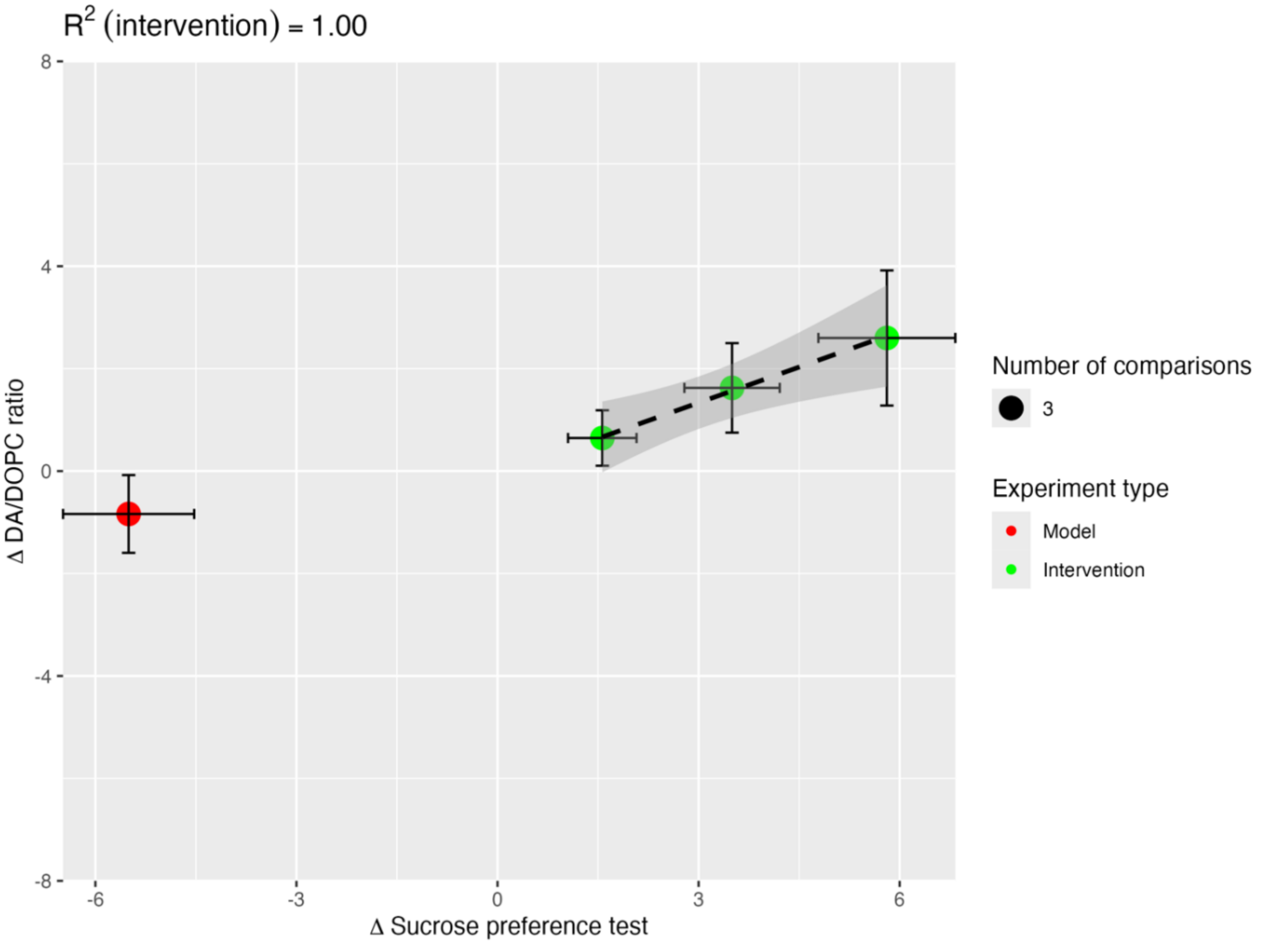

4.3 Relationship between change in dopamine concentrations and change in DOPAC concentrations

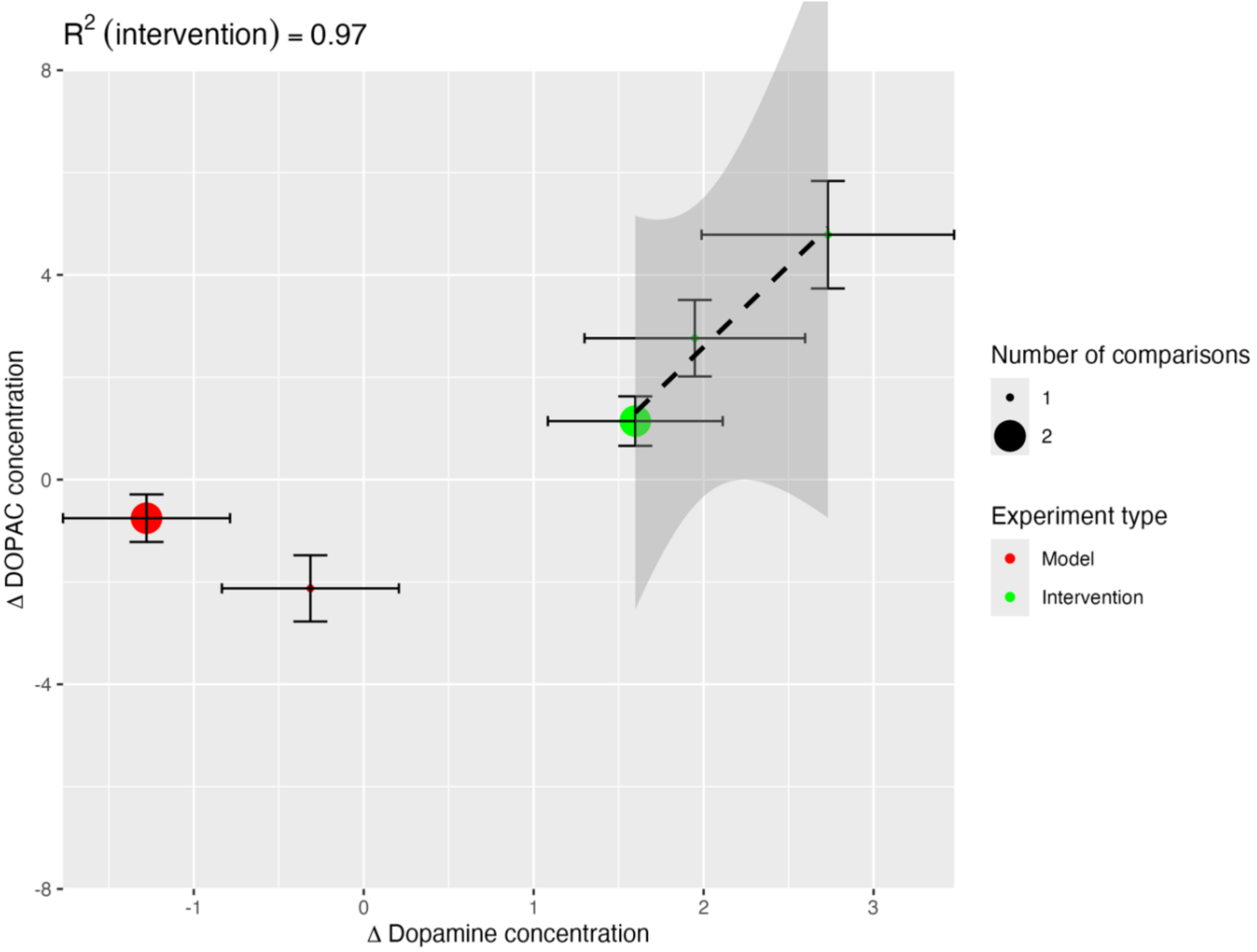

4.4 Relationship between change in dopamine concentrations and change in dopamine/DOPAC ratio

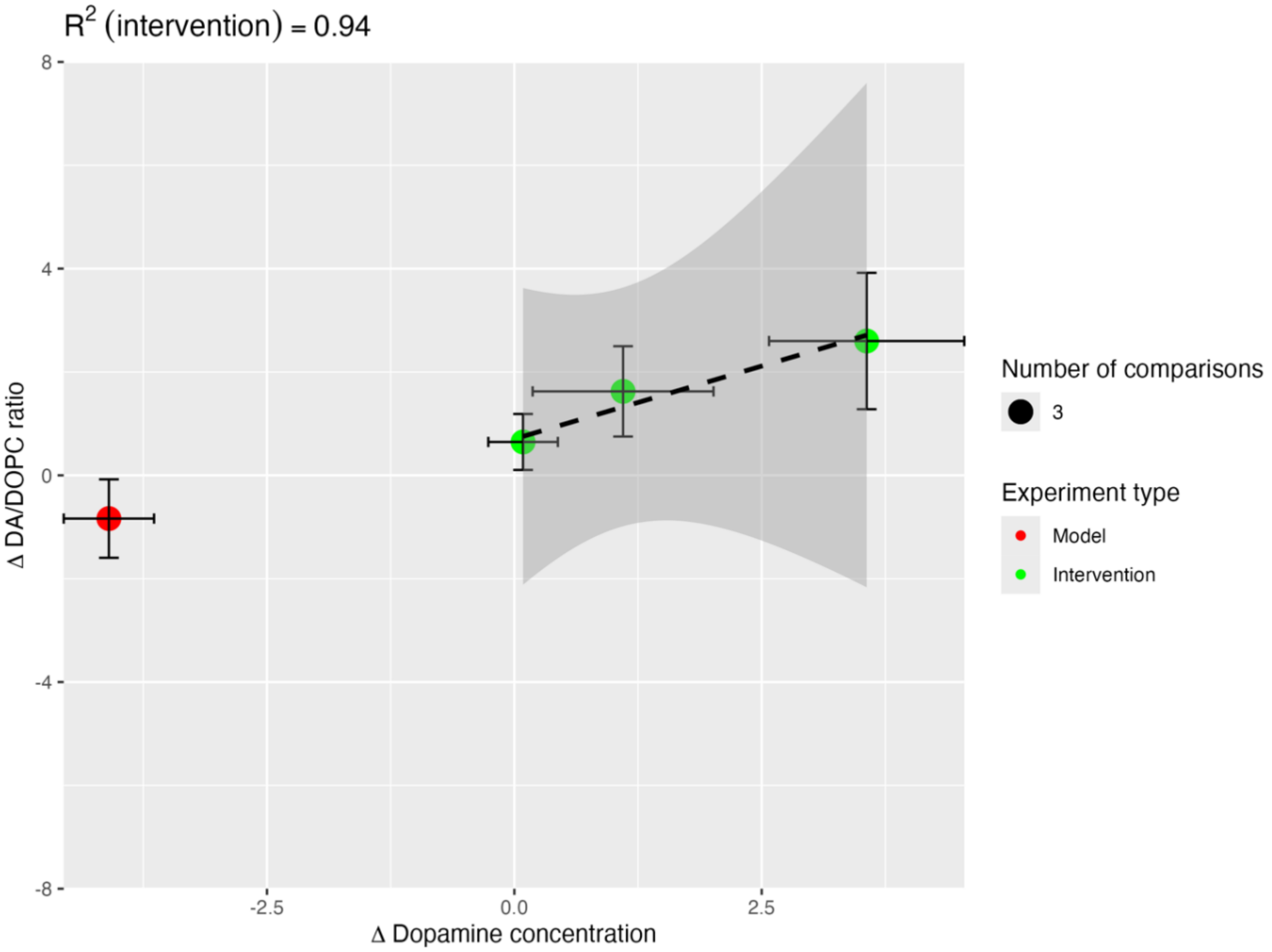

Alt Text

5. Attrition bias and adverse effects of treatment

0% of 793 animals in Control cohorts and 0% of 770 animals in Intervention cohorts ‘dropped out’ between allocation to group and outcome measurement. Given that 10 of 94 interventions (10.64%) were administered as a single dose, treatment emergent adverse effects likely to lead to withdrawal of an animal from the study would be unusual, and technical failure or attrition is more likely. This analysis is based on full reporting of animals excluded from analyses, and it may be that group sizes were specified ‘after the event’, or that there was unreported replacement of animals excluded during the experiment, so these data should be interpreted with caution.

6. Summary of the evidence

6.1 Dopaminergic agents versus control

Outcome	Summary of the association	Within-study biases	Across-studies biases	Indirectness	Other biases
Sucrose preference	64 experimental comparisons from 36 experiments in 27 publications involving 10 animal strains and reporting data from 1156 animals; SMD = 1.34 (95% CI to 0.88 to 1.79; 95% PrI -0.76 to 3.43) (Section 3.1.3). No heterogeneity was observed.	Moderate risk of bias likely to exaggerate the effects of dopaminergic agents. All studies had unclear risk of bias for most of the SyRCLE items. Reporting was mostly incomplete; the median number of ARRIVE items reported was 12 (of 22).	Moderate risk of bias likely to exaggerate the effects of dopaminergic agents. No studies preregistered their analyses. There was evidence of small-study effects (Section 3.1.6).	Moderate risk of indirectness. For explanation, see [1] below.	No other risks identified.
Dopamine concentrations	13 experimental comparisons from 3 experiments in 3 publications involving 2 animal strains and reporting data from 228 animals. There were insufficient data to allow multilevel metaregression; conventional random effects meta-analysis gave SMD	Moderate risk of bias likely to exaggerate the effects of dopaminergic agents. All studies had unclear risk of bias for most of the SyRCLE items. Reporting was mostly incomplete; the median number of	Moderate risk of bias likely to exaggerate the effects of dopaminergic agents. No studies preregistered their analyses. We did not find evidence of small study effects.	Moderate risk of indirectness. For explanation, see [2] below.	No other risks identified.

Outcome	Summary of the association	Within-study biases	Across-studies biases	Indirectness	Other biases
	=1.63 (95% CI: 0.67 to 2.59; 95% PrI -1.69 to 4.95) (Section 3.2.3). We did not explore sources of heterogeneity.	ARRIVE items reported was 10 (of 22).			

6.2 Effect of inducing model, without treatment

To provide context for the effects of dopaminergic drugs in these models, we also present a summary of the effects of model induction.

Outcome	Summary of the association	Within-study biases	Across-studies biases	Indirectness	Other biases
Sucrose preference	42 experimental comparisons from 35 experiments in 26 publications involving 9 animal strains and reporting data from 739 animals; SMD = -2.07 (95% CI: -2.74 to -1.40; 95% PrI -5.06 to 0.92).	Moderate risk of bias likely to exaggerate the effects of anhedonia modelling. All studies had unclear risk of bias for most of the SyRCLE items. Reporting was mostly incomplete; the median number of ARRIVE items reported was 13 (of 22).	Moderate risk of bias likely to exaggerate the effects of anhedonia modelling. No studies preregistered their analyses. There was no evidence for small-study effects.	n.a.	No other risks identified.

Outcome	Summary of the association	Within-study biases	Across-studies biases	Indirectness	Other biases
Dopamine concentrations	7 experimental comparisons from 4 experiments in 4 publications involving 3 animal strains and reporting data from 119 animals. There were insufficient data to allow multilevel metargeression; conventional random effects meta-analysis gave SMD = -2.27 (95% CI: -3.87 to -0.67; 95% PrI -6.47 to 1.93) (Section 2.2.3). We did not explore sources of heterogeneity.	The included study was at unclear risk of bias (SyRCLE); the number of ARRIVE items reported was 13 (of 22).	Moderate risk of bias likely to exaggerate the effects of Dopaminergic agents. The study did not preregistered its analyses.	n.a.	No other risks identified.

Rationale for conclusions for indirectness: **[1] Effect of dopaminergic agents on sucrose preference: Moderate risk of indirectness** We had concerns for indirectness because all experiments were in rodents, and beacuse anhedonia (evaluated using the sucrose preference test) was not identified in the JLA depression Priority Setting Partnership 'Top 10'. However, anhedonia is a well recognised feature of human disease.There were a range of models used including pharmacological, behavioural and 'surgical' (modelling stroke or traumatic brain injury) which are each related to the causation or triggering of depression in humans, and some of these models recapitulate the change in hippocampal volume observed in human depression. Known antidepressants have been reported to increase sucrose preference in animal models of depression, and the depressive phenotype of the WAG/RiJ rat is responsive to antidepressant medication.

[2] Effect of dopaminergic agents on dopamine concentrations: Moderate risk of indirectness In addition to the concerns under [1] above, the concentration of dopamine is not a clinical endpoint, so is therefore less direct.

Evaluation of indirectness of evidence (based on criteria in document "Assessing the certainty of evidence in animal studies") for the studies included in the review

The framework for the evaluation of indirectness is based on eight dimensions, based on the work of Belzung and Lemoine, and comprising **(i) Homological validity** - what is the extent of homology between the model organism and humans relevant to the condition studied; **(ii) Ontopathogenic validity** - Does the model include prenatal or early life exposures inducing transition from initial organism to vulnerable organism; **(iii) Triggering validity** - are any triggering factors used in the modelling – or their homologues -known to induce psychosis or relapse in humans?; **(iv) Mechanistic validity** - whether the neurobiological or cognitive mechanisms which operate in human disease can be observed in the animal model; **(v) Induction validity** - Does the induction of the disease model induce changes in biomarkers (see below) which are known to be altered in human disease?; **(vi) Remission validity** - What is the effect of other drugs known to be effective in humans in the particular animal model / outcome measure pair? ; **(vii) Biomarker validity** - are changes in disease markers (eg neurotransmitter levels, structural brain imaging) seen in human disease also seen in this animal model?; and **(viii) Ethological validity** - what is the 'behavioural distance' between the model phenotype in animals and the symptoms and signs of human disease at which treatment is targeted?

**Dimension**	**Characteristic**	**Homological validity**	**Ontopathogenic validity**	**Triggering validity**	**Mechanistic validity**	**Induction validity**	**Remission validity**	**Biomarker validity**	**Ethological validity**
Species and strain	Rat, Mouse	We could find no evidence that the rat behavioural repertoire is closer to human than is the mouse	n.a.	n.a.	n.a.	n.a.	n.a.	n.a.	n.a.

Dimension	Characteristic	Homological validity	Ontopathogenic validity	Triggering validity	Mechanistic validity	Induction validity	Remission validity	Biomarker validity	Ethological validity
Model Induction	Models using genetic induction – the WAG Riij model	The WAG/RiJ rat is an inbred strain which manifests features of absence epilepsy and comorbid depression.	No	n.a.	Rats manifest abnomalities in DA-ergic and 5HT systems similar to those seen in depression	n.a.	Known antidepressant drugs improve depressive behaviours in WAG/Rj rats	n.a.	n.a.
~	Pharmacological models (Tetrabenazine)	n.a.	No	NA	Tetrabenazine depletes central dopamine stores	Tetrabenazine causes depressive symptomatology in humans	NA	NA	n.a.
NA	Behavioural Models	n.a.	exposure to adversity in early life is associated with depression in adulthood	exposure to adversity is known to trigger episodes of depression	NA	NA	NA	hippocampal volume is reduced in CUMS and in human depression	NA

Dimension	Characteristic	Homological validity	Ontopathogenic validity	Triggering validity	Mechanistic validity	Induction validity	Remission validity	Biomarker validity	Ethological validity
NA	Surgical Models	n.a.	No	The models used were of stroke (MCAO) combined with spatial restraint stress; and of traumatic brain injury. In humans, both of these are associated with depression.	NA	considered'multi-factorial; may be a general effect of adversity	n.a.	hippocampal volume is reduced following experimental TBI; no evidence in MCAO other than direct effect of infact	NA
Outcome Measure	Sucrose preference test	n.a.	n.a.	n.a.	n.a.	n.a.	Desmethylimipramine increases sucrose preference in chronic unpredictable mild stress	n.a.	Anhedonia is not listed on the JLA depression PSP top 10, and

Dimension	Characteristic	Homological validity	Ontopathogenic validity	Triggering validity	Mechanistic validity	Induction validity	Remission validity	Biomarker validity	Ethological validity
							(10.1007/BF00187257)		so the ethological validity of these measures as relevant to unmet clinical need is uncertain
~	Dopamine concentration	n.a.	n.a.	n.a.	reduced [DA] is argued to lead to the reduced dopamine transporter levels reported in human depression	DA depletion causes depression in humans	CUMS increases [DA] in nucleus accumbens, and this is reversed by fluoxetine	n.a.	NA

The description of the criteria is available at https://doi.org/10.17605/OSF.IO/TDMAU

Evaluation of the concordance between different outcome measures

For both the induction of the disease model and the effects of dopaminergic interventions, there was some agreement between the effect sizes measured using these different outcomes when applied to the same cohort of animals. The data are however too sparse to allow firm conclusions at the level of outcome measure pairs.

7. Software used

We used R version 4.3.1 (R Core Team 2023) and the following R packages: devtools v. 2.4.5 (Wickham et al. 2022), dosresmeta v. 2.0.1 (Crippa and Orsini 2016), ggpubr v. 0.6.0 (Kassambara 2023), gtools v. 3.9.5 (Warnes et al. 2023), Hmisc v. 5.1.1 (Harrell Jr 2023a), kableExtra v. 1.4.0.3 (Zhu 2024), knitr v. 1.45 (Xie 2014, 2015, 2023), Matrix v. 1.6.5 (Bates, Maechler, and Jagan 2024), meta v. 7.0.0 (Balduzzi, Rücker, and Schwarzer 2019), metadat v. 1.2.0 (White et al. 2022), metafor v. 4.6.0 (Viechtbauer 2010), mvmeta v. 1.0.3 (Gasparrini, Armstrong, and Kenward 2012), numDeriv v. 2016.8.1.1 (Gilbert and Varadhan 2019), orchaRd v. 2.0 (Nakagawa et al. 2023), patchwork v. 1.2.0 (Pedersen 2024), PRISMA2020 v. 1.1.1 (Haddaway et al. 2022), rje v. 1.12.1 (Evans 2022), rms v. 6.7.1 (Harrell Jr 2023b), robvis v. 0.3.0.900 (McGuinness and Higgins 2020), tidyverse v. 2.0.0 (Wickham et al. 2019), usethis v. 2.2.3 (Wickham et al. 2024), xtable v. 1.8.4 (Dahl et al. 2019).

References

1. Balduzzi S, Rücker G, Schwarzer G. "How to Perform a Meta-Analysis with R: A Practical Tutorial." *Evidence-Based Mental Health 2019*;22:153–60.
2. Bates, Douglas, Martin Maechler, and Mikael Jagan. 2024. *Matrix: Sparse and Dense Matrix Classes and Methods*. https://CRAN.R-project.org/package=Matrix.
3. Bymaster F, Skolnick P, Huang NY, Bradshaew M, McKinney A, Manthis J, Fava M, Tran P. Efficacy and safety of EB-1010, a triple reuptake inhibitor, in the treatment of patients with major depressive disorder. Biol Psychiatry. 2011;69:1S-290S.
4. Crippa A, Orsini N. "Multivariate Dose-Response Meta-Analysis: The dosresmeta R Package." *Journal of Statistical Software* 2016;72:1–15.
5. Dahl, David B., David Scott, Charles Roosen, Arni Magnusson, and Jonathan Swinton. 2019. *xtable: Export Tables to LaTeX or HTML*. https://CRAN.R-project.org/package=xtable.
6. Evans, Robin. 2022. *rje: Miscellaneous Useful Functions for Statistics*. https://CRAN.R-project.org/package=rje.
7. Gasparrini, A., B. Armstrong, and M. G. Kenward. 2012. "Multivariate Meta-Analysis for Non-Linear and Other Multi-Parameter Associations." *Statistics in Medicine* 31 (29): 3821–39.
8. Gilbert, Paul, and Ravi Varadhan. 2019. *numDeriv: Accurate Numerical Derivatives*. https://CRAN.R-project.org/package=numDeriv.
9. Haddaway, Neal R, Matthew J Page, Chris C Pritchard, and Luke A McGuinness. 2022. "PRISMA2020: An r Package and Shiny App for Producing PRISMA 2020-Compliant Flow Diagrams, with Interactivity for Optimised Digital Transparency and Open Synthesis." *Campbell Systematic Reviews* 18 (2): e1230. https://doi.org/10.1002/cl2.1230.
10. Harrell Jr, Frank E. 2023a. *Hmisc: Harrell Miscellaneous*. https://CRAN.R-project.org/package=Hmisc.
11. ———. 2023b. *rms: Regression Modeling Strategies*. https://CRAN.R-project.org/package=rms.
12. Hewett K, Chrzanowski W, Schmitz M, Savela A, Milanova V, Gee M, Krishen A, Millen L, Leary MO, Modell J. Eight-week, placebo-controlled, double-blind comparison of the antidepressant efficacy and tolerability of bupropion XR and venlafaxine XR. J Psychopharmacol. 2009 Jul;23(5):531-8. doi: 10.1177/0269881108089602. Epub 2008 Jul 17. PMID: 18635695.
13. Hewett K, Gee MD, Krishen A, Wunderlich HP, Le Clus A, Evoniuk G, Modell JG. Double-blind, placebo-controlled comparison of the antidepressant efficacy and tolerability of bupropion XR and venlafaxine XR. J Psychopharmacol. 2010a Aug;24(8):1209-16. doi: 10.1177/0269881109106953. Epub 2009 Nov 25. PMID: 19939870.
14. Hewett K, Chrzanowski W, Jokinen R, Felgentreff R, Shrivastava RK, Gee MD, Wightman DS, O'Leary MC, Millen LS, Leon MC, Briggs MA, Krishen A, Modell JG. Double-blind, placebo-controlled evaluation of extended-release bupropion in elderly patients with major depressive disorder. J Psychopharmacol. 2010b Apr;24(4):521-9. doi: 10.1177/0269881108100254. Epub 2009 Jan 22. PMID: 19164492.

15. Jefferson JW, Rush AJ, Nelson JC, VanMeter SA, Krishen A, Hampton KD, Wightman DS, Modell JG. Extended-release bupropion for patients with major depressive disorder presenting with symptoms of reduced energy, pleasure, and interest: findings from a randomized, double-blind, placebo-controlled study. J Clin Psychiatry. 2006 Jun;67(6):865-73. doi: 10.4088/jcp.v67n0602. PMID: 16848645.
16. Kassambara, Alboukadel. 2023. *ggpubr: "ggplot2" Based Publication Ready Plots*. https://CRAN.R-project.org/package=ggpubr.
17. Koshino Y, Bahk WM, Sakai H, Kobayashi T. The efficacy and safety of bupropion sustained-release formulation for the treatment of major depressive disorder: a multi-center, randomized, double-blind, placebo-controlled study in Asian patients. Neuropsychiatr Dis Treat. 2013;9:1273-80. doi: 10.2147/NDT.S48158. Epub 2013 Aug 28. PMID: 24039429; PMCID: PMC3770623.
18. McGuinness, Luke A, and Julian PT Higgins. 2020. "Risk-of-Bias VISualization (Robvis): An r Package and Shiny Web App for Visualizing Risk-of-Bias Assessments." *Research Synthesis Methods*. https://doi.org/10.1002/jrsm.1411.
19. Nakagawa, Shinichi, Malgorzata Lagisz, Rose E. O'Dea, Patrice Pottier, Joanna Rutkowska, Alistair M. Senior, Yefeng Yang, and Daniel W. A. Noble. 2023. "orchaRd 2.0: An r Package for Visualizing Meta-Analyses with Orchard Plots." *EcoEvoRxiv* 12: 4–12. https://doi.org/https://doi.org/10.32942/X2QC7K.
20. Pedersen, Thomas Lin. 2024. *patchwork: The Composer of Plots*. https://CRAN.R-project.org/package=patchwork.
21. R Core Team. 2023. *R: A Language and Environment for Statistical Computing*. Vienna, Austria: R Foundation for Statistical Computing. https://www.R-project.org/.
22. Tran P, Bradshaw M, McKinney A, Bymaster F, Skolnick P, Czobor P, Huang N, Manthis J, Fava M. P.2.c.003 A placebo-controlled study of EB1010, a novel triple re-uptake inhibitor, in patients with major depressive disorder. European Neuropsychopharmacology 2011;10.1016/S0924-977X(11)70617-4.
23. Tran P, Skolnick P, Czobor P, Huang NY, Bradshaw M, McKinney A, Fava M. Efficacy and tolerability of the novel triple reuptake inhibitor amitifadine in the treatment of patients with major depressive disorder: a randomized, double-blind, placebo-controlled trial. J Psychiatr Res. 2012 Jan;46(1):64-71. doi: 10.1016/j.jpsychires.2011.09.003. Epub 2011 Sep 16. PMID: 21925682.
24. Viechtbauer, Wolfgang. 2010. "Conducting Meta-Analyses in R with the metafor Package." *Journal of Statistical Software* 36 (3): 1–48. https://doi.org/10.18637/jss.v036.i03.
25. Warnes, Gregory R., Ben Bolker, Thomas Lumley, Arni Magnusson, Bill Venables, Genei Ryodan, and Steffen Moeller. 2023. *gtools: Various r Programming Tools*. https://CRAN.R-project.org/package=gtools.
26. White, Thomas, Daniel Noble, Alistair Senior, W. Kyle Hamilton, and Wolfgang Viechtbauer. 2022. *metadat: Meta-Analysis Datasets*. https://CRAN.R-project.org/package=metadat.
27. Wickham, Hadley, Mara Averick, Jennifer Bryan, Winston Chang, Lucy D'Agostino McGowan, Romain François, Garrett Grolemund, et al. 2019. "Welcome to the tidyverse." *Journal of Open Source Software* 4 (43): 1686. https://doi.org/10.21105/joss.01686.

28. Wickham, Hadley, Jennifer Bryan, Malcolm Barrett, and Andy Teucher. 2024. *usethis: Automate Package and Project Setup*. https://CRAN.R-project.org/package=usethis.
29. Wickham, Hadley, Jim Hester, Winston Chang, and Jennifer Bryan. 2022. *devtools: Tools to Make Developing r Packages Easier*. https://CRAN.R-project.org/package=devtools.
30. Xie, Yihui. 2014. "knitr: A Comprehensive Tool for Reproducible Research in R." In *Implementing Reproducible Computational Research*, edited by Victoria Stodden, Friedrich Leisch, and Roger D. Peng. Chapman; Hall/CRC.
31. ———. 2015. *Dynamic Documents with R and Knitr*. 2nd ed. Boca Raton, Florida: Chapman; Hall/CRC. https://yihui.org/knitr/.
32. ———. 2023. *knitr: A General-Purpose Package for Dynamic Report Generation in r*. https://yihui.org/knitr/.
33. Zhu, Hao. 2024. *kableExtra: Construct Complex Table with "kable" and Pipe Syntax*.
